# The Secure Anonymised Information Linkage databank Dementia e-cohort (SAIL-DeC)

**DOI:** 10.23889/ijpds.v5i1.1121

**Published:** 2020-02-25

**Authors:** C Schnier, T Wilkinson, A Akbari, C Orton, K Sleegers, J Gallacher, RA Lyons, CLM Sudlow

**Affiliations:** 1 Usher Institute of Population Health Sciences and Informatics, University of Edinburgh, Edinburgh, UK; 2 Centre for Clinical Brain Sciences, University of Edinburgh, Edinburgh, UK; 3 Health Data Research UK Wales and Northern Ireland, Swansea University, Swansea, UK; 4 Administrative Data Research Partnership Wales, Swansea University, Swansea, UK; 5 Center for Molecular Neurology, University of Antwerp, Antwerp, Belgium; 6 Department of Psychiatry, University of Oxford, Oxford, UK; 7 National Centre for Population Health and Wellbeing Research, Swansea University, Swansea, UK; 8 Health Data Research UK Scotland, University of Edinburgh, Edinburgh, UK

## Abstract

**Introduction:**

The rising burden of dementia is a global concern, and there is a need to study its causes, natural history and outcomes. The Secure Anonymised Information Linkage (SAIL) Databank contains anonymised, routinely-collected healthcare data for the population of Wales, UK. It has potential to be a valuable resource for dementia research owing to its size, long follow-up time and prospective collection of data during clinical care.

**Objectives:**

We aimed to apply reproducible methods to create the SAIL dementia e-cohort (SAIL-DeC). We created SAIL-DeC with a view to maximising its utility for a broad range of research questions whilst minimising duplication of effort for researchers.

**Methods:**

SAIL contains individual-level, linked primary care, hospital admission, mortality and demographic data. Data are currently available until 2018 and future updates will extend participant follow-up time. We included participants who were born between 1st January 1900 and 1st January 1958 and for whom primary care data were available. We applied algorithms consisting of International Classification of Diseases (versions 9 and 10) and Read (version 2) codes to identify participants with and without all-cause dementia and dementia subtypes. We also created derived variables for comorbidities and risk factors.

**Results:**

From 4.4 million unique participants in SAIL, 1.2 million met the cohort inclusion criteria, resulting in 18.8 million person-years of follow-up. Of these, 129,650 (10%) developed all-cause dementia, with 77,978 (60%) having dementia subtype codes. Alzheimer’s disease was the most common subtype diagnosis (62%). Among the dementia cases, the median duration of observation time was 14 years.

**Conclusion:**

We have created a generalisable, national dementia e-cohort, aimed at facilitating epidemiological dementia research.

## Introduction

Dementia is a major global health challenge [1,2] and the current lack of disease-modifying therapies places the onus on the research community to identify potentially modifiable risk factors [3], as well as to study its incidence, prevalence and natural history. The pathologies underlying neurodegenerative diseases such as Alzheimer’s disease are likely to begin many years before symptom onset [4], and so long follow-up times are required to determine whether an association between a given factor and dementia is truly causal or due to reverse causation [5,6]. Longitudinal studies, with prospective data collection, are therefore of great importance to improving our understanding of dementia.

Dementias Platform UK (DPUK, www.dementiasplatform.uk) is a UK-wide, public-private partnership, that aims to facilitate and accelerate dementia research by providing a single point of access to data for >2 million participants across >38 existing cohort studies. Many of these cohorts have provided, and will continue to provide, important insights in the field of dementia. However, as these cohorts require participant consent for recruitment, they are likely to suffer from selection bias [7–9]. In contrast, a nationwide cohort based on whole-population administrative data is likely to avoid this issue, as analyses based on it can be more readily generalised to other populations.

The Secure Anonymised Information Linkage (SAIL) Databank (https://saildatabank.com) is a remotely-accessible, privacy-protecting data safe haven containing anonymised, individual-level, linked routinely-collected health and social care datasets for the population of Wales, UK [10–12]. Wales, with a population of approximately three million people, is one of four countries in the UK. A key enabler of data creation is Wales’ National Health Service (NHS), which acts a single provider of healthcare, free at the point of use to the resident population. As a result, SAIL is a large, nationwide, population-based research resource comprising longitudinal, routinely-collected healthcare data. Its size, national coverage and richness of available data means SAIL has the potential to be of great value for dementia research [13]. However, it can be a demanding task for researchers to transform the complex and varied datasets into a study population appropriate for their research question, as well as to identify participants with dementia with a minimum of misclassification.

By applying coding algorithms to linked routinely-collected datasets, we developed a novel DPUK cohort – the SAIL Dementia e-Cohort (SAIL-DeC). SAIL-Dec is a population-based electronic cohort (e-cohort) containing health-related information on people with and without diagnosed dementia. We developed SAIL-DeC to maximise its generalisability and utility for a broad range of research questions and methodologies. For example, we anticipate SAIL-DeC data being used to conduct risk factor studies, explore geographical variations in dementia incidence or outcomes, develop or validate risk prediction models and perform health economic analyses.

We created SAIL-DeC with the aims of minimising duplication of effort, increasing reproducibility, reducing costs and allowing a broader range of researchers to apply to use SAIL data.

## Methods

### Study reporting

We have followed the Reporting of studies Conducted using Observational Routinely-collected Data (RECORD) guideline in formatting this manuscript [14]. The SQL script used to create the cohort and cohort meta-data are available at https://datashare.is.ed.ac.uk/handle/10283/3268.

### SAIL Databank

The SAIL Databank, based at Swansea University, was developed based on four principles: (1) to operate a remote access system, providing secure access to data to approved researchers; (2) to provide a powerful data analytic platform; (3) to ensure a robust mechanism for the safe transfer of approved files in and out of the system; and (4) to be efficient and scalable [11].

SAIL uses a split-file anonymisation method to maintain confidentiality. Individuals within each routinely-collected dataset are assigned a unique identifier (Anonymised Linking Field [ALF]). The ALF is generated by NHS Wales Information Service, a trusted third party, using the Matching Algorithm for Consistent Results in Anonymised Linkage, which has an accuracy of 99.85% [12,13]. Within SAIL, the ALF is further encrypted (ALF-E) and used to link the now de-identified individuals across multiple routinely-collected datasets, with further encryption (ALF_PE) then applied before data are allocated to an approved project.

### Datasets

To construct SAIL-DeC, we used linked primary care, hospital admissions, mortality and deprivation datasets (https://saildatabank.com/saildata/sail-datasets). Hospital admissions data (Patient Episode Database for Wales [PEDW]), first collected in Wales in April 1991, contain information regarding inpatient admissions (emergency, elective and maternity) and day-case procedures. Diagnoses within PEDW are coded using the International Classification of Diseases version 10 (ICD-10) system [15]. Mortality data (Annual District Death Extract [ADDE]), available in SAIL since 1995 and derived from England and Wales’ death certification and registration system, contain diagnoses of cause of death as well as contributory comorbidities. ADDE uses ICD-9 coding until 2001 [16], and ICD-10 coding thereafter. Primary care data (Welsh Longitudinal General Practice dataset [WLGP]), currently use the Read version 2 system [17,18], although this will ultimately be replaced with the international SNOMED CT system in the future [19]. While ICD-10 codes contain only diagnostic information, Read codes contain information on diagnoses, administrative procedures, prescriptions, and symptoms and signs, making them a potentially rich resource for a wide range of research. Currently, SAIL contains primary care data for approximately 80% of the Welsh population. The subpopulation for whom primary care data are available are representative of the entire Welsh population in terms of age, sex and deprivation (Supplementary Appendix 1). The period of time covered by primary care data varies considerably between individuals and across practices – we included all available primary care data for all eligible participants. Deprivation data were derived from the Welsh Demographic Service Dataset (WDSD). Within WDSD, the Welsh Index of Multiple Deprivation [WIMD], is used to measure relative deprivation based on geographical household location, for small areas in Wales (Lower-layer Super Output Areas [LSOAs])[20]. To create SAIL-DeC we used the 2011 version of WIMD, linked to the 2001 version of LSOAs. In future updates of the cohort we will be able to update these deprivation datasets as new versions become available.

### Study population

We included all participants within the SAIL Databank for whom primary care data were available, based on being registered with a SAIL-contributing general practice (GP) at any point. We excluded participants with a date of birth listed as before 1^st^ January 1900, as we deemed these to be likely to be incorrect. We also excluded participants whose 60^th^ birthday would fall after the latest date of follow-up, because the prevalence of dementia is very low below this age [21]. We therefore included participants born between 1^st^ January 1900 and 1^st^ January 1958 in the initial cohort development. The later date will change as the SAIL Databank receives updates of the datasets in the future, meaning the cohort will continue to increase in size over time as more participants become eligible. The timing of cohort refreshes will be negotiated with prospective applicants and available updates added to relevant extracts within SAIL-DeC.

We defined the entry date into the cohort as the first date of registration with a SAIL GP. We excluded participants without a valid GP registration date. We defined the last date of follow-up as the earliest of GP de-registration or death (currently January 2018).

### Cohort tables

**Figure 1: Format of SAIL dementia e-cohort:**
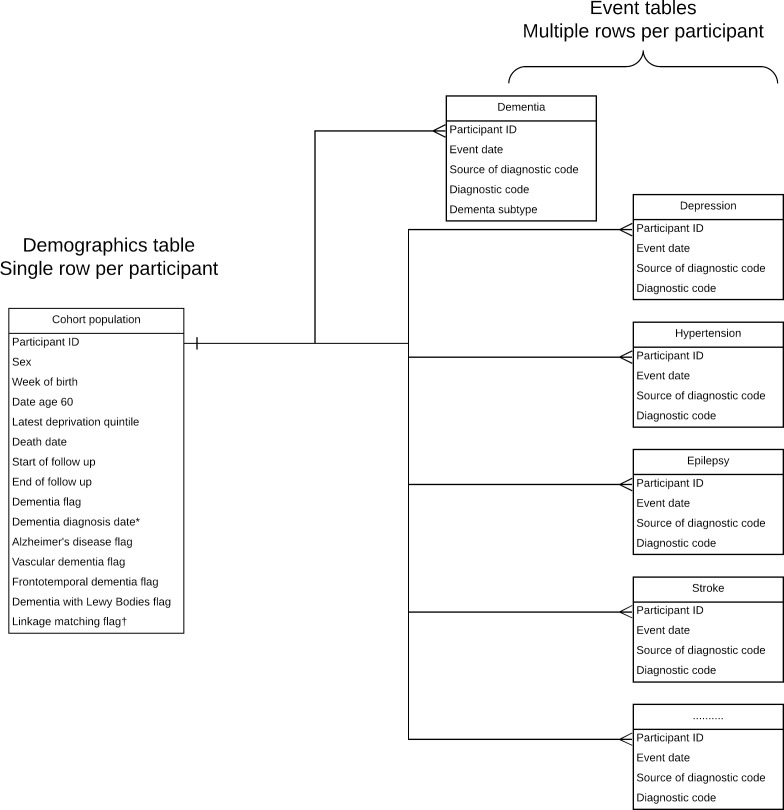
Event tables consist of derived variables for: ever-smoking; obesity; hypertension; atrial fibrillation; peripheral arterial disease; myocardial infarction; stroke; diabetes; asthma; cancer; chronic obstructive pulmonary disease; depression; epilepsy; heart failure; hypothyroidism; osteoporosis; alcohol dependence; substance misuse; motor neurone disease; Parkinson’s disease; and rheumatoid arthritis. *field blank if participant did not develop dementia. †Flag indicating that linkage to ≥ 1 datasets for this participant may not be accurate (<95% probabilistic matching).

Using information from the four datasets, we created three types of table (Figure 1):

1. A demographics table, with one row per participant. This table holds basic demographic information and information on the follow-up time to allow survival analysis and/or efficient case-control matching. This table also contains information on the death date if appropriate and indicator flags on whether the participant developed all-cause dementia during follow-up. There is also a flag for whether the participant received a dementia subtype code.
2. A dementia events table, with multiple rows per participant. This contains the code details, date and source (i.e. primary care, hospital admissions or mortality data) of each dementia code and the dementia subtype to which the code refers.
3. Multiple risk factor/comorbidities events tables, with multiple rows per participant. Using the same format as the dementia events table, these tables contain information on the specific code, date and data source for each derived risk factor or comorbidity.

We created a data dictionary, which lists all cohort tables and outlines the source of the derived variables for each table type (Supplementary Appendix 2).

### Derived variables

#### Dementia

We used a validated list of ICD-9, ICD-10 and Read V2 codes to identify all-cause dementia, Alzheimer’s disease, vascular dementia cases, dementia with Lewy Bodies and frontotemporal dementia in primary care, hospital admissions or mortality data (Supplementary Appendix 3). We developed the code list based on findings from a systematic review of the accuracy of dementia coding in routinely-collected healthcare data [22] and a UK-based validation study in which cases identified from coded data were compared to the full-text medical record [23]. We defined the date of diagnosis in the demographics table as the date of the first all-cause dementia code in any dataset. Where a participant had a dementia subtype code (e.g. Alzheimer’s disease), we defined date of diagnosis as the first date of any (all-cause) dementia code. Subtype code categories were not mutually exclusive, so a participant with ≥ 1 Alzheimer’s disease and vascular dementia codes in any dataset would be categorised as having both of these subtype diagnoses.

#### Risk factors and comorbidities

We created ICD-9, ICD-10 and Read V2 code lists to derive variables for risk factors and comorbidities based on a four-stage process: (1) code lists used by existing studies [24–34]; (2) an online clinical codes repository [35]; (3) where available, the recommended Read code lists from the UK Quality Outcomes Framework (QOF) [36] and (4) a manual review of the codes by a clinician (TW) (Supplementary Appendices 4-6). Where possible, we used validated code lists with known accuracy versus a definable reference standard; however, this was not possible for the majority of variables. Researchers who apply to use data from SAIL-DeC can use these code lists, or create their own using the underlying datasets, thereby creating an iterative process in which we use feedback from users of SAIL-DeC to improve and create different versions of the definitions of derived variables over time.

#### Bias

As the SAIL Databank contains data for the entirety of Wales, and primary care data for 80% of the population, we have attempted to minimise selection bias by including all eligible participants. We created code lists for diagnoses with the intention of maximising positive predictive value (PPV, the proportion of identified cases that are true cases) whilst maintaining a reasonable sensitivity (the proportion of true disease cases identified), in order to minimise bias in effect estimates [37].

#### Statistical methods

We calculated the total number of people and number of person-years of follow-up, stratified by sex, deprivation and birth decade for the whole cohort and for the dementia cases.

For the dementia cases, we counted the number of participants who had a specific dementia subtype code (Alzheimer’s disease, vascular dementia, dementia with Lewy Bodies and frontotemporal dementia). We calculated the median duration of follow-up for the dementia cases, as well as the number of cases and follow-up time for dementia cases in whom follow-up began prior to age 60. We also created an event flow diagram, indicating to what extent and in which order dementia cases were identified across multiple datasets. We calculated the number of dementia cases and person-years at risk for each derived risk factor or comorbidity.

### Data access and cleaning methods

To create SAIL-DeC, we accessed all hospital admissions, mortality, primary care and deprivation data contained within SAIL. For the purposes of data cleaning, we excluded:

- Participants with a recorded date of birth before 1/1/1900;
- Any information from mortality data when the date of death was recorded as being before 1/1/1980 or after 1/1/2020. Where a record in mortality data was missing but death was recorded in the WDSD dataset, we used the latter to obtain the death date (7% of deceased participants);
- Participants without a GP registration start or end date;
- Participants who had a dementia diagnosis without a valid date (i.e. before 1/1/1900 or after 1/1/2018), as we did not know whether they represented true cases.

SAIL contains information on the linkage quality of ALFs obtained following deterministic and probabilistic matching which have been through a standard split file approach. We did not exclude participants based on low ALF matching rates, but instead created a flag in the demographics table to indicate where a participant has one or more linkages with <95% probabilistic matching. Users of SAIL-DeC can therefore opt to exclude participants of lower linkage quality depending on their study requirements.

## Results

### Demographics of whole cohort and dementia cases

**Figure 2: Study flow diagram:**
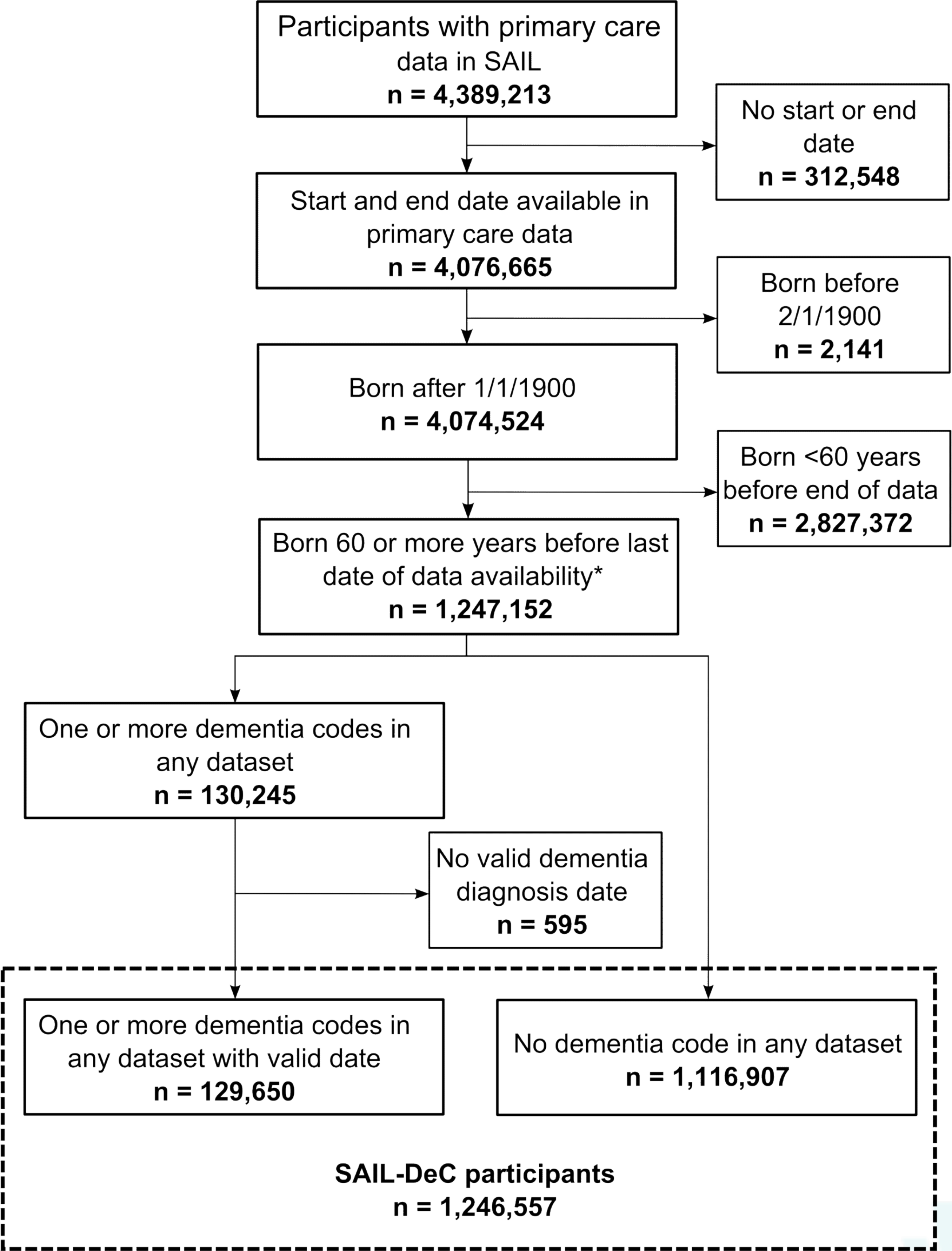
*Currently April 2016

From the 4,389,213 people within the SAIL Databank with primary care data, 1,246,557 participants met the cohort inclusion criteria (Figure 2), resulting in 18,802,369 person-years of follow-up. For the whole cohort, the median first GP registration date was October 1995, with a median age at registration of 59 years. For participants with a diagnosis of dementia, the median date of first GP registration was January 1996 and median age at registration was 71 years. The demographics of the whole cohort and the dementia cases are displayed in Table 1. In the whole cohort, participants were equally distributed across deprivation quintiles.

*Latest deprivation score for each participant

**Demographics of dementia cases and whole cohort:** 

	Whole cohort			Dementia cases		
		
	n	(%)	Person-years	n	(%)	Person-years
Total participants	1,246,557	18,796,117	129,650	1,773,462
Sex
Female	658,518	(53)	9,963,822	82,571	(64)	1,118,479
Male	588,035	(47)	8,832,289	47,079	(36)	654,983
Missing	<5	(0)	5	0	(0)	0
Deprivation quintile*
1 (Most deprived)	224,376	(18)	3,276,397	24,915	(19)	334,806
2	238,021	(19)	3,438,409	26,344	(20)	353,203
3	264,799	(21)	3,939,140	27,798	(21)	377,314
4	242,115	(19)	3,696,385	25,317	(20)	346,736
5 (Least deprived)	255,363	(20)	4,223,548	24,033	(19)	350,798
Missing	21,883	(2)	222,238	1,243	(1)	10,604
Birth Decade
1900-1910	40,380	(3)	231,761	7,633	(6)	51,713
1911-1920	130,388	(10)	1,157,531	32,515	(25)	334,063
1921-1930	228,475	(18)	2,962,413	50,179	(39)	720,464
1931-1940	271,791	(22)	4,451,912	27,922	(22)	470,036
1941-1950	373,971	(30)	6,500,674	9,450	(7)	163,509
1951-1960	201,552	(16)	3,491,826	1,951	(2)	33,677

### Dementia cases

#### Dementia subtypes

**Figure 3: Dementia subtypes:**
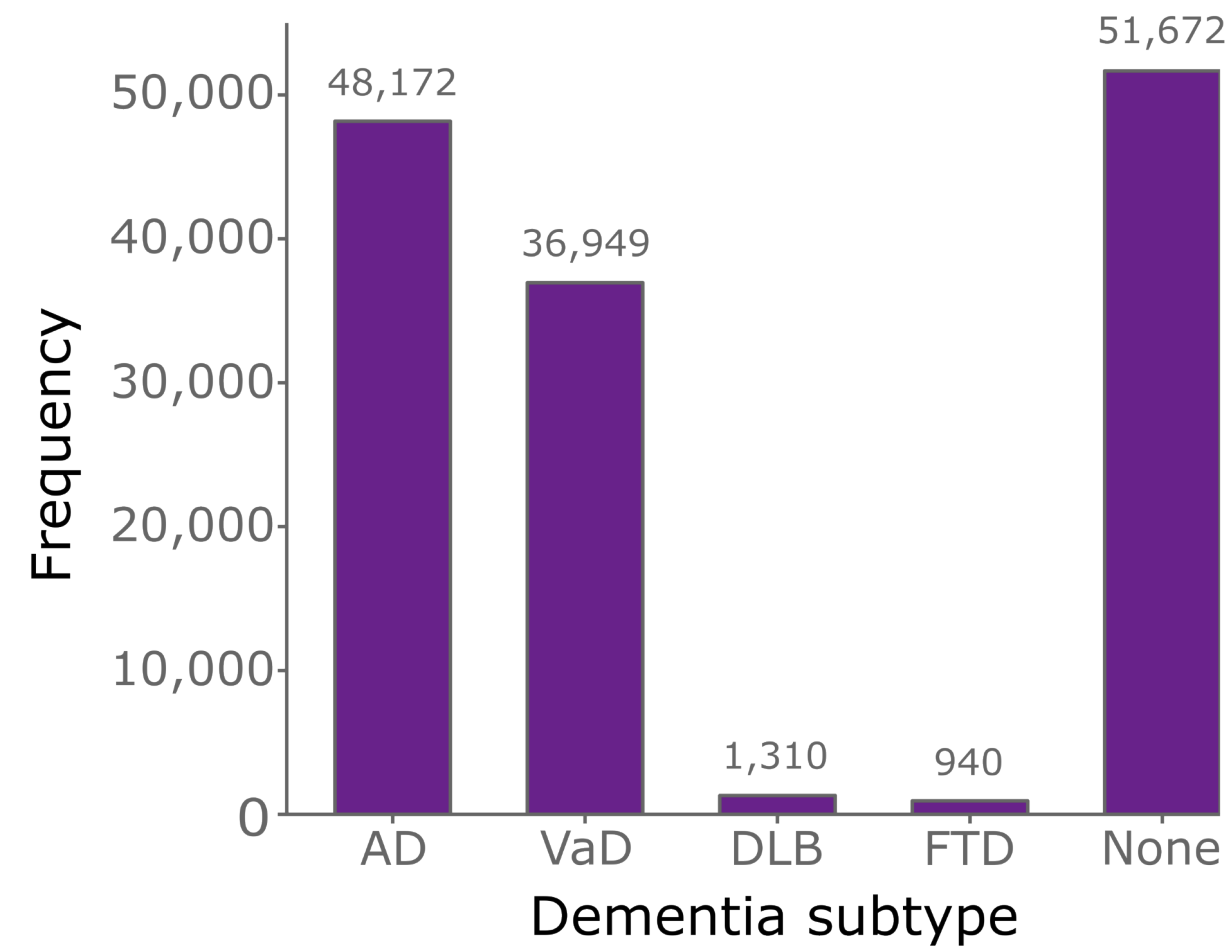
AD – Alzheimer’s disease, VaD – vascular dementia, DLB – dementia with Lewy Bodies, FTD – frontotemporal dementia, None – no subtype code. Categories not mutually exclusive (apart from ‘none’ category). 77,978/129,650 (60.1%) of participants with all-cause dementia had at least one dementia subtype code. 8653 participants had both Alzheimer’s disease and vascular dementia codes (‘mixed dementia’).

Of all SAIL-DeC participants, 129,650 (10%) developed all-cause dementia during follow-up. Of these, 77,978 (60%) had ≥ 1 codes for a dementia subtype (Figure 3). Alzheimer’s disease was the most common subtype diagnosis (48,172, 62%), followed by vascular dementia (36,949, 47%). 8,653 (11%) participants with dementia had both Alzheimer’s disease and vascular dementia codes.

#### Case ascertainment across datasets

From the 129,650 dementia cases, 78,828 (61%) were identified at any time in primary care data, 101,654 (78%) in hospital admissions data and 52,198 (40%) in mortality data. Forty-eight percent of participants were first identified in primary care data, 48% in hospital admissions data and 4% were identified only in mortality data (Supplementary Appendix 7). The number of dementia cases identified by each code are summarised in Supplementary Appendix 8.

#### Observation period

Among dementia cases, the median duration of follow-up was 14 years. We were able to follow up 23,724 (18%) dementia cases from <age 60 years, with a median follow-up time of 22 years. Seventy-nine percent of participants who developed dementia died during follow-up.

#### Risk factors and comorbidities

The number of participants and duration of follow-up for each risk factor or comorbidity among the 129,650 dementia cases is displayed in Table 2. A detailed breakdown of the number of participants identified by individual codes, as well as the extent to which participants with each risk factor or comorbidity are identified in the datasets over time, is available in Supplementary Appendices 9-29 and at https://datashare.is.ed.ac.uk/handle/10283/3268.

**Table 2: Numbers and person-years of follow-up for risk factors or comorbidities across 129,650 dementia cases:** *Chronic kidney disease stages III-V. ICD-9, ICD-10 and Read V2 codes used to derive the variables are displayed in Supplementary Appendices 4-6. A detailed breakdown of the number of participants identified by each individual code is available in Supplementary Appendices 9-29 and at https://datashare.is.ed.ac.uk/handle/10283/3268.

Risk factor / comorbidity	Dementia cases		
	
	n	(%)	Person-years
Ever-smoker	67,775	(52)	1,070,030
Obesity	9,197	(7)	161,496
Hypertension	81,377	(63)	1,211,400
Diabetes mellitus	29,745	(23)	430,198
Osteoporosis	24,486	(19)	381,686
Atrial fibrillation	38,866	(30)	556,328
Myocardial infarction	22,194	(17)	310,403
Heart failure	31,703	(24)	426,072
Peripheral arterial disease	9,612	(7)	136,934
Stroke	32,240	(25)	427,974
Depression	39,130	(30)	587,340
Parkinson’s disease	9,845	(8)	128,771
Epilepsy	9,496	(7)	128,487
Motor neurone disease	332	(0)	4,284
Asthma	22,303	(17)	338,197
Chronic obstructive pulmonary disease	24,444	(19)	346,059
Cancer	34,663	(27)	496,564
Rheumatoid arthritis	5,597	(4)	82,091
Hypothyroidism	18,663	(14)	273,953
Alcohol dependence	6,289	(5)	89,684
Substance misuse	2,323	(2)	36,589
Chronic kidney disease*	26,821	(21)	447,895

## Discussion

We have applied algorithms to routinely-collected primary care, hospital admissions, mortality and deprivation datasets within the SAIL Databank to create a ‘real world’ dementia e-cohort. We have incorporated this cohort into DPUK, to complement existing, ‘consented’ cohort studies within the initiative.

### Flexibility

SAIL-DeC is designed to be flexible, meaning researchers can choose to use the existing disease definitions or create new ones to suit their purposes. For example, we used a validated code list for dementia outcomes in which the presence of a single dementia code in any dataset leads to a participant being identified as a dementia case, with the date of the first dementia code used to determine the ‘date of diagnosis’. Users may wish to use an alternative definition of dementia – for example, requiring >1 dementia codes in any data source, or including prescriptions for dementia drugs (e.g., cholinesterase inhibitors) in the algorithm. Similarly, they may wish to create new comorbidity variables or adapt the existing ones. We included all available data for all eligible participants, allowing users of SAIL-DeC to create a sub-cohort relevant to their research question. Researchers who intend to use SAIL-DeC as a cohort study (e.g. when conducting a case-cohort analysis) will need to select a time point from which follow up starts for an individual, such as a specific date or participant age.

### Identifying dementia cases using routinely-collected healthcare data

For routinely-collected healthcare datasets to be used to identify dementia cases for research, they must do so with sufficient accuracy [22]. If using the data for analyses of risk factors or the natural history of dementia, identifying disease outcomes with a high PPV is important in order to minimise the risk of bias (37). Validation studies of UK routinely-collected healthcare data to identify dementia cases have reported PPVs of 83-100% [23,38,39], 85-87% [23,40] and 80-90% [23,41] for primary care, hospital admissions and mortality data respectively.

The sensitivity (the proportion of true disease cases identified) of using routinely-collected healthcare data to identify disease outcomes is another important consideration. There is a trade-off between PPV and sensitivity, meaning case ascertainment methods with a high PPV may fail to identify a proportion of ‘true’ cases. Studies of the sensitivity of hospital admissions and mortality data in patients known to mental health services with dementia (and therefore likely to overestimate sensitivity as it does not account for the proportion of people with dementia who are undiagnosed) reported estimates of 78% and 54% respectively [42,43]. The sensitivity of UK primary care data is currently unknown [22], although a study is underway to investigate this [44].

The use of multiple data sources improves our understanding of the timing of a dementia diagnosis, as in some cases there can be a significant delay between the identification of a participant with dementia in one dataset compared to others (Supplementary Appendix 7). This is particularly important for analyses in which the date of dementia diagnosis is needed, such as time-to-event analyses.

### Potential uses

There are numerous potential uses for this cohort. Given the breadth of primary care Read codes, there is the opportunity to identify novel risk factors for dementia and its subtypes. For example, there is increasing evidence that some drugs are associated with an increased risk of dementia [45–47]. The primary care dataset within SAIL-DeC contains details of all drug prescriptions, meaning the cohort could be used to explore this issue. Recent work by the Whitehall II study has shown the importance of long follow-up times to explore whether associations between various factors and dementia are in fact due to the effects of dementia on the factor itself (reverse causation) [5,6]. The long follow-up times in SAIL-DeC would enable such studies for a variety of risk factors.

Routinely-collected healthcare datasets have been used to study dementia incidence and prevalence [48,49], as well as to investigate within-country geographical variations in dementia outcomes [50]. As SAIL have obtained primary care data for a large proportion of the Welsh population, with included participants representative of the wider population, the dataset provides an opportunity to explore geographical variations in dementia incidence or outcomes.

Of the participants who developed dementia, 79% died during follow-up. This shows that, for many participants, follow-up until death was ‘completed’ (i.e. not censored early). SAIL-DeC would therefore be well suited to studies surrounding end of life for people diagnosed with dementia.

Primary care data have been used to develop and validate risk prediction models for a range of diseases [51–53]. Given that the variables within SAIL-DeC are all routinely-collected, any variables used in a dementia risk prediction model developed using the cohort’s data would be applicable to current clinical use.

The breadth of information contained within Read codes raises the possibility of using data within the cohort for hypothesis-free studies of dementia, such as environment-wide association studies [54,55]. With >100,000 dementia outcomes within SAIL-Dec, the cohort is likely to provide sufficient statistical power for such analyses.

In addition to hospital admissions data, SAIL also contains other healthcare datasets such as emergency attendances, critical care admissions, care home residence, pathology results and outpatient referrals. Other routine datasets are continuing to be linked to SAIL participants, and these could be linked to SAIL-DeC participants when they are made available. These datasets may provide a useful means with which to conduct health economic analyses for dementia care. There are also plans to derive new phenotype data from multiple sources of NHS clinical data using natural language processing, such as radiology reports and free-text correspondence between clinicians.

### Accessing the data

Researchers interested in using SAIL-DeC data can contact the SAIL Databank directly (https://saildatabank.com/application-process), or approach DPUK via the DPUK portal (https://portal.dementiasplatform.uk), who can facilitate the application. Applicants must submit their proposal to the independent Information Governance Review Panel (IGRP), which ensures proper and appropriate use of SAIL data. Researchers are required to demonstrate appropriate Information Governance training prior to being provided with remote access to the SAIL safe haven [11]. Costs depend on the complexity of the project and support required and are outlined early in the project scoping process. Our intention is that using the pre-prepared SAIL-DeC datasets should reduce the complexity of data preparation, thereby minimising costs and time needed for new studies.

### Strengths and limitations

SAIL-DeC has several strengths as a research resource. It is population-based, and contains data for ~80% of Wales, meaning that it is likely to be generalisable to other similar populations and should not suffer from the ‘healthy cohort effect’ [7–9]. This is reflected in the near-equal distribution of participants across deprivation quintiles. Its size is another strength. With 18.8 million person-years of follow-up in the whole cohort, there is likely to be sufficient power for most types of analyses, even on relatively rare exposures or outcomes. In creating the e-cohort, we have attempted to make it useful for a wide range of research studies, with the aim of increasing efficiency and reducing costs for researchers. We have used coded algorithms to simplify the routinely-collected datasets, with the intention of enabling researchers without experience of using UK healthcare datasets to use SAIL data. We have created derived variables for many comorbidities and risk factors, but the resource is designed to be flexible: users can request additional variables or alter how variables are defined if required for their analyses. Furthermore, if users change how some derived variables are defined (i.e. codes removed or added, or more complex algorithms created) based on their experience or the latest evidence, we can alter the definitions of these variables for other researchers too, creating a ‘learning’ resource for the dementia research community.

The e-cohort also has several limitations. First, routinely-collected healthcare data will not identify dementia cases with perfect accuracy. Whereas validation studies have shown PPV to be generally high across UK datasets, no studies have calculated the sensitivity of using primary care, hospital admissions and mortality data in combination [22]. To have the opportunity to appear in routinely-collected data, people with dementia must first be known to healthcare services with a dementia diagnosis, and dementia is known to be underdiagnosed [56], meaning sensitivity is likely to be lower than PPV. This means that SAIL-DeC would probably not be an appropriate resource with which to calculate absolute dementia prevalence and incidence in Wales as it will likely underestimate the number of cases. However, it could be used to compare the relative burden of dementia across different geographical areas.

The accuracy of the algorithms used to derive many of the variables for risk factors and comorbidities is not known. We have attempted to use algorithms with a presumed high PPV over sensitivity, by including codes we consider likely to reflect true positive cases and excluding codes that may introduce false positive cases. This means that some of our algorithms may identify risk factors and comorbidities with a low sensitivity, as suggested by the low rates of obesity (7%) at any point for the dementia cases. It is likely that some of the factors, particularly those for which recording is mandatory in QOF, are better recorded and therefore more likely to be detected than others. Our intention is that this will improve over time: as new validation studies of these variables are performed, and users of SAIL-DeC provide feedback on the code lists for these variables, we will update these algorithms to maximise their accuracy. For example, there is the potential to create more complex algorithms (e.g. by including continuous measurements such as body mass index for obesity or blood pressure readings for hypertension), to improve case ascertainment for some variables.

Although UK routinely-collected healthcare datasets identify all-cause dementia cases with a high PPV, the PPVs for the identification of dementia subtypes is lower. Using UK hospital admissions, mortality and primary care data in combination, PPVs were estimated as 71% for Alzheimer’s disease and 44% for vascular dementia [23]. There have been no validation studies estimating the PPVs for rare dementia subtypes such as dementia with Lewy Bodies and frontotemporal dementia [22]. Researchers should consider this when using SAIL-DeC to study dementia subtypes rather than all-cause dementia.

Furthermore, SAIL-DeC relies entirely on routinely-collected data to identify dementia cases as well as risk factors and other comorbidities. Although Read coding in primary care data provides a wide range of information in addition to diagnoses such as symptoms, signs, administrative procedures and prescriptions, there is limited phenotypic depth – for example there is no imaging, free-text or genetic data. Over time this may change, as SAIL obtains linkage to more detailed datasets.

The availability of primary care data in SAIL increased over time, as practices switched to electronic records, meaning the primary care records further back in time are less complete. We therefore have less information on participants earlier in life compared to later life, which may limit the use of cohort to study early or midlife risk factors for dementia. The introduction of QOF from 2004 onwards changed the way in which GPs were remunerated, and this led to changes in how GPs coded diagnoses and symptoms for certain conditions [57,58]. Dementia was introduced to QOF in 2006/2007, resulting in a sudden increase in dementia primary care codes around this time. Researchers using SAIL-DeC for survival analyses may wish to consider this when selecting their study time window.

## Conclusion

In conclusion, we have applied coding algorithms to primary care, hospital admissions and mortality data to create SAIL-DeC, a national dementia e-cohort, to complement existing cohorts within DPUK. The cohort will enable researchers to conduct a wide range of analyses related to dementia, whilst minimising duplication of effort, time and cost.

## Ethics statement

Ethical approval was not required because the study used only anonymised data. Approval was granted by the Information Governance Review Panel (IGRP, application number: 0697). Composed of government, regulatory and professional agencies, the IGRP oversees and approves applications to use the SAIL databank.

## Supplementary Material

Supplementary Material

## Abbreviations

ADDE	Annual District Death Extract
ALF	Anonymised Linking Field
DPUK	Dementias Platform UK
GP	General practice
ICD	International Classification of Diseases
IGRP	Information Governance Review Panel
LSOA	Lower Super Output Area
NHS	National Health Service
PEDW	Patient Episode Database for Wales
PPV	Positive Predictive Value
QOF	Quality Outcomes Framework
Read V2	Read version 2
RECORD	The REporting of studies Conducted using Observational Routinely-collected health Data statement
SAIL	Secure Anonymised Information Linkage (Databank)
SAIL-DeC	SAIL dementia electronic cohort
SNOMED CT	Systematised Nomenclature of Medicine & Clinical Terms
UK	United Kingdom
WDSD	Welsh Demographic Service Dataset
WIMD	Welsh Index of Multiple Deprivation
WLGP	Welsh Longitudinal General Practice dataset
