# Supplementary material for: The Secure Anonymised Information Linkage databank Dementia e-cohort (SAIL-DeC)
Source: Int J Popul Data Sci. 2020 Feb 25;5(1):1121. doi: 10.23889/ijpds.v5i1.1121 (PMC7473277; doi:10.23889/ijpds.v5i1.1121)
Supplement: Supplementary Material [file ijpds-05-01-1121-s001.zip › Supplementary Appendix 10.html]

Event tables


# Event tables

### *Asthma*

#### *Christian*

#### *January 2019*

## Code selection

We have selected codes based on the UK Biobank algorithm and asthma validation study (unpublished) in conjunction with the WHO ICD 10 browser (apps.who.int/classifications/icd10/browse/2010/en) and the NHS Read Code Browser (https://isd.digital.nhs.uk/trud3/user/guest/group/0/home). We have deliberately included codes with obvious `misspelling’ (for example having a dot where none should be) or ICD 10 codes ending with ‘X’.

All codes that were selected for classification and the total number of people with at least one of the codes are displayed in the following tables. Please be aware that frequency counts of Read V2 codes in the table do not reflect the hierarchical nature of Read V2 coding (for example, counts of E01.. do not include E011.).

### Read V2 codes:

| code | desc | total\_n |
| --- | --- | --- |
| 173A. | Exercise induced asthma | 2515 |
| 173d. | Work aggravated asthma | <5 |
| 1780. | Aspirin induced asthma | 103 |
| 1781. | Asthma trigger - pollen | 4201 |
| 1782. | Asthma trigger - tobacco smoke | 1691 |
| 1783. | Asthma trigger - warm air | 2279 |
| 1784. | Asthma trigger - emotion | 1934 |
| 1785. | Asthma trigger - damp | 2362 |
| 1786. | Asthma trigger - animals | 1639 |
| 1787. | Asthma trigger - seasonal | 2497 |
| 1788. | Asthma trigger - cold air | 5090 |
| 1789. | Asthma trigger - respiratory infection | 8142 |
| 178A. | Asthma trigger - airborne dust | 3335 |
| 178B. | Asthma trigger - exercise | 4310 |
| 1O2.. | Asthma confirmed | 1645 |
| 388t. | Royal College of Physicians asthma assessment | 6695 |
| 388t0 | Royal College of Physicians asthma assessment three questions score | 2526 |
| 38DL. | Asthma control test | 3070 |
| 38DT. | Asthma control questionnaire | 233 |
| 661M1 | Asthma self-management plan agreed | 6683 |
| 661N1 | Asthma self-management plan review | 667 |
| 663.. | Respiratory disease monitoring | 76497 |
| 663d. | Emergency asthma admission since last appointment | 2011 |
| 663e. | Asthma restricts exercise | 9165 |
| 663e0 | Asthma sometimes restricts exercise | 18381 |
| 663e1 | Asthma severely restricts exercise | 2971 |
| 663f. | Asthma never restricts exercise | 16140 |
| 663j. | Asthma - currently active | 7963 |
| 663m. | Asthma accident and emergency attendance since last visit | 3817 |
| 663n. | Asthma treatment compliance satisfactory | 31705 |
| 663N. | Asthma disturbing sleep | 27938 |
| 663N0 | Asthma causing night waking | 7342 |
| 663N1 | Asthma disturbs sleep weekly | 5566 |
| 663N2 | Asthma disturbs sleep frequently | 4051 |
| 663O. | Asthma not disturbing sleep | 85850 |
| 663O0 | Asthma never disturbs sleep | 18706 |
| 663P. | Asthma limiting activities | 40970 |
| 663P0 | Asthma limits activities 1 to 2 times per month | 1365 |
| 663P1 | Asthma limits activities 1 to 2 times per week | 1935 |
| 663P2 | Asthma limits activities most days | 2499 |
| 663q. | Asthma daytime symptoms | 53419 |
| 663r. | Asthma causes night symptoms 1 to 2 times per month | 6939 |
| 663s. | Asthma never causes daytime symptoms | 50140 |
| 663t. | Asthma causes daytime symptoms 1 to 2 times per month | 29132 |
| 663u. | Asthma causes daytime symptoms 1 to 2 times per week | 27084 |
| 663U. | Asthma management plan given | 56834 |
| 663v. | Asthma causes daytime symptoms most days | 29837 |
| 663V. | Asthma severity | 3852 |
| 663V0 | Occasional asthma | 1029 |
| 663V1 | Mild asthma | 2388 |
| 663V2 | Moderate asthma | 2397 |
| 663V3 | Severe asthma | 759 |
| 663w. | Asthma limits walking up hills or stairs | 12825 |
| 663x. | Asthma limits walking on the flat | 2913 |
| 663y. | Number of asthma exacerbations in past year | 14171 |
| 66Y5. | Change in asthma management plan | 850 |
| 66Y9. | Step up change in asthma management plan | 2962 |
| 66YA. | Step down change in asthma management plan | 2166 |
| 66YC. | Absent from work or school due to asthma | 154 |
| 66YJ. | Asthma annual review | 105116 |
| 66YK. | Asthma follow-up | 37503 |
| 66Yp. | Asthma review using Royal College of Physicians three questions | 61890 |
| 66YP. | Asthma night-time symptoms | 6815 |
| 66Yq. | Asthma causes night time symptoms 1 to 2 times per week | 3937 |
| 66YQ. | Asthma monitoring by nurse | 21886 |
| 66Yr. | Asthma causes symptoms most nights | 3064 |
| 66YR. | Asthma monitoring by doctor | 5592 |
| 66Ys. | Asthma never causes night symptoms | 5538 |
| 679J0 | Health education - asthma self management | 537 |
| 679J1 | Health education - structured asthma discussion | 261 |
| 8794. | Asthma control step 1 | 3818 |
| 8795. | Asthma control step 2 | 8265 |
| 8796. | Asthma control step 3 | 8587 |
| 8797. | Asthma control step 4 | 1822 |
| 8798. | Asthma control step 5 | 469 |
| 8B3j. | Asthma medication review | 60341 |
| 8CMA0 | Patient has a written asthma personal action plan | 996 |
| 8CR0. | Asthma clinical management plan | 1070 |
| 8H2P. | Emergency admission, asthma | 2664 |
| 9OJ1. | Attends asthma monitoring | 1686 |
| 9OJA. | Asthma monitoring check done | 31825 |
| H33.. | Asthma | 141927 |
| H330. | Extrinsic (atopic) asthma | 3287 |
| H3300 | Extrinsic asthma without status asthmaticus | 486 |
| H3301 | Extrinsic asthma with status asthmaticus | 295 |
| H330z | Extrinsic asthma NOS | 109 |
| H331. | Intrinsic asthma | 2759 |
| H3310 | Intrinsic asthma without status asthmaticus | 131 |
| H3311 | Intrinsic asthma with status asthmaticus | 42 |
| H331z | Intrinsic asthma NOS | 164 |
| H332. | Mixed asthma | 357 |
| H333. | Acute exacerbation of asthma | 26862 |
| H334. | Brittle asthma | 26 |
| H335. | Chronic asthma with fixed airflow obstruction | 191 |
| H33z. | Asthma unspecified | 1886 |
| H33z0 | Status asthmaticus NOS | 1366 |
| H33z1 | Asthma attack | 5197 |
| H33z2 | Late-onset asthma | 862 |
| H33zz | Asthma NOS | 6473 |
| 663m0 | NA | <5 |

### ICD 9 and 10 codes:

| code | desc | total\_n |
| --- | --- | --- |
| 493 | Asthma | 0 |
| 4930 | Extrinsic asthma | <5 |
| 4931 | Intrinsic asthma | <5 |
| 4939 | Asthma unspecified | 496 |
| J45 | Asthma | <5 |
| J450 | Predominantly allergic asthma | 1694 |
| J451 | Nonallergic asthma | 47 |
| J458 | Mixed asthma | 65 |
| J459 | Asthma unspecified | 126352 |
| J45X | NA | 16 |
| J46 | Status asthmaticus | 23 |
| J460 | NA | <5 |
| J46X | NA | 1513 |

## Descriptives

217504 people had at least one diagnostic code in at least one of the datasets. 127127 people had a code in hospital admissions data, 2467 in mortality data and 179422 in primary care data. The following figure shows the year of the first code that was found for any person classified positive using (a) all codes combined, (b) only codes from hospital admissions data, (c) only codes from the mortality data and (d) only codes from primary care data.
