# Supplementary material for: The Secure Anonymised Information Linkage databank Dementia e-cohort (SAIL-DeC)
Source: Int J Popul Data Sci. 2020 Feb 25;5(1):1121. doi: 10.23889/ijpds.v5i1.1121 (PMC7473277; doi:10.23889/ijpds.v5i1.1121)
Supplement: Supplementary Material [file ijpds-05-01-1121-s001.zip › Supplementary Appendix 11.html]

Event tables


# Event tables

### *Atrial fibrillation*

#### *Christian*

#### *January 2019*

## Code selection

We have selected codes based on QoF Business Rules 24 (https://www.pcc-cic.org.uk/article/qof-business-rules-v240 ) in conjunction with the WHO ICD 10 browser (apps.who.int/classifications/icd10/browse/2010/en) and the NHS Read Code Browser (https://isd.digital.nhs.uk/trud3/user/guest/group/0/home). We have deliberately included codes with obvious `misspelling’ (for example having a dot where none should be) or ICD 10 codes ending with ‘X’.

All codes that were selected for classification and the total number of people with at least one of the codes are displayed in the following tables. Please be aware that frequency counts of Read V2 codes in the table do not reflect the hierarchical nature of Read V2 coding (for example, counts of E01.. do not include E011.).

### Read V2 codes:

| code | desc | total\_n |
| --- | --- | --- |
| G573. | Atrial fibrillation and flutter | 28994 |
| G5730 | Atrial fibrillation | 105710 |
| G5731 | Atrial flutter | 5993 |
| G5732 | Paroxysmal atrial fibrillation | 20815 |
| G5733 | Non-rheumatic atrial fibrillation | 95 |
| G5734 | Permanent atrial fibrillation | 182 |
| G5735 | Persistent atrial fibrillation | 231 |
| G5736 | Paroxysmal atrial flutter | 103 |
| G5737 | Chronic atrial fibrillation | 10 |
| G5738 | Typical atrial flutter | <5 |
| G5739 | Atypical atrial flutter | 6 |
| G573z | Atrial fibrillation and flutter NOS | 854 |

### ICD 9 and 10 codes:

| code | desc | total\_n |
| --- | --- | --- |
| 4273 | Atrial fibrillation and flutter | 1420 |
| I48 | Atrial fibrillation and flutter | 12840 |
| I48- | NA | <5 |
| I48# | NA | <5 |
| I48. | NA | 5 |
| I480 | Paroxysmal atrial fibrillation | 8013 |
| I481 | Persistent atrial fibrillation | 498 |
| I482 | Chronic atrial fibrillation | 314 |
| I483 | Typical atrial flutter | 66 |
| I484 | Atypical atrial flutter | 69 |
| I489 | Atrial fibrillation and atrial flutter unspecified | 50489 |
| I48X | NA | 174513 |

## Descriptives

222721 people had at least one diagnostic code in at least one of the datasets. 198197 people had a code in hospital admissions data, 18483 in mortality data and 134863 in primary care data. The following figure shows the year of the first code that was found for any person classified positive using (a) all codes combined, (b) only codes from hospital admissions data, (c) only codes from the mortality data and (d) only codes from primary care data.
