# Supplementary material for: The Secure Anonymised Information Linkage databank Dementia e-cohort (SAIL-DeC)
Source: Int J Popul Data Sci. 2020 Feb 25;5(1):1121. doi: 10.23889/ijpds.v5i1.1121 (PMC7473277; doi:10.23889/ijpds.v5i1.1121)
Supplement: Supplementary Material [file ijpds-05-01-1121-s001.zip › Supplementary Appendix 12.html]

Event tables


# Event tables

### *Cancer*

#### *Christian*

#### *January 2019*

## Code selection

We have selected codes based on QOF Business rules v24 (https://www.pcc-cic.org.uk/article/qof-business-rules-v240) in conjunction with the WHO ICD 10 browser (apps.who.int/classifications/icd10/browse/2010/en) and the NHS Read Code Browser (https://isd.digital.nhs.uk/trud3/user/guest/group/0/home). We have deliberately included codes with obvious `misspelling’ (for example having a dot where none should be) or ICD 10 codes ending with ‘X’.

All codes that were selected for classification and the total number of people with at least one of the codes are displayed in the following tables. Please be aware that frequency counts of Read V2 codes in the table do not reflect the hierarchical nature of Read V2 coding (for example, counts of E01.. do not include E011.).

Researchers with specific interest in cancers are advised that data in the Dementia Cohort can be linked to a more comprehensive Welsh Cancer Intelligence and surveillance data set.

### Read V2 codes:

| code | desc | total\_n |
| --- | --- | --- |
| B0… | Malignant neoplasm of lip, oral cavity and pharynx | 355 |
| B00.. | Malignant neoplasm of lip | 244 |
| B000. | Malignant neoplasm of upper lip, vermilion border | 0 |
| B0000 | Malignant neoplasm of upper lip, external | 0 |
| B0001 | Malignant neoplasm of upper lip, lipstick area | 0 |
| B000z | Malignant neoplasm of upper lip, vermilion border NOS | <5 |
| B001. | Malignant neoplasm of lower lip, vermilion border | 5 |
| B0010 | Malignant neoplasm of lower lip, external | 9 |
| B0011 | Malignant neoplasm of lower lip, lipstick area | 0 |
| B001z | Malignant neoplasm of lower lip, vermilion border NOS | <5 |
| B002. | Malignant neoplasm of upper lip, inner aspect | 0 |
| B0020 | Malignant neoplasm of upper lip, buccal aspect | 0 |
| B0021 | Malignant neoplasm of upper lip, frenulum | <5 |
| B0022 | Malignant neoplasm of upper lip, mucosa | <5 |
| B0023 | Malignant neoplasm of upper lip, oral aspect | 0 |
| B002z | Malignant neoplasm of upper lip, inner aspect NOS | 0 |
| B003. | Malignant neoplasm of lower lip, inner aspect | <5 |
| B0030 | Malignant neoplasm of lower lip, buccal aspect | 0 |
| B0031 | Malignant neoplasm of lower lip, frenulum | 0 |
| B0032 | Malignant neoplasm of lower lip, mucosa | 5 |
| B0033 | Malignant neoplasm of lower lip, oral aspect | <5 |
| B003z | Malignant neoplasm of lower lip, inner aspect NOS | <5 |
| B004. | Malignant neoplasm of lip unspecified, inner aspect | <5 |
| B0040 | Malignant neoplasm of lip unspecified, buccal aspect | 0 |
| B0041 | Malignant neoplasm of lip unspecified, frenulum | 0 |
| B0042 | Malignant neoplasm of lip unspecified, mucosa | <5 |
| B0043 | Malignant neoplasm of lip, oral aspect | <5 |
| B004z | Malignant neoplasm of lip, inner aspect NOS | 0 |
| B005. | Malignant neoplasm of commissure of lip | <5 |
| B006. | Malignant neoplasm of overlapping lesion of lip | <5 |
| B007. | Malignant neoplasm of lip, unspecified | 8 |
| B00y. | Malignant neoplasm of other sites of lip | 0 |
| B00z. | Malignant neoplasm of vermilion border of lip unspecified | 0 |
| B00z0 | Malignant neoplasm of lip, unspecified, external | 0 |
| B00z1 | Malignant neoplasm of lip, unspecified, lipstick area | 0 |
| B00zz | Malignant neoplasm of lip, vermilion border NOS | <5 |
| B01.. | Malignant neoplasm of tongue | 682 |
| B010. | Malignant neoplasm of base of tongue | 151 |
| B0100 | Malignant neoplasm of base of tongue dorsal surface | <5 |
| B010z | Malignant neoplasm of fixed part of tongue NOS | <5 |
| B011. | Malignant neoplasm of dorsal surface of tongue | <5 |
| B0110 | Malignant neoplasm of anterior 2/3 of tongue dorsal surface | 0 |
| B0111 | Malignant neoplasm of midline of tongue | <5 |
| B011z | Malignant neoplasm of dorsum of tongue NOS | <5 |
| B012. | Malignant neoplasm of tongue, tip and lateral border | 24 |
| B013. | Malignant neoplasm of ventral surface of tongue | 11 |
| B0130 | Malignant neoplasm of anterior 2/3 of tongue ventral surface | <5 |
| B0131 | Malignant neoplasm of frenulum linguae | 0 |
| B013z | Malignant neoplasm of ventral tongue surface NOS | <5 |
| B014. | Malignant neoplasm of anterior 2/3 of tongue unspecified | 11 |
| B015. | Malignant neoplasm of tongue, junctional zone | 0 |
| B016. | Malignant neoplasm of lingual tonsil | 36 |
| B017. | Malignant overlapping lesion of tongue | <5 |
| B01y. | Malignant neoplasm of other sites of tongue | <5 |
| B01z. | Malignant neoplasm of tongue NOS | 63 |
| B02.. | Malignant neoplasm of major salivary glands | 70 |
| B020. | Malignant neoplasm of parotid gland | 324 |
| B021. | Malignant neoplasm of submandibular gland | 46 |
| B022. | Malignant neoplasm of sublingual gland | <5 |
| B023. | Malignant neoplasm, overlapping lesion of major salivary glands | 0 |
| B02y. | Malignant neoplasm of other major salivary glands | <5 |
| B02z. | Malignant neoplasm of major salivary gland NOS | <5 |
| B03.. | Malignant neoplasm of gum | 59 |
| B030. | Malignant neoplasm of upper gum | 13 |
| B031. | Malignant neoplasm of lower gum | 20 |
| B03y. | Malignant neoplasm of other sites of gum | 0 |
| B03z. | Malignant neoplasm of gum NOS | <5 |
| B04.. | Malignant neoplasm of floor of mouth | 181 |
| B040. | Malignant neoplasm of anterior portion of floor of mouth | 23 |
| B041. | Malignant neoplasm of lateral portion of floor of mouth | 12 |
| B042. | Malignant neoplasm, overlapping lesion of floor of mouth | <5 |
| B04y. | Malignant neoplasm of other sites of floor of mouth | <5 |
| B04z. | Malignant neoplasm of floor of mouth NOS | 29 |
| B05.. | Malignant neoplasm of other and unspecified parts of mouth | 59 |
| B050. | Malignant neoplasm of cheek mucosa | 58 |
| B051. | Malignant neoplasm of vestibule of mouth | <5 |
| B0510 | Malignant neoplasm of upper buccal sulcus | <5 |
| B0511 | Malignant neoplasm of lower buccal sulcus | <5 |
| B0512 | Malignant neoplasm of upper labial sulcus | <5 |
| B0513 | Malignant neoplasm of lower labial sulcus | <5 |
| B051z | Malignant neoplasm of vestibule of mouth NOS | 0 |
| B052. | Malignant neoplasm of hard palate | 29 |
| B053. | Malignant neoplasm of soft palate | 73 |
| B054. | Malignant neoplasm of uvula | 23 |
| B055. | Malignant neoplasm of palate unspecified | 9 |
| B0550 | Malignant neoplasm of junction of hard and soft palate | 6 |
| B0551 | Malignant neoplasm of roof of mouth | <5 |
| B055z | Malignant neoplasm of palate NOS | 8 |
| B056. | Malignant neoplasm of retromolar area | 37 |
| B057. | Overlapping lesion of other and unspecified parts of mouth | 30 |
| B05y. | Malignant neoplasm of other specified mouth parts | 6 |
| B05z. | Malignant neoplasm of mouth NOS | 47 |
| B05z0 | Kaposi’s sarcoma of palate | 0 |
| B06.. | Malignant neoplasm of oropharynx | 229 |
| B060. | Malignant neoplasm of tonsil | 455 |
| B0600 | Malignant neoplasm of faucial tonsil | <5 |
| B0601 | Malignant neoplasm of palatine tonsil | <5 |
| B0602 | Malignant neoplasm of overlapping lesion of tonsil | <5 |
| B060z | Malignant neoplasm tonsil NOS | 95 |
| B061. | Malignant neoplasm of tonsillar fossa | 44 |
| B062. | Malignant neoplasm of tonsillar pillar | 7 |
| B0620 | Malignant neoplasm of faucial pillar | <5 |
| B0621 | Malignant neoplasm of glossopalatine fold | <5 |
| B0622 | Malignant neoplasm of palatoglossal arch | 0 |
| B0623 | Malignant neoplasm of palatopharyngeal arch | <5 |
| B062z | Malignant neoplasm of tonsillar fossa NOS | <5 |
| B063. | Malignant neoplasm of vallecula | 19 |
| B064. | Malignant neoplasm of anterior epiglottis | 24 |
| B0640 | Malignant neoplasm of epiglottis, free border | 9 |
| B0641 | Malignant neoplasm of glossoepiglottic fold | <5 |
| B064z | Malignant neoplasm of anterior epiglottis NOS | 9 |
| B065. | Malignant neoplasm of junctional region of epiglottis | <5 |
| B066. | Malignant neoplasm of lateral wall of oropharynx | 7 |
| B067. | Malignant neoplasm of posterior wall of oropharynx | 6 |
| B06y. | Malignant neoplasm of oropharynx, other specified sites | 10 |
| B06y0 | Malignant neoplasm of branchial cleft | 0 |
| B06yz | Malignant neoplasm of other specified site of oropharynx NOS | <5 |
| B06z. | Malignant neoplasm of oropharynx NOS | 20 |
| B07.. | Malignant neoplasm of nasopharynx | 106 |
| B070. | Malignant neoplasm of roof of nasopharynx | 0 |
| B071. | Malignant neoplasm of posterior wall of nasopharynx | <5 |
| B0710 | Malignant neoplasm of adenoid | 7 |
| B0711 | Malignant neoplasm of pharyngeal tonsil | 19 |
| B071z | Malignant neoplasm of posterior wall of nasopharynx NOS | <5 |
| B072. | Malignant neoplasm of lateral wall of nasopharynx | <5 |
| B0720 | Malignant neoplasm of pharyngeal recess | 26 |
| B0721 | Malignant neoplasm of opening of auditory tube | <5 |
| B072z | Malignant neoplasm of lateral wall of nasopharynx NOS | <5 |
| B073. | Malignant neoplasm of anterior wall of nasopharynx | 0 |
| B0730 | Malignant neoplasm of floor of nasopharynx | 0 |
| B0731 | Malignant neoplasm of nasopharyngeal soft palate surface | <5 |
| B0732 | Malignant neoplasm of posterior margin of nasal septum and choanae | <5 |
| B073z | Malignant neoplasm of anterior wall of nasopharynx NOS | 0 |
| B074. | Malignant neoplasm, overlapping lesion of nasopharynx | <5 |
| B07y. | Malignant neoplasm of other specified site of nasopharynx | <5 |
| B07z. | Malignant neoplasm of nasopharynx NOS | 12 |
| B08.. | Malignant neoplasm of hypopharynx | 89 |
| B080. | Malignant neoplasm of postcricoid region | 18 |
| B081. | Malignant neoplasm of pyriform sinus | 68 |
| B082. | Malignant neoplasm of aryepiglottic fold, hypopharyngeal aspect | 8 |
| B083. | Malignant neoplasm of posterior pharynx | 10 |
| B084. | Malignant neoplasm, overlapping lesion of hypopharynx | <5 |
| B08y. | Malignant neoplasm of other specified hypopharyngeal site | 8 |
| B08z. | Malignant neoplasm of hypopharynx NOS | 15 |
| B0z.. | Malignant neoplasm of other and ill-defined sites within the lip, oral cavity and pharynx | 7 |
| B0z0. | Malignant neoplasm of pharynx unspecified | 66 |
| B0z1. | Malignant neoplasm of Waldeyer’s ring | 0 |
| B0z2. | Malignant neoplasm of laryngopharynx | 101 |
| B0zy. | Malignant neoplasm of other sites of lip, oral cavity and pharynx | 11 |
| B0zz. | Malignant neoplasm of lip, oral cavity and pharynx NOS | 144 |
| B10.. | Malignant neoplasm of oesophagus | 3680 |
| B100. | Malignant neoplasm of cervical oesophagus | 12 |
| B101. | Malignant neoplasm of thoracic oesophagus | 19 |
| B102. | Malignant neoplasm of abdominal oesophagus | 23 |
| B103. | Malignant neoplasm of upper third of oesophagus | 30 |
| B104. | Malignant neoplasm of middle third of oesophagus | 55 |
| B105. | Malignant neoplasm of lower third of oesophagus | 314 |
| B106. | Malignant neoplasm, overlapping lesion of oesophagus | <5 |
| B107. | Siewert type I adenocarcinoma | 8 |
| B10y. | Malignant neoplasm of other specified part of oesophagus | 18 |
| B10z. | Malignant neoplasm of oesophagus NOS | 2874 |
| B11.. | Malignant neoplasm of stomach | 3306 |
| B110. | Malignant neoplasm of cardia of stomach | 148 |
| B1100 | Malignant neoplasm of cardiac orifice of stomach | <5 |
| B1101 | Malignant neoplasm of cardio-oesophageal junction of stomach | 159 |
| B110z | Malignant neoplasm of cardia of stomach NOS | 19 |
| B111. | Malignant neoplasm of pylorus of stomach | 48 |
| B1110 | Malignant neoplasm of prepylorus of stomach | 13 |
| B1111 | Malignant neoplasm of pyloric canal of stomach | 24 |
| B111z | Malignant neoplasm of pylorus of stomach NOS | 5 |
| B112. | Malignant neoplasm of pyloric antrum of stomach | 34 |
| B113. | Malignant neoplasm of fundus of stomach | 27 |
| B114. | Malignant neoplasm of body of stomach | 64 |
| B115. | Malignant neoplasm of lesser curve of stomach unspecified | 48 |
| B116. | Malignant neoplasm of greater curve of stomach unspecified | 17 |
| B117. | Malignant neoplasm, overlapping lesion of stomach | 0 |
| B118. | Siewert type II adenocarcinoma | 18 |
| B119. | Siewert type III adenocarcinoma | 15 |
| B11y. | Malignant neoplasm of other specified site of stomach | 14 |
| B11y0 | Malignant neoplasm of anterior wall of stomach NEC | <5 |
| B11y1 | Malignant neoplasm of posterior wall of stomach NEC | <5 |
| B11yz | Malignant neoplasm of other specified site of stomach NOS | <5 |
| B11z. | Malignant neoplasm of stomach NOS | 635 |
| B12.. | Malignant neoplasm of small intestine and duodenum | 1101 |
| B120. | Malignant neoplasm of duodenum | 164 |
| B121. | Malignant neoplasm of jejunum | 27 |
| B122. | Malignant neoplasm of ileum | 37 |
| B123. | Malignant neoplasm of Meckel’s diverticulum | <5 |
| B124. | Malignant neoplasm, overlapping lesion of small intestine | <5 |
| B12y. | Malignant neoplasm of other specified site of small intestine | <5 |
| B12z. | Malignant neoplasm of small intestine NOS | 53 |
| B13.. | Malignant neoplasm of colon | 8970 |
| B130. | Malignant neoplasm of hepatic flexure of colon | 218 |
| B131. | Malignant neoplasm of transverse colon | 370 |
| B132. | Malignant neoplasm of descending colon | 279 |
| B133. | Malignant neoplasm of sigmoid colon | 2842 |
| B134. | Malignant neoplasm of caecum | 2735 |
| B135. | Malignant neoplasm of appendix | 117 |
| B136. | Malignant neoplasm of ascending colon | 547 |
| B137. | Malignant neoplasm of splenic flexure of colon | 197 |
| B138. | Malignant neoplasm, overlapping lesion of colon | <5 |
| B139. | Hereditary nonpolyposis colon cancer | 6 |
| B13y. | Malignant neoplasm of other specified sites of colon | 27 |
| B13z. | Malignant neoplasm of colon NOS | 3619 |
| B14.. | Malignant neoplasm of rectum, rectosigmoid junction and anus | 370 |
| B140. | Malignant neoplasm of rectosigmoid junction | 512 |
| B141. | Malignant neoplasm of rectum | 9032 |
| B142. | Malignant neoplasm of anal canal | 409 |
| B1420 | Malignant neoplasm of cloacogenic zone | <5 |
| B143. | Malignant neoplasm of anus unspecified | 133 |
| B14y. | Malignant neoplasm of other sites of rectum, rectosigmoid junction and anus | 12 |
| B14z. | Malignant neoplasm of rectum, rectosigmoid junction and anus NOS | 22 |
| B15.. | Malignant neoplasm of liver and intrahepatic bile ducts | 90 |
| B150. | Primary malignant neoplasm of liver | 184 |
| B1500 | Primary carcinoma of liver | 71 |
| B1501 | Hepatoblastoma of liver | 0 |
| B1502 | Primary angiosarcoma of liver | <5 |
| B1503 | Hepatocellular carcinoma | 717 |
| B150z | Primary malignant neoplasm of liver NOS | 19 |
| B151. | Malignant neoplasm of intrahepatic bile ducts | 52 |
| B1510 | Malignant neoplasm of interlobular bile ducts | <5 |
| B1511 | Malignant neoplasm of interlobular biliary canals | <5 |
| B1512 | Malignant neoplasm of intrahepatic biliary passages | <5 |
| B1513 | Malignant neoplasm of intrahepatic canaliculi | <5 |
| B1514 | Malignant neoplasm of intrahepatic gall duct | 7 |
| B151z | Malignant neoplasm of intrahepatic bile ducts NOS | 11 |
| B152. | Malignant neoplasm of liver unspecified | 360 |
| B153. | Secondary malignant neoplasm of liver | 227 |
| B15z. | Malignant neoplasm of liver and intrahepatic bile ducts NOS | 24 |
| B16.. | Malignant neoplasm of gallbladder and extrahepatic bile ducts | 35 |
| B160. | Malignant neoplasm of gallbladder | 422 |
| B161. | Malignant neoplasm of extrahepatic bile ducts | 48 |
| B1610 | Malignant neoplasm of cystic duct | <5 |
| B1611 | Malignant neoplasm of hepatic duct | 13 |
| B1612 | Malignant neoplasm of common bile duct | 153 |
| B1613 | Malignant neoplasm of sphincter of Oddi | <5 |
| B161z | Malignant neoplasm of extrahepatic bile ducts NOS | 7 |
| B162. | Malignant neoplasm of ampulla of Vater | 111 |
| B163. | Malignant neoplasm, overlapping lesion of biliary tract | 5 |
| B16y. | Malignant neoplasm of other gallbladder and extrahepatic bile ducts | <5 |
| B16z. | Malignant neoplasm of gallbladder and extrahepatic bile ducts NOS | 14 |
| B17.. | Malignant neoplasm of pancreas | 3084 |
| B170. | Malignant neoplasm of head of pancreas | 611 |
| B171. | Malignant neoplasm of body of pancreas | 65 |
| B172. | Malignant neoplasm of tail of pancreas | 88 |
| B173. | Malignant neoplasm of pancreatic duct | 67 |
| B174. | Malignant neoplasm of Islets of Langerhans | 0 |
| B175. | Malignant neoplasm, overlapping lesion of pancreas | 0 |
| B176. | Somatostatinoma of pancreas | <5 |
| B17y. | Malignant neoplasm of other specified sites of pancreas | <5 |
| B17y0 | Malignant neoplasm of ectopic pancreatic tissue | <5 |
| B17yz | Malignant neoplasm of specified site of pancreas NOS | 5 |
| B17z. | Malignant neoplasm of pancreas NOS | 458 |
| B21.. | Malignant neoplasm of larynx | 1069 |
| B210. | Malignant neoplasm of glottis | 223 |
| B211. | Malignant neoplasm of supraglottis | 175 |
| B212. | Malignant neoplasm of subglottis | 19 |
| B213. | Malignant neoplasm of laryngeal cartilage | 47 |
| B2130 | Malignant neoplasm of arytenoid cartilage | <5 |
| B2131 | Malignant neoplasm of cricoid cartilage | 10 |
| B2132 | Malignant neoplasm of cuneiform cartilage | <5 |
| B2133 | Malignant neoplasm of thyroid cartilage | 19 |
| B213z | Malignant neoplasm of laryngeal cartilage NOS | 21 |
| B214. | Malignant neoplasm, overlapping lesion of larynx | <5 |
| B215. | Malignant neoplasm of epiglottis NOS | 53 |
| B21y. | Malignant neoplasm of larynx, other specified site | 30 |
| B21z. | Malignant neoplasm of larynx NOS | 239 |
| B22.. | Malignant neoplasm of trachea, bronchus and lung | 3190 |
| B220. | Malignant neoplasm of trachea | 34 |
| B2200 | Malignant neoplasm of cartilage of trachea | 0 |
| B2201 | Malignant neoplasm of mucosa of trachea | 0 |
| B220z | Malignant neoplasm of trachea NOS | <5 |
| B221. | Malignant neoplasm of main bronchus | 1040 |
| B2210 | Malignant neoplasm of carina of bronchus | 52 |
| B2211 | Malignant neoplasm of hilus of lung | 170 |
| B221z | Malignant neoplasm of main bronchus NOS | 202 |
| B222. | Malignant neoplasm of upper lobe, bronchus or lung | 937 |
| B2220 | Malignant neoplasm of upper lobe bronchus | 247 |
| B2221 | Malignant neoplasm of upper lobe of lung | 1190 |
| B222z | Malignant neoplasm of upper lobe, bronchus or lung NOS | 143 |
| B223. | Malignant neoplasm of middle lobe, bronchus or lung | 130 |
| B2230 | Malignant neoplasm of middle lobe bronchus | 27 |
| B2231 | Malignant neoplasm of middle lobe of lung | 129 |
| B223z | Malignant neoplasm of middle lobe, bronchus or lung NOS | 25 |
| B224. | Malignant neoplasm of lower lobe, bronchus or lung | 463 |
| B2240 | Malignant neoplasm of lower lobe bronchus | 110 |
| B2241 | Malignant neoplasm of lower lobe of lung | 648 |
| B224z | Malignant neoplasm of lower lobe, bronchus or lung NOS | 87 |
| B225. | Malignant neoplasm of overlapping lesion of bronchus and lung | 31 |
| B226. | Mesothelioma | 792 |
| B22y. | Malignant neoplasm of other sites of bronchus or lung | 190 |
| B22z. | Malignant neoplasm of bronchus or lung NOS | 15952 |
| B32.. | Malignant melanoma of skin | 6497 |
| B320. | Malignant melanoma of lip | 10 |
| B321. | Malignant melanoma of eyelid including canthus | 20 |
| B322. | Malignant melanoma of ear and external auricular canal | 31 |
| B3220 | Malignant melanoma of auricle (ear) | 17 |
| B3221 | Malignant melanoma of external auditory meatus | 0 |
| B322z | Malignant melanoma of ear and external auricular canal NOS | <5 |
| B323. | Malignant melanoma of other and unspecified parts of face | 36 |
| B3230 | Malignant melanoma of external surface of cheek | 75 |
| B3231 | Malignant melanoma of chin | 6 |
| B3232 | Malignant melanoma of eyebrow | <5 |
| B3233 | Malignant melanoma of forehead | 29 |
| B3234 | Malignant melanoma of external surface of nose | 23 |
| B3235 | Malignant melanoma of temple | 15 |
| B323z | Malignant melanoma of face NOS | 43 |
| B324. | Malignant melanoma of scalp and neck | 57 |
| B3240 | Malignant melanoma of scalp | 66 |
| B3241 | Malignant melanoma of neck | 64 |
| B324z | Malignant melanoma of scalp and neck NOS | <5 |
| B325. | Malignant melanoma of trunk (excluding scrotum) | 87 |
| B3250 | Malignant melanoma of axilla | 25 |
| B3251 | Malignant melanoma of breast | 174 |
| B3252 | Malignant melanoma of buttock | 8 |
| B3253 | Malignant melanoma of groin | 31 |
| B3254 | Malignant melanoma of perianal skin | 0 |
| B3255 | Malignant melanoma of perineum | <5 |
| B3256 | Malignant melanoma of umbilicus | <5 |
| B3257 | Malignant melanoma of back | 261 |
| B3258 | Malignant melanoma of chest wall | 65 |
| B325z | Malignant melanoma of trunk, excluding scrotum, NOS | 25 |
| B326. | Malignant melanoma of upper limb and shoulder | 83 |
| B3260 | Malignant melanoma of shoulder | 79 |
| B3261 | Malignant melanoma of upper arm | 133 |
| B3262 | Malignant melanoma of fore-arm | 122 |
| B3263 | Malignant melanoma of hand | 10 |
| B3264 | Malignant melanoma of finger | 10 |
| B3265 | Malignant melanoma of thumb | 6 |
| B326z | Malignant melanoma of upper limb or shoulder NOS | <5 |
| B327. | Malignant melanoma of lower limb and hip | 145 |
| B3270 | Malignant melanoma of hip | <5 |
| B3271 | Malignant melanoma of thigh | 108 |
| B3272 | Malignant melanoma of knee | 32 |
| B3273 | Malignant melanoma of popliteal fossa area | <5 |
| B3274 | Malignant melanoma of lower leg | 320 |
| B3275 | Malignant melanoma of ankle | 26 |
| B3276 | Malignant melanoma of heel | 9 |
| B3277 | Malignant melanoma of foot | 46 |
| B3278 | Malignant melanoma of toe | 16 |
| B3279 | Malignant melanoma of great toe | 12 |
| B327z | Malignant melanoma of lower limb or hip NOS | 9 |
| B328. | Malignant melanoma stage IA | 9 |
| B329. | Malignant melanoma stage IB | 11 |
| B32A. | Malignant melanoma stage IIA | 5 |
| B32B. | Malignant melanoma stage IIB | 9 |
| B32C. | Malignant melanoma stage IIC | <5 |
| B32D. | Malignant melanoma stage IIIA | <5 |
| B32E. | Malignant melanoma stage IIIB | <5 |
| B32F. | Malignant melanoma stage IIIC | <5 |
| B32G. | Malignant melanoma stage IV M1a | <5 |
| B32H. | Malignant melanoma stage IV M1b | <5 |
| B32J. | Malignant melanoma stage IV M1c | 0 |
| B32y. | Malignant melanoma of other specified skin site | 47 |
| B32y0 | Overlapping malignant melanoma of skin | 0 |
| B32z. | Malignant melanoma of skin NOS | 183 |
| B34.. | Malignant neoplasm of female breast | 34495 |
| B340. | Malignant neoplasm of nipple and areola of female breast | 130 |
| B3400 | Malignant neoplasm of nipple of female breast | 40 |
| B3401 | Malignant neoplasm of areola of female breast | 10 |
| B340z | Malignant neoplasm of nipple or areola of female breast NOS | <5 |
| B341. | Malignant neoplasm of central part of female breast | 93 |
| B342. | Malignant neoplasm of upper-inner quadrant of female breast | 104 |
| B343. | Malignant neoplasm of lower-inner quadrant of female breast | 54 |
| B344. | Malignant neoplasm of upper-outer quadrant of female breast | 462 |
| B345. | Malignant neoplasm of lower-outer quadrant of female breast | 75 |
| B346. | Malignant neoplasm of axillary tail of female breast | 42 |
| B347. | Malignant neoplasm, overlapping lesion of breast | 6 |
| B34y. | Malignant neoplasm of other site of female breast | 49 |
| B34y0 | Malignant neoplasm of ectopic site of female breast | 0 |
| B34yz | Malignant neoplasm of other site of female breast NOS | 20 |
| B34z. | Malignant neoplasm of female breast NOS | 3443 |
| B35.. | Malignant neoplasm of male breast | 177 |
| B350. | Malignant neoplasm of nipple and areola of male breast | <5 |
| B3500 | Malignant neoplasm of nipple of male breast | <5 |
| B3501 | Malignant neoplasm of areola of male breast | <5 |
| B350z | Malignant neoplasm of nipple or areola of male breast NOS | <5 |
| B35z. | Malignant neoplasm of other site of male breast | 6 |
| B35z0 | Malignant neoplasm of ectopic site of male breast | 0 |
| B35zz | Malignant neoplasm of male breast NOS | 19 |
| B41.. | Malignant neoplasm of cervix uteri | 2021 |
| B410. | Malignant neoplasm of endocervix | 110 |
| B4100 | Malignant neoplasm of endocervical canal | 19 |
| B4101 | Malignant neoplasm of endocervical gland | <5 |
| B410z | Malignant neoplasm of endocervix NOS | 29 |
| B411. | Malignant neoplasm of exocervix | 18 |
| B412. | Malignant neoplasm, overlapping lesion of cervix uteri | <5 |
| B41y. | Malignant neoplasm of other site of cervix | 39 |
| B41y0 | Malignant neoplasm of cervical stump | <5 |
| B41y1 | Malignant neoplasm of squamocolumnar junction of cervix | 9 |
| B41yz | Malignant neoplasm of other site of cervix NOS | 17 |
| B41z. | Malignant neoplasm of cervix uteri NOS | 424 |
| B43.. | Malignant neoplasm of body of uterus | 1266 |
| B430. | Malignant neoplasm of corpus uteri, excluding isthmus | 32 |
| B4300 | Malignant neoplasm of cornu of corpus uteri | <5 |
| B4301 | Malignant neoplasm of fundus of corpus uteri | <5 |
| B4302 | Malignant neoplasm of endometrium of corpus uteri | 2990 |
| B4303 | Malignant neoplasm of myometrium of corpus uteri | 5 |
| B430z | Malignant neoplasm of corpus uteri NOS | 43 |
| B431. | Malignant neoplasm of isthmus of uterine body | <5 |
| B4310 | Malignant neoplasm of lower uterine segment | <5 |
| B431z | Malignant neoplasm of isthmus of uterine body NOS | <5 |
| B432. | Malignant neoplasm of overlapping lesion of corpus uteri | 11 |
| B43y. | Malignant neoplasm of other site of uterine body | 22 |
| B43z. | Malignant neoplasm of body of uterus NOS | 148 |
| B440. | Malignant neoplasm of ovary | 4031 |
| B46.. | Malignant neoplasm of prostate | 31853 |
| B47.. | Malignant neoplasm of testis | 243 |
| B470. | Malignant neoplasm of undescended testis | 0 |
| B4700 | Malignant neoplasm of ectopic testis | 0 |
| B4701 | Malignant neoplasm of retained testis | 0 |
| B4702 | Seminoma of undescended testis | 22 |
| B4703 | Teratoma of undescended testis | <5 |
| B470z | Malignant neoplasm of undescended testis NOS | 0 |
| B471. | Malignant neoplasm of descended testis | <5 |
| B4710 | Seminoma of descended testis | 101 |
| B4711 | Teratoma of descended testis | 34 |
| B471z | Malignant neoplasm of descended testis NOS | 0 |
| B47z. | Malignant neoplasm of testis NOS | 423 |
| B49.. | Malignant neoplasm of urinary bladder | 8782 |
| B490. | Malignant neoplasm of trigone of urinary bladder | 25 |
| B491. | Malignant neoplasm of dome of urinary bladder | 31 |
| B492. | Malignant neoplasm of lateral wall of urinary bladder | 64 |
| B493. | Malignant neoplasm of anterior wall of urinary bladder | 21 |
| B494. | Malignant neoplasm of posterior wall of urinary bladder | 40 |
| B495. | Malignant neoplasm of bladder neck | 40 |
| B496. | Malignant neoplasm of ureteric orifice | 65 |
| B497. | Malignant neoplasm of urachus | <5 |
| B498. | Local recurrence of malignant tumour of urinary bladder | 82 |
| B49y. | Malignant neoplasm of other site of urinary bladder | 34 |
| B49y0 | Malignant neoplasm, overlapping lesion of bladder | 0 |
| B49z. | Malignant neoplasm of urinary bladder NOS | 1666 |
| B4A.. | Malignant neoplasm of kidney and other unspecified urinary organs | 1817 |
| B4A0. | Malignant neoplasm of kidney parenchyma | 941 |
| B4A00 | Hypernephroma | 240 |
| B4A1. | Malignant neoplasm of renal pelvis | 166 |
| B4A10 | Malignant neoplasm of renal calyces | 8 |
| B4A11 | Malignant neoplasm of ureteropelvic junction | <5 |
| B4A1z | Malignant neoplasm of renal pelvis NOS | 36 |
| B4A2. | Malignant neoplasm of ureter | 206 |
| B4A3. | Malignant neoplasm of urethra | 49 |
| B4A4. | Malignant neoplasm of paraurethral glands | <5 |
| B4Ay. | Malignant neoplasm of other urinary organs | 8 |
| B4Ay0 | Malignant neoplasm of overlapping lesion of urinary organs | <5 |
| B4Az. | Malignant neoplasm of kidney or urinary organs NOS | 680 |
| B51.. | Malignant neoplasm of brain | 1184 |
| B510. | Malignant neoplasm of cerebrum (excluding lobes and ventricles) | 18 |
| B5100 | Malignant neoplasm of basal ganglia | <5 |
| B5101 | Malignant neoplasm of cerebral cortex | 5 |
| B5102 | Malignant neoplasm of corpus striatum | 0 |
| B5103 | Malignant neoplasm of globus pallidus | <5 |
| B5104 | Malignant neoplasm of hypothalamus | 0 |
| B5105 | Malignant neoplasm of thalamus | <5 |
| B510z | Malignant neoplasm of cerebrum NOS | 20 |
| B511. | Malignant neoplasm of frontal lobe | 56 |
| B512. | Malignant neoplasm of temporal lobe | 42 |
| B5120 | Malignant neoplasm of hippocampus | <5 |
| B5121 | Malignant neoplasm of uncus | 0 |
| B512z | Malignant neoplasm of temporal lobe NOS | 7 |
| B513. | Malignant neoplasm of parietal lobe | 57 |
| B514. | Malignant neoplasm of occipital lobe | 16 |
| B515. | Malignant neoplasm of cerebral ventricles | 0 |
| B5150 | Malignant neoplasm of choroid plexus | 0 |
| B5151 | Malignant neoplasm of floor of cerebral ventricle | 0 |
| B515z | Malignant neoplasm of cerebral ventricle NOS | <5 |
| B516. | Malignant neoplasm of cerebellum | 42 |
| B517. | Malignant neoplasm of brain stem | 9 |
| B5170 | Malignant neoplasm of cerebral peduncle | <5 |
| B5171 | Malignant neoplasm of medulla oblongata | <5 |
| B5172 | Malignant neoplasm of midbrain | 0 |
| B5173 | Malignant neoplasm of pons | <5 |
| B517z | Malignant neoplasm of brain stem NOS | <5 |
| B51y. | Malignant neoplasm of other parts of brain | 7 |
| B51y0 | Malignant neoplasm of corpus callosum | 6 |
| B51y1 | Malignant neoplasm of tapetum | 0 |
| B51y2 | Malignant neoplasm, overlapping lesion of brain | <5 |
| B51yz | Malignant neoplasm of other part of brain NOS | <5 |
| B51z. | Malignant neoplasm of brain NOS | 104 |
| B53.. | Malignant neoplasm of thyroid gland | 701 |
| B595. | Malignant tumour of unknown origin | 102 |
| B61.. | Hodgkin’s disease | 895 |
| B610. | Hodgkin’s paragranuloma | 0 |
| B6100 | Hodgkin’s paragranuloma of unspecified site | 0 |
| B6101 | Hodgkin’s paragranuloma of lymph nodes of head, face, and neck | 0 |
| B6102 | Hodgkin’s paragranuloma of intrathoracic lymph nodes | 0 |
| B6103 | Hodgkin’s paragranuloma of intra-abdominal lymph nodes | <5 |
| B6104 | Hodgkin’s paragranuloma of lymph nodes of axilla and upper limb | 0 |
| B6105 | Hodgkin’s paragranuloma of lymph nodes of inguinal region and lower limb | 0 |
| B6106 | Hodgkin’s paragranuloma of intrapelvic lymph nodes | 0 |
| B6107 | Hodgkin’s paragranuloma of spleen | 0 |
| B6108 | Hodgkin’s paragranuloma of lymph nodes of multiple sites | 0 |
| B610z | Hodgkin’s paragranuloma NOS | 0 |
| B611. | Hodgkin’s granuloma | <5 |
| B6110 | Hodgkin’s granuloma of unspecified site | 0 |
| B6111 | Hodgkin’s granuloma of lymph nodes of head, face and neck | 0 |
| B6112 | Hodgkin’s granuloma of intrathoracic lymph nodes | 0 |
| B6113 | Hodgkin’s granuloma of intra-abdominal lymph nodes | 0 |
| B6114 | Hodgkin’s granuloma of lymph nodes of axilla and upper limb | 0 |
| B6115 | Hodgkin’s granuloma of lymph nodes of inguinal region and lower limb | 0 |
| B6116 | Hodgkin’s granuloma of intrapelvic lymph nodes | 0 |
| B6117 | Hodgkin’s granuloma of spleen | 0 |
| B6118 | Hodgkin’s granuloma of lymph nodes of multiple sites | 0 |
| B611z | Hodgkin’s granuloma NOS | 0 |
| B612. | Hodgkin’s sarcoma | <5 |
| B6120 | Hodgkin’s sarcoma of unspecified site | 0 |
| B6121 | Hodgkin’s sarcoma of lymph nodes of head, face and neck | 0 |
| B6122 | Hodgkin’s sarcoma of intrathoracic lymph nodes | 0 |
| B6123 | Hodgkin’s sarcoma of intra-abdominal lymph nodes | 0 |
| B6124 | Hodgkin’s sarcoma of lymph nodes of axilla and upper limb | 0 |
| B6125 | Hodgkin’s sarcoma of lymph nodes of inguinal region and lower limb | 0 |
| B6126 | Hodgkin’s sarcoma of intrapelvic lymph nodes | 0 |
| B6127 | Hodgkin’s sarcoma of spleen | 0 |
| B6128 | Hodgkin’s sarcoma of lymph nodes of multiple sites | 0 |
| B612z | Hodgkin’s sarcoma NOS | 0 |
| B613. | Hodgkin’s disease, lymphocytic-histiocytic predominance | 9 |
| B6130 | Hodgkin’s disease, lymphocytic-histiocytic predominance of unspecified site | 0 |
| B6131 | Hodgkin’s disease, lymphocytic-histiocytic predominance of lymph nodes of head, face and neck | <5 |
| B6132 | Hodgkin’s disease, lymphocytic-histiocytic predominance of intrathoracic lymph nodes | <5 |
| B6133 | Hodgkin’s disease, lymphocytic-histiocytic predominance of intra-abdominal lymph nodes | <5 |
| B6134 | Hodgkin’s disease, lymphocytic-histiocytic predominance of lymph nodes of axilla and upper limb | <5 |
| B6135 | Hodgkin’s disease, lymphocytic-histiocytic predominance of lymph nodes of inguinal region and lower limb | 0 |
| B6136 | Hodgkin’s disease, lymphocytic-histiocytic predominance of intrapelvic lymph nodes | 0 |
| B6137 | Hodgkin’s, lymphocytic-histiocytic predominance of spleen | 0 |
| B6138 | Hodgkin’s disease, lymphocytic-histiocytic predominance of lymph nodes of multiple sites | 0 |
| B613z | Hodgkin’s, lymphocytic-histiocytic predominance NOS | <5 |
| B614. | Hodgkin’s disease, nodular sclerosis | 54 |
| B6140 | Hodgkin’s disease, nodular sclerosis of unspecified site | <5 |
| B6141 | Hodgkin’s disease, nodular sclerosis of lymph nodes of head, face and neck | <5 |
| B6142 | Hodgkin’s disease, nodular sclerosis of intrathoracic lymph nodes | 0 |
| B6143 | Hodgkin’s disease, nodular sclerosis of intra-abdominal lymph nodes | <5 |
| B6144 | Hodgkin’s disease, nodular sclerosis of lymph nodes of axilla and upper limb | 0 |
| B6145 | Hodgkin’s disease, nodular sclerosis of lymph nodes of inguinal region and lower limb | 0 |
| B6146 | Hodgkin’s disease, nodular sclerosis of intrapelvic lymph nodes | 0 |
| B6147 | Hodgkin’s disease, nodular sclerosis of spleen | 0 |
| B6148 | Hodgkin’s disease, nodular sclerosis of lymph nodes of multiple sites | <5 |
| B614z | Hodgkin’s disease, nodular sclerosis NOS | <5 |
| B615. | Hodgkin’s disease, mixed cellularity | 13 |
| B6150 | Hodgkin’s disease, mixed cellularity of unspecified site | 0 |
| B6151 | Hodgkin’s disease, mixed cellularity of lymph nodes of head, face and neck | <5 |
| B6152 | Hodgkin’s disease, mixed cellularity of intrathoracic lymph nodes | 0 |
| B6153 | Hodgkin’s disease, mixed cellularity of intra-abdominal lymph nodes | 0 |
| B6154 | Hodgkin’s disease, mixed cellularity of lymph nodes of axilla and upper limb | <5 |
| B6155 | Hodgkin’s disease, mixed cellularity of lymph nodes of inguinal region and lower limb | 0 |
| B6156 | Hodgkin’s disease, mixed cellularity of intrapelvic lymph nodes | 0 |
| B6157 | Hodgkin’s disease, mixed cellularity of spleen | 0 |
| B6158 | Hodgkin’s disease, mixed cellularity of lymph nodes of multiple sites | 0 |
| B615z | Hodgkin’s disease, mixed cellularity NOS | 0 |
| B616. | Hodgkin’s disease, lymphocytic depletion | <5 |
| B6160 | Hodgkin’s lymphocytic depletion of unspecified site | <5 |
| B6161 | Hodgkin’s disease, lymphocytic depletion of lymph nodes of head, face and neck | <5 |
| B6162 | Hodgkin’s disease, lymphocytic depletion of intrathoracic lymph nodes | 0 |
| B6163 | Hodgkin’s disease, lymphocytic depletion of intra-abdominal lymph nodes | 0 |
| B6164 | Hodgkin’s disease, lymphocytic depletion of lymph nodes of axilla and upper limb | 0 |
| B6165 | Hodgkin’s disease, lymphocytic depletion of lymph nodes of inguinal region and lower limb | 0 |
| B6166 | Hodgkin’s disease, lymphocytic depletion of intrapelvic lymph nodes | 0 |
| B6167 | Hodgkin’s disease, lymphocytic depletion of spleen | <5 |
| B6168 | Hodgkin’s disease, lymphocytic depletion of lymph nodes of multiple sites | 0 |
| B616z | Hodgkin’s disease, lymphocytic depletion NOS | <5 |
| B617. | Nodular lymphocyte predominant Hodgkin lymphoma | <5 |
| B618. | Nodular sclerosis classical Hodgkin lymphoma | <5 |
| B619. | Mixed cellularity classical Hodgkin lymphoma | <5 |
| B61A. | Lymphocyte depleted classical Hodgkin lymphoma | 0 |
| B61B. | Lymphocyte-rich classical Hodgkin lymphoma | 0 |
| B61C. | Other classical Hodgkin lymphoma | 5 |
| B61z. | Hodgkin’s disease NOS | 44 |
| B61z0 | Hodgkin’s disease NOS, unspecified site | <5 |
| B61z1 | Hodgkin’s disease NOS of lymph nodes of head, face and neck | 5 |
| B61z2 | Hodgkin’s disease NOS of intrathoracic lymph nodes | <5 |
| B61z3 | Hodgkin’s disease NOS of intra-abdominal lymph nodes | 0 |
| B61z4 | Hodgkin’s disease NOS of lymph nodes of axilla and upper limb | 0 |
| B61z5 | Hodgkin’s disease NOS of lymph nodes of inguinal region and lower limb | <5 |
| B61z6 | Hodgkin’s disease NOS of intrapelvic lymph nodes | 0 |
| B61z7 | Hodgkin’s disease NOS of spleen | 0 |
| B61z8 | Hodgkin’s disease NOS of lymph nodes of multiple sites | <5 |
| B61zz | Hodgkin’s disease NOS | 15 |
| B62.. | Other malignant neoplasm of lymphoid and histiocytic tissue | 54 |
| B620. | Nodular lymphoma (Brill - Symmers disease) | 17 |
| B6200 | Nodular lymphoma of unspecified site | 9 |
| B6201 | Nodular lymphoma of lymph nodes of head, face and neck | 5 |
| B6202 | Nodular lymphoma of intrathoracic lymph nodes | <5 |
| B6203 | Nodular lymphoma of intra-abdominal lymph nodes | <5 |
| B6204 | Nodular lymphoma of lymph nodes of axilla and upper limb | <5 |
| B6205 | Nodular lymphoma of lymph nodes of inguinal region and lower limb | <5 |
| B6206 | Nodular lymphoma of intrapelvic lymph nodes | 0 |
| B6207 | Nodular lymphoma of spleen | 5 |
| B6208 | Nodular lymphoma of lymph nodes of multiple sites | <5 |
| B620z | Nodular lymphoma NOS | 13 |
| B621. | Mycosis fungoides | 321 |
| B6210 | Mycosis fungoides of unspecified site | <5 |
| B6211 | Mycosis fungoides of the lymph nodes of head, face and neck | <5 |
| B6212 | Mycosis fungoides of intrathoracic lymph nodes | 0 |
| B6213 | Mycosis fungoides of intra-abdominal lymph nodes | 0 |
| B6214 | Mycosis fungoides of lymph nodes of axilla and upper limb | <5 |
| B6215 | Mycosis fungoides of lymph nodes of inguinal region and lower limb | <5 |
| B6216 | Mycosis fungoides of intrapelvic lymph nodes | 0 |
| B6217 | Mycosis fungoides of spleen | 0 |
| B6218 | Mycosis fungoides of lymph nodes of multiple sites | 0 |
| B621z | Mycosis fungoides NOS | 12 |
| B622. | Sezary’s disease | 18 |
| B6220 | Sezary’s disease of unspecified site | 0 |
| B6221 | Sezary’s disease of lymph nodes of head, face and neck | 0 |
| B6222 | Sezary’s disease of intrathoracic lymph nodes | 0 |
| B6223 | Sezary’s disease of intra-abdominal lymph nodes | 0 |
| B6224 | Sezary’s disease of lymph nodes of axilla and upper limb | 0 |
| B6225 | Sezary’s disease of lymph nodes of inguinal region and lower limb | 0 |
| B6226 | Sezary’s disease of intrapelvic lymph nodes | 0 |
| B6227 | Sezary’s disease of spleen | 0 |
| B6228 | Sezary’s disease of lymph nodes of multiple sites | 0 |
| B622z | Sezary’s disease NOS | <5 |
| B623. | Malignant histiocytosis | 16 |
| B6230 | Malignant histiocytosis of unspecified site | 0 |
| B6231 | Malignant histiocytosis of lymph nodes of head, face and neck | <5 |
| B6232 | Malignant histiocytosis of intrathoracic lymph nodes | 0 |
| B6233 | Malignant histiocytosis of intra-abdominal lymph nodes | <5 |
| B6234 | Malignant histiocytosis of lymph nodes of axilla and upper limb | 0 |
| B6235 | Malignant histiocytosis of lymph nodes of inguinal region and lower limb | 0 |
| B6236 | Malignant histiocytosis of intrapelvic lymph nodes | 0 |
| B6237 | Malignant histiocytosis of spleen | 0 |
| B6238 | Malignant histiocytosis of lymph nodes of multiple sites | 0 |
| B623z | Malignant histiocytosis NOS | <5 |
| B624. | Leukaemic reticuloendotheliosis | 119 |
| B6240 | Leukaemic reticuloendotheliosis of unspecified sites | 0 |
| B6241 | Leukaemic reticuloendotheliosis of lymph nodes of head, face and neck | 0 |
| B6242 | Leukaemic reticuloendotheliosis of intrathoracic lymph nodes | 0 |
| B6243 | Leukaemic reticuloendotheliosis of intra-abdominal lymph nodes | 0 |
| B6244 | Leukaemic reticuloendotheliosis of lymph nodes of axilla and upper limb | 0 |
| B6245 | Leukaemic reticuloendotheliosis of lymph nodes of inguinal region and lower limb | 0 |
| B6246 | Leukaemic reticuloendotheliosis of intrapelvic lymph nodes | 0 |
| B6247 | Leukaemic reticuloendotheliosis of spleen | 0 |
| B6248 | Leukaemic reticuloendotheliosis of lymph nodes of multiple sites | 0 |
| B624z | Leukaemic reticuloendotheliosis NOS | 0 |
| B625. | Letterer-Siwe disease | 7 |
| B6250 | Letterer-Siwe disease of unspecified sites | 0 |
| B6251 | Letterer-Siwe disease of lymph nodes of head, face and neck | 0 |
| B6252 | Letterer-Siwe disease of intrathoracic lymph nodes | <5 |
| B6253 | Letterer-Siwe disease of intra-abdominal lymph nodes | 0 |
| B6254 | Letterer-Siwe disease of lymph nodes of axilla and upper limb | 0 |
| B6255 | Letterer-Siwe disease of lymph nodes of inguinal region and lower limb | 0 |
| B6256 | Letterer-Siwe disease of intrapelvic lymph nodes | 0 |
| B6257 | Letterer-Siwe disease of spleen | 0 |
| B6258 | Letterer-Siwe disease of lymph nodes of multiple sites | 0 |
| B625z | Letterer-Siwe disease NOS | <5 |
| B626. | Malignant mast cell tumours | 8 |
| B6260 | Mast cell malignancy of unspecified site | <5 |
| B6261 | Mast cell malignancy of lymph nodes of head, face and neck | <5 |
| B6262 | Mast cell malignancy of intrathoracic lymph nodes | 0 |
| B6263 | Mast cell malignancy of intra-abdominal lymph nodes | 0 |
| B6264 | Mast cell malignancy of lymph nodes of axilla and upper limb | 0 |
| B6265 | Mast cell malignancy of lymph nodes of inguinal region and lower limb | <5 |
| B6266 | Mast cell malignancy of intrapelvic lymph nodes | 0 |
| B6267 | Mast cell malignancy of spleen | 0 |
| B6268 | Mast cell malignancy of lymph nodes of multiple sites | <5 |
| B626z | Malignant mast cell tumour NOS | 0 |
| B627. | Non - Hodgkin’s lymphoma | 2707 |
| B6270 | Follicular non-Hodgkin’s small cleaved cell lymphoma | 19 |
| B6271 | Follicular non-Hodgkin’s mixed small cleaved and large cell lymphoma | <5 |
| B6272 | Follicular non-Hodgkin’s large cell lymphoma | 16 |
| B6273 | Diffuse non-Hodgkin’s small cell (diffuse) lymphoma | 9 |
| B6274 | Diffuse non-Hodgkin’s small cleaved cell (diffuse) lymphoma | 0 |
| B6275 | Diffuse non-Hodgkin’s mixed small and large cell (diffuse) lymphoma | 6 |
| B6276 | Diffuse non-Hodgkin’s immunoblastic (diffuse) lymphoma | 0 |
| B6277 | Diffuse non-Hodgkin’s lymphoblastic (diffuse) lymphoma | 6 |
| B6278 | Diffuse non-Hodgkin’s lymphoma undifferentiated (diffuse) | <5 |
| B6279 | Mucosa-associated lymphoma | 30 |
| B627A | Diffuse non-Hodgkin’s large cell lymphoma | 44 |
| B627B | Other types of follicular non-Hodgkin’s lymphoma | 16 |
| B627C | Follicular non-Hodgkin’s lymphoma | 574 |
| B627D | Diffuse non-Hodgkin’s centroblastic lymphoma | <5 |
| B627E | Diffuse large B-cell lymphoma | 320 |
| B627F | Extranodal marginal zone B-cell lymphoma of mucosa-associated lymphoid tissue | <5 |
| B627G | Mediastinal (thymic) large B-cell lymphoma | <5 |
| B627W | Unspecified B-cell non-Hodgkin’s lymphoma | 257 |
| B627X | Diffuse non-Hodgkin’s lymphoma, unspecified | 43 |
| B628. | Follicular lymphoma | 187 |
| B6280 | Follicular lymphoma grade 1 | 8 |
| B6281 | Follicular lymphoma grade 2 | 6 |
| B6282 | Follicular lymphoma grade 3 | <5 |
| B6283 | Follicular lymphoma grade 3a | <5 |
| B6284 | Follicular lymphoma grade 3b | <5 |
| B6285 | Diffuse follicle centre lymphoma | <5 |
| B6286 | Cutaneous follicle centre lymphoma | <5 |
| B6287 | Other types of follicular lymphoma | <5 |
| B629. | Multifocal and multisystemic (disseminated) Langerhans-cell histiocytosis | 0 |
| B62A. | Sarcoma of dendritic cells | <5 |
| B62B. | Multifocal and unisystemic Langerhans-cell histiocytosis | 0 |
| B62C. | Unifocal Langerhans-cell histiocytosis | 0 |
| B62D. | Histiocytic sarcoma | <5 |
| B62E. | T/NK-cell lymphoma | 11 |
| B62E0 | Mature T/NK-cell lymphoma | 0 |
| B62E1 | Anaplastic large cell lymphoma, ALK-positive | <5 |
| B62E2 | Anaplastic large cell lymphoma, ALK-negative | <5 |
| B62E3 | Cutaneous T-cell lymphoma | 21 |
| B62E4 | Extranodal NK/T-cell lymphoma, nasal type | <5 |
| B62E5 | Hepatosplenic T-cell lymphoma | <5 |
| B62E6 | Enteropathy-associated T-cell lymphoma | <5 |
| B62E7 | Subcutaneous panniculitic T-cell lymphoma | <5 |
| B62E8 | Blastic NK-cell lymphoma | <5 |
| B62E9 | Angioimmunoblastic T-cell lymphoma | 11 |
| B62EA | Primary cutaneous CD30-positive T-cell proliferations | <5 |
| B62Ew | Other mature T/NK-cell lymphoma | <5 |
| B62F. | Nonfollicular lymphoma | <5 |
| B62F0 | Small cell B-cell lymphoma | 53 |
| B62F1 | Mantle cell lymphoma | 106 |
| B62F2 | Lymphoblastic (diffuse) lymphoma | <5 |
| B62Fy | Other non-follicular lymphoma | <5 |
| B62x. | Malignant lymphoma otherwise specified | 150 |
| B62x0 | T-zone lymphoma | <5 |
| B62x1 | Lymphoepithelioid lymphoma | <5 |
| B62x2 | Peripheral T-cell lymphoma | 36 |
| B62x3 | Malignant reticuloendotheliosis | <5 |
| B62x4 | Malignant reticulosis | 7 |
| B62x5 | Malignant immunoproliferative small intestinal disease | 0 |
| B62x6 | True histiocytic lymphoma | <5 |
| B62xX | Other and unspecified peripheral and cutaneous T-cell lymphomas | 48 |
| B62y. | Malignant lymphoma NOS | 372 |
| B62y0 | Malignant lymphoma NOS of unspecified site | 9 |
| B62y1 | Malignant lymphoma NOS of lymph nodes of head, face and neck | 26 |
| B62y2 | Malignant lymphoma NOS of intrathoracic lymph nodes | 5 |
| B62y3 | Malignant lymphoma NOS of intra-abdominal lymph nodes | 7 |
| B62y4 | Malignant lymphoma NOS of lymph nodes of axilla and upper limb | <5 |
| B62y5 | Malignant lymphoma NOS of lymph nodes of inguinal region and lower limb | 5 |
| B62y6 | Malignant lymphoma NOS of intrapelvic lymph nodes | <5 |
| B62y7 | Malignant lymphoma NOS of spleen | 11 |
| B62y8 | Malignant lymphoma NOS of lymph nodes of multiple sites | 15 |
| B62yz | Malignant lymphoma NOS | 123 |
| B62z. | Malignant neoplasms of lymphoid and histiocytic tissue NOS | <5 |
| B62z0 | Unspecified malignant neoplasm of lymphoid and histiocytic tissue of unspecified site | 0 |
| B62z1 | Unspecified malignant neoplasm of lymphoid and histiocytic tissue of lymph nodes of head, face and neck | 5 |
| B62z2 | Unspecified malignant neoplasm of lymphoid and histiocytic tissue of intrathoracic lymph nodes | 0 |
| B62z3 | Unspecified malignant neoplasm of lymphoid and histiocytic tissue of intra-abdominal lymph nodes | <5 |
| B62z4 | Unspecified malignant neoplasm of lymphoid and histiocytic tissue of lymph nodes of axilla and upper limb | <5 |
| B62z5 | Unspecified malignant neoplasm of lymphoid and histiocytic tissue of lymph nodes of inguinal region and lower limb | <5 |
| B62z6 | Unspecified malignant neoplasm of lymphoid and histiocytic tissue of intrapelvic lymph nodes | 0 |
| B62z7 | Unspecified malignant neoplasm of lymphoid and histiocytic tissue of spleen | <5 |
| B62z8 | Unspecified malignant neoplasm of lymphoid and histiocytic tissue of lymph nodes of multiple sites | <5 |
| B62zz | Lymphoid and histiocytic malignancy NOS | <5 |
| B630. | Multiple myeloma | 2563 |
| B6304 | Solitary plasmacytoma | 7 |
| B640. | Acute lymphoid leukaemia | 95 |
| B6400 | B-cell acute lymphoblastic leukaemia | 11 |
| B641. | Chronic lymphoid leukaemia | 3813 |
| B6410 | B-cell chronic lymphocytic leukaemia | 76 |
| B6411 | Clinical stage A chronic lymphocytic leukaemia | 74 |
| B6412 | Clinical stage B chronic lymphocytic leukaemia | <5 |
| B6413 | Clinical stage C chronic lymphocytic leukaemia | 8 |
| B650. | Acute myeloid leukaemia | 1183 |
| B6500 | Acute myeloid leukaemia with 11q23 abnormality | 0 |
| B6501 | Acute myeloid leukaemia with multilineage dysplasia | 0 |
| B651. | Chronic myeloid leukaemia | 484 |
| B6510 | Chronic eosinophilic leukaemia | 0 |
| B6511 | Chronic myeloid leukaemia, BCR/ABL positive | <5 |
| B6512 | Chronic neutrophilic leukaemia | 5 |
| B6513 | Atypical chronic myeloid leukaemia, BCR/ABL negative | 0 |
| B651z | Chronic myeloid leukaemia NOS | 27 |
| B12I. | NA | 117 |

### ICD 9 and 10 codes:

| code | desc | total\_n |
| --- | --- | --- |
| 140 | Malignant neoplasm of lip | 0 |
| 1400 | Upper lip vermilion border | 0 |
| 1401 | Lower lip vermilion border | <5 |
| 1403 | Upper lip inner aspect | 0 |
| 1404 | Lower lip inner aspect | 0 |
| 1405 | Lip unspecified inner aspect | 0 |
| 1406 | Commissure of lip | 0 |
| 1408 | Other | 0 |
| 1409 | Lip unspecified vermilion border | 8 |
| 141 | Malignant neoplasm of tongue | 0 |
| 1410 | Base of tongue | <5 |
| 1411 | Dorsal surface of tongue | 0 |
| 1412 | Tip and lateral border of tongue | 0 |
| 1413 | Ventral surface of tongue | 0 |
| 1414 | Anterior two-thirds of tongue part unspecified | 0 |
| 1415 | Junctional zone | 0 |
| 1416 | Lingual tonsil | 0 |
| 1418 | Other | 0 |
| 1419 | Tongue unspecified | 48 |
| 142 | Malignant neoplasm of major salivary glands | 0 |
| 1420 | Parotid gland | 17 |
| 1421 | Submandibular gland | <5 |
| 1422 | Sublingual gland | 0 |
| 1428 | Other | 0 |
| 1429 | Site unspecified | <5 |
| 143 | Malignant neoplasm of gum | 0 |
| 1430 | Upper gum | 5 |
| 1431 | Lower gum | 7 |
| 1438 | Other | 0 |
| 1439 | Gum unspecified | <5 |
| 144 | Malignant neoplasm of floor of mouth | 0 |
| 1440 | Anterior portion | 0 |
| 1441 | Lateral portion | 0 |
| 1448 | Other | 0 |
| 1449 | Part unspecified | 17 |
| 145 | Malignant neoplasm of other and unspecified parts | 0 |
| 1450 | Cheek mucosa | <5 |
| 1451 | Vestibule of mouth | 0 |
| 1452 | Hard palate | <5 |
| 1453 | Soft palate | 5 |
| 1454 | Uvula | 0 |
| 1455 | Palate unspecified | <5 |
| 1456 | Retromolar area | <5 |
| 1458 | Other | 0 |
| 1459 | Mouth unspecified | 28 |
| 146 | Malignant neoplasm of oropharynx | 0 |
| 1460 | Tonsil | 29 |
| 1461 | Tonsillar fossa | <5 |
| 1462 | Tonsillar pillars (anterior) (posterior) | 0 |
| 1463 | Vallecula | 0 |
| 1464 | Anterior aspect of epiglottis | 0 |
| 1465 | Junctional region | 0 |
| 1466 | Lateral wall of oropharynx | 0 |
| 1467 | Posterior wall of oropharynx | 0 |
| 1468 | Other | 0 |
| 1469 | Oropharynx unspecified | 12 |
| 147 | Malignant neoplasm of nasopharynx | 0 |
| 1470 | Superior wall | 0 |
| 1471 | Posterior wall | <5 |
| 1472 | Lateral wall | 0 |
| 1473 | Anterior wall | 0 |
| 1478 | Other | 0 |
| 1479 | Nasopharynx unspecified | 12 |
| 148 | Malignant neoplasm of hypopharynx | 0 |
| 1480 | Postcricoid region | <5 |
| 1481 | Pyriform sinus | 10 |
| 1482 | Aryepiglottic fold hypopharyngeal aspect | <5 |
| 1483 | Posterior hypopharyngeal wall | 0 |
| 1488 | Other | 0 |
| 1489 | Hypopharynx unspecified | <5 |
| 149 | Malignant neoplasm of other and ill-defined sites | 0 |
| 1490 | Pharynx unspecified | 38 |
| 1491 | Waldeyer s ring | 0 |
| 1498 | Other | <5 |
| 1499 | Ill-defined | 0 |
| 150 | Malignant neoplasm of oesophagus | 0 |
| 1500 | Cervical part | 0 |
| 1501 | Thoracic part | <5 |
| 1502 | Abdominal part | 0 |
| 1503 | Upper third | <5 |
| 1504 | Middle third | <5 |
| 1505 | Lower third | 13 |
| 1508 | Other | 0 |
| 1509 | Oesophagus unspecified | 824 |
| 151 | Malignant neoplasm of stomach | 0 |
| 1510 | Cardia | 41 |
| 1511 | Pylorus | <5 |
| 1512 | Pyloric antrum | 0 |
| 1513 | Fundus of stomach | <5 |
| 1514 | Body of stomach | <5 |
| 1515 | Lesser curvature unspecified | <5 |
| 1516 | Greater curvature unspecified | 0 |
| 1518 | Other | 0 |
| 1519 | Stomach unspecified | 941 |
| 152 | Malignant neoplasm of small intestine including d | 0 |
| 1520 | Duodenum | 22 |
| 1521 | Jejunum | <5 |
| 1522 | Ileum | <5 |
| 1523 | Meckel s diverticulum | 0 |
| 1528 | Other | 0 |
| 1529 | Small intestine unspecified | 7 |
| 153 | Malignant neoplasm of colon | 0 |
| 1530 | Hepatic flexure | 0 |
| 1531 | Transverse colon | 16 |
| 1532 | Descending colon | 7 |
| 1533 | Sigmoid colon | 136 |
| 1534 | Caecum | 194 |
| 1535 | Appendix | 7 |
| 1536 | Ascending colon | 14 |
| 1537 | Splenic flexure | 6 |
| 1538 | Other colon | 0 |
| 1539 | Colon unspecified | 1299 |
| 154 | Malignant neoplasm of rectum rectosigmoid junctio | 0 |
| 1540 | Rectosigmoid junction | 71 |
| 1541 | Rectum | 578 |
| 1542 | Anal canal | <5 |
| 1543 | Anus unspecified | 20 |
| 1548 | Other | <5 |
| 155 | Malignant neoplasm of liver and intrahepatic bile | 0 |
| 1550 | Liver primary | 76 |
| 1551 | Intrahepatic bile ducts | 130 |
| 1552 | Liver not specified as primary or secondary | 53 |
| 156 | Malignant neoplasm of gallbladder and extrahepatic | 0 |
| 1560 | Gallbladder | 35 |
| 1561 | Extrahepatic bile ducts | 21 |
| 1562 | Ampulla of vater | 6 |
| 1568 | Other | 0 |
| 1569 | Biliary tract part unspecified | <5 |
| 157 | Malignant neoplasm of pancreas | 0 |
| 1570 | Head of pancreas | 110 |
| 1571 | Body of pancreas | <5 |
| 1572 | Tail of pancreas | <5 |
| 1573 | Pancreatic duct | 0 |
| 1574 | Islets of langerhans | <5 |
| 1578 | Other | 0 |
| 1579 | Part unspecified | 656 |
| 158 | Malignant neoplasm of retroperitoneum and peritone | 0 |
| 1580 | Retroperitoneum | 7 |
| 1588 | Specified parts of peritoneum | <5 |
| 1589 | Peritoneum unspecified | 8 |
| 159 | Malignant neo other ill-def sites digest orgs and peritonum | 0 |
| 1590 | Intestinal tract part unspecified | 166 |
| 1591 | Spleen not elsewhere classified | 0 |
| 1598 | Other | 0 |
| 1599 | Ill-defined | 22 |
| 160 | Malignant neoplasm of nasal cavities middle ear a | 0 |
| 1600 | Nasal cavities | <5 |
| 1601 | Auditory tube middle ear and mastoid air cells | <5 |
| 1602 | Maxillary sinus | 7 |
| 1603 | Ethmoidal sinus | <5 |
| 1604 | Frontal sinus | <5 |
| 1605 | Sphenoidal sinus | <5 |
| 1608 | Other | <5 |
| 1609 | Accessory sinus unspecified | <5 |
| 161 | Malignant neoplasm of larynx | 0 |
| 1610 | Glottis | 7 |
| 1611 | Supraglottis | 12 |
| 1612 | Subglottis | <5 |
| 1613 | Laryngeal cartilages | 0 |
| 1618 | Other | 0 |
| 1619 | Larynx unspecified | 130 |
| 162 | Malignant neoplasm of trachea bronchus and lung | 0 |
| 1620 | Trachea | 5 |
| 1622 | Main bronchus | 10 |
| 1623 | Upper lobe bronchus or lung | 20 |
| 1624 | Middle lobe bronchus or lung | <5 |
| 1625 | Lower lobe bronchus or lung | 15 |
| 1628 | Other | 0 |
| 1629 | Bronchus and lung unspecified | 3724 |
| 163 | Malignant neoplasm of pleura | 0 |
| 1630 | Parietal | 0 |
| 1631 | Visceral | 0 |
| 1638 | Other | 0 |
| 1639 | Pleura unspecified | 40 |
| 164 | Malignant neoplasm of thymus heart and mediastinu | 0 |
| 1640 | Thymus | 5 |
| 1641 | Heart | <5 |
| 1642 | Anterior mediastinum | 0 |
| 1643 | Posterior mediastinum | 0 |
| 1648 | Other | 0 |
| 1649 | Mediastinum part unspecified | <5 |
| 165 | Other malignant neoplasms within the respiratory s | 0 |
| 1650 | Upper respiratory tract part unspecified | 0 |
| 1658 | Other | 0 |
| 1659 | Ill-defined sites within the respiratory system | 0 |
| 170 | Malignant neoplasm of bone and articular cartilage | 0 |
| 1700 | Bones of skull and face | <5 |
| 1701 | Lower jaw bone | <5 |
| 1702 | Vertebral column excluding sacrum and coccyx | 5 |
| 1703 | Ribs sternum and clavicle | <5 |
| 1704 | Long bones of upper limb and scapula | 0 |
| 1705 | Upper limb short bones | 0 |
| 1706 | Pelvic bones sacrum and coccyx | <5 |
| 1707 | Lower limb long bones | <5 |
| 1708 | Lower limb short bones | 0 |
| 1709 | Site unspecified | 5 |
| 171 | Malignant neoplasm of connective and other soft ti | 0 |
| 1710 | Head face and neck | <5 |
| 1712 | Upper limb including shoulder | <5 |
| 1713 | Lower limb including hip | 8 |
| 1714 | Thorax | <5 |
| 1715 | Abdomen | <5 |
| 1716 | Pelvis | <5 |
| 1717 | Trunk unspecified | 0 |
| 1718 | Other | 0 |
| 1719 | Site unspecified | 37 |
| 172 | Malignant melanoma of skin | 0 |
| 1720 | Lip | 0 |
| 1721 | Eyelid including canthus | <5 |
| 1722 | Ear and external auricular canal | <5 |
| 1723 | Other and unspecified parts of face | <5 |
| 1724 | Scalp and neck | 0 |
| 1725 | Trunk except scrotum | <5 |
| 1726 | Upper limb including shoulder | <5 |
| 1727 | Lower limb including hip | <5 |
| 1728 | Other | 0 |
| 1729 | Site unspecified | 165 |
| 173 | Other malignant neoplasm of skin | 0 |
| 1730 | Skin of lip | 0 |
| 1731 | Eyelid including canthus | <5 |
| 1732 | Ear and external auricular canal | 22 |
| 1733 | Skin of other and unspecified parts of face | 20 |
| 1734 | Scalp and skin of neck | 17 |
| 1735 | Skin of trunk except scrotum | 9 |
| 1736 | Skin of upper limb including shoulder | 6 |
| 1737 | Skin of lower limb including hip | 8 |
| 1738 | Other | 0 |
| 1739 | Site unspecified | 30 |
| 174 | Malignant neoplasm of female breast | 0 |
| 1740 | Nipple and areola | <5 |
| 1741 | Central portion | <5 |
| 1742 | Upper-inner quadrant | 0 |
| 1743 | Lower-inner quadrant | 0 |
| 1744 | Upper-outer quadrant | <5 |
| 1745 | Lower-outer quadrant | <5 |
| 1746 | Axillary tail | 0 |
| 1748 | Other | <5 |
| 1749 | Breast unspecified | 1772 |
| 175 | Malignant neoplasm of male breast | 9 |
| 179 | Malignant neoplasm of uterus part unspecified | 91 |
| 180 | Malignant neoplasm of cervix uteri | 0 |
| 1800 | Endocervix | <5 |
| 1801 | Exocervix | 0 |
| 1808 | Other | 0 |
| 1809 | Cervix uteri unspecified | 157 |
| 181 | Malignant neoplasm of placenta | 0 |
| 182 | Malignant neoplasm of body of uterus | 0 |
| 1820 | Corpus uteri except isthmus | 127 |
| 1821 | Isthmus | 0 |
| 1828 | Other | 0 |
| 183 | Malignant neoplasm of ovary and other uterine adne | 0 |
| 1830 | Ovary | 569 |
| 1832 | Fallopian tube | <5 |
| 1833 | Broad ligament | 0 |
| 1834 | Parametrium | 0 |
| 1835 | Round ligament | 0 |
| 1838 | Other | 0 |
| 1839 | Uterine adnexa unspecified | <5 |
| 184 | Malignant neoplasm of other and unspecified female | 0 |
| 1840 | Vagina | 17 |
| 1841 | Labia majora | <5 |
| 1842 | Labia minora | 0 |
| 1843 | Clitoris | 0 |
| 1844 | Vulva unspecified | 66 |
| 1848 | Other | 0 |
| 1849 | Site unspecified | 0 |
| 185 | Malignant neoplasm of prostate | 1392 |
| 186 | Malignant neoplasm of testis | 0 |
| 1860 | Undescended | 0 |
| 1869 | Other and unspecified | 9 |
| 187 | Malignant neoplasm of penis and other male genital | 0 |
| 1871 | Prepuce | 0 |
| 1872 | Glans penis | 0 |
| 1873 | Body of penis | <5 |
| 1874 | Penis part unspecified | 14 |
| 1875 | Epididymis | 0 |
| 1876 | Spermatic cord | 0 |
| 1877 | Scrotum | 5 |
| 1878 | Other | 0 |
| 1879 | Site unspecified | <5 |
| 188 | Malignant neoplasm of bladder | 0 |
| 1880 | Trigone | 0 |
| 1881 | Dome | 0 |
| 1882 | Lateral wall | <5 |
| 1883 | Anterior wall | <5 |
| 1884 | Posterior wall | <5 |
| 1885 | Bladder neck | <5 |
| 1886 | Ureteric orifice | <5 |
| 1887 | Urachus | 0 |
| 1888 | Other | 0 |
| 1889 | Part unspecified | 608 |
| 189 | Malignant neoplasm of kidney and other and unspeci | 0 |
| 1890 | Kidney except pelvis | 401 |
| 1891 | Renal pelvis | 5 |
| 1892 | Ureter | 22 |
| 1893 | Urethra | 5 |
| 1894 | Paraurethral glands | 0 |
| 1898 | Other | 0 |
| 1899 | Site unspecified | 0 |
| 190 | Malignant neoplasm of eye | 0 |
| 1900 | Eyeball except conjunctiva cornea retina and ch | 0 |
| 1901 | Orbit | <5 |
| 1902 | Lacrimal gland | 0 |
| 1903 | Conjunctiva | <5 |
| 1904 | C0rnea | 0 |
| 1905 | Retina | <5 |
| 1906 | Choroid | <5 |
| 1907 | Lacrimal duct | 0 |
| 1908 | Other | 0 |
| 1909 | Part unspecified | 10 |
| 191 | Malignant neoplasm of brain | 0 |
| 1910 | Cerebrum except lobes and ventricles | 54 |
| 1911 | Frontal lobe | 25 |
| 1912 | Temporal lobe | 12 |
| 1913 | Parietal lobe | 14 |
| 1914 | Occipital lobe | <5 |
| 1915 | Ventricle | 0 |
| 1916 | Cerebellum | <5 |
| 1917 | Brain stem | <5 |
| 1918 | Other | 13 |
| 1919 | Brain unspecified | 204 |
| 192 | Malignant neo other and unspec parts of nervous system | 0 |
| 1920 | Cranial nerves | 0 |
| 1921 | Cerebral meninges | <5 |
| 1922 | Spinal cord | <5 |
| 1923 | Spinal meninges | 0 |
| 1928 | Other | 0 |
| 1929 | Part unspecified | <5 |
| 193 | Malignant neoplasm of thyroid gland | 42 |
| 194 | Malignant neoplasm of other endocrine glands and r | 0 |
| 1940 | Suprarenal gland | 8 |
| 1941 | Parathyroid gland | 0 |
| 1943 | Pituitary gland and craniopharyngeal duct | <5 |
| 1944 | Pineal gland | 0 |
| 1945 | Carotid body | <5 |
| 1946 | Aortic body and other paraganglia | <5 |
| 1947 | NA | <5 |
| 1948 | Other | 0 |
| 1949 | Site unspecified | <5 |
| 195 | Malignant neoplasm of other and ill-defined sites | 0 |
| 1950 | Head face and neck | 22 |
| 1951 | Thorax | 6 |
| 1952 | Abdomen | 34 |
| 1953 | Pelvis | 18 |
| 1954 | Upper limb | 0 |
| 1955 | Lower limb | <5 |
| 1958 | Other specified sites | 7 |
| 196 | Secondary and unspecified malignant neoplasm of ly | 0 |
| 1960 | Head face and neck | 6 |
| 1961 | Intrathoracic | <5 |
| 1962 | Intra-abdominal | <5 |
| 1963 | Axilla and upper limb | 10 |
| 1965 | Inguinal and lower limb | 0 |
| 1966 | Intrapelvic | 0 |
| 1968 | Multiple sites | 0 |
| 1969 | Site unspecified | 14 |
| 196X | NA | <5 |
| 197 | Secondary malignant neoplasm of respiratory and di | 0 |
| 1970 | Lung | 146 |
| 1971 | Mediastinum | <5 |
| 1972 | Pleura | 7 |
| 1973 | Other respiratory organs | 5 |
| 1974 | Small intestine including duodenum | <5 |
| 1975 | Large intestine and rectum | 14 |
| 1976 | Retroperitoneum and peritoneum | 25 |
| 1977 | Liver | 516 |
| 1978 | Other digestive organs | 25 |
| 198 | Secondary malignant neoplasm of other specified si | 0 |
| 1980 | Kidney | <5 |
| 1981 | Other urinary organs | 14 |
| 1982 | Skin | 8 |
| 1983 | Brain and spinal cord | 257 |
| 1984 | Other parts of nervous system | <5 |
| 1985 | Bone and bone marrow | 159 |
| 1986 | Ovary | 8 |
| 1987 | Suprarenal gland | 6 |
| 1988 | Other specified sites | 97 |
| 199 | Malignant neoplasm without specification of site | 0 |
| 1990 | Disseminated | 6034 |
| 1991 | Other without specification of site | 1774 |
| 200 | Lymphosarcoma and reticulosarcoma | 0 |
| 2000 | Reticulosarcoma | 0 |
| 2001 | Lymphosarcoma | <5 |
| 2002 | Burkitt s tumour | 5 |
| 2008 | Other named variants | <5 |
| 201 | Hodgkin s disease | 0 |
| 2010 | Hodgkin s paragranuloma | 0 |
| 2011 | Hodgkin s granuloma | 0 |
| 2012 | Hodgkin s sarcoma | 0 |
| 2014 | Lymphocytic-histiocytic predominance | 0 |
| 2015 | Nodular sclerosis | <5 |
| 2016 | Mixed cellularity | 0 |
| 2017 | Lymphocytic depletion | 0 |
| 2019 | Unspecified | 35 |
| 202 | Other malignant neoplasm of lymphoid and histiocyt | 0 |
| 2020 | Nodular lymphoma | 5 |
| 2021 | Mycosis fungoides | 5 |
| 2022 | Sezary s disease | <5 |
| 2023 | Malignant histiocytosis | 0 |
| 2024 | Leukaemic reticuloendotheliosis | 8 |
| 2025 | Letterer-siwe disease | 0 |
| 2026 | Malignant mast-cell tumours | 0 |
| 2028 | Other lymphomas | 500 |
| 2029 | Other and unspecified | 0 |
| 203 | Multiple myeloma and immunoproliferative neoplasms | 0 |
| 2030 | Multiple myeloma | 312 |
| 2031 | Plasma cell leukaemia | <5 |
| 2038 | Other immunoproliferative neoplasms | <5 |
| 204 | Lymphoid leukaemia | 0 |
| 2040 | Acute lymphoid leukaemia | 24 |
| 2041 | Chronic lymphoid leukaemia | 186 |
| 2042 | Subacute | 0 |
| 2048 | Other | 0 |
| 2049 | Unspecified | 7 |
| 205 | Myeloid leukaemia | 0 |
| 2050 | Acute myeloid leukaemia | 221 |
| 2051 | Chronic myeloid leukaemia | 78 |
| 2052 | Subacute | 0 |
| 2053 | Myeloid sarcoma | 0 |
| 2058 | Other | 0 |
| 2059 | Unspecified | 7 |
| 206 | Monocytic leukaemia | 0 |
| 2060 | Acute monocytic leukaemia | <5 |
| 2061 | Chronic monocytic leukaemia | <5 |
| 2062 | Subacute | 0 |
| 2068 | Other | 0 |
| 2069 | Unspecified | 0 |
| 207 | Other specified leukaemia | 0 |
| 2070 | Acute erythraemia and erythroleukaemia | 0 |
| 2071 | Chronic erythraemia | 0 |
| 2072 | Megakaryocytic leukaemia | 0 |
| 2078 | Other | <5 |
| 208 | Leukaemia of unspecified cell type | 0 |
| 2080 | Acute leukaemia of unspecified cell type | 28 |
| 2081 | Chronic leukaemia of unspecified cell type | 0 |
| 2082 | Subacute | 0 |
| 2088 | Other | 0 |
| 2089 | Unspecified | 18 |
| C00 | Malignant neoplasm of lip | 0 |
| C000 | Malignant neoplasm: External upper lip | 146 |
| C001 | Malignant neoplasm: External lower lip | 280 |
| C002 | Malignant neoplasm: External lip unspecified | 8 |
| C003 | Malignant neoplasm: Upper lip inner aspect | 13 |
| C004 | Malignant neoplasm: Lower lip inner aspect | 19 |
| C005 | Malignant neoplasm: Lip unspecified inner aspect | <5 |
| C006 | Malignant neoplasm: Commissure of lip | 19 |
| C008 | Malignant neoplasm: Overlapping lesion of lip | <5 |
| C009 | Malignant neoplasm: Lip unspecified | 136 |
| C01 | Malignant neoplasm of base of tongue | 71 |
| C01X | NA | 719 |
| C02 | Malignant neoplasm of other and unspecified parts of tongue | 0 |
| C020 | Malignant neoplasm: Dorsal surface of tongue | 34 |
| C021 | Malignant neoplasm: Border of tongue | 348 |
| C022 | Malignant neoplasm: Ventral surface of tongue | 91 |
| C023 | Malignant neoplasm: Anterior two-thirds of tongue part unspecified | 104 |
| C024 | Malignant neoplasm: Lingual tonsil | 89 |
| C028 | Malignant neoplasm: Overlapping lesion of tongue | 42 |
| C029 | Malignant neoplasm: Tongue unspecified | 762 |
| C02X | NA | <5 |
| C03 | Malignant neoplasm of gum | 0 |
| C030 | Malignant neoplasm: Upper gum | 102 |
| C031 | Malignant neoplasm: Lower gum | 123 |
| C039 | Malignant neoplasm: Gum unspecified | 62 |
| C04 | Malignant neoplasm of floor of mouth | 0 |
| C040 | Malignant neoplasm: Anterior floor of mouth | 153 |
| C041 | Malignant neoplasm: Lateral floor of mouth | 12 |
| C048 | Malignant neoplasm: Overlapping lesion of floor of mouth | 9 |
| C049 | Malignant neoplasm: Floor of mouth unspecified | 396 |
| C05 | Malignant neoplasm of palate | 0 |
| C050 | Malignant neoplasm: Hard palate | 65 |
| C051 | Malignant neoplasm: Soft palate | 159 |
| C052 | Malignant neoplasm: Uvula | 38 |
| C058 | Malignant neoplasm: Overlapping lesion of palate | 34 |
| C059 | Malignant neoplasm: Palate unspecified | 115 |
| C05X | NA | <5 |
| C06 | Malignant neoplasm of other and unspecified parts of mouth | 0 |
| C060 | Malignant neoplasm: Cheek mucosa | 154 |
| C061 | Malignant neoplasm: Vestibule of mouth | 25 |
| C062 | Malignant neoplasm: Retromolar area | 165 |
| C068 | Malignant neoplasm: Overlapping lesion of other and unspecified parts of mouth | 19 |
| C069 | Malignant neoplasm: Mouth unspecified | 276 |
| C07 | Malignant neoplasm of parotid gland | 155 |
| C07. | NA | <5 |
| C07X | NA | 531 |
| C08 | Malignant neoplasm of other and unspecified major salivary glands | 0 |
| C080 | Malignant neoplasm: Submandibular gland | 106 |
| C081 | Malignant neoplasm: Sublingual gland | 6 |
| C088 | Malignant neoplasm: Overlapping lesion of major salivary glands | <5 |
| C089 | Malignant neoplasm: Major salivary gland unspecified | 49 |
| C09 | Malignant neoplasm of tonsil | 0 |
| C090 | Malignant neoplasm: Tonsillar fossa | 101 |
| C091 | Malignant neoplasm: Tonsillar pillar (anterior)(posterior) | 43 |
| C098 | Malignant neoplasm: Overlapping lesion of tonsil | 20 |
| C099 | Malignant neoplasm: Tonsil unspecified | 914 |
| C10 | Malignant neoplasm of oropharynx | 0 |
| C100 | Malignant neoplasm: Vallecula | 51 |
| C101 | Malignant neoplasm: Anterior surface of epiglottis | 25 |
| C102 | Malignant neoplasm: Lateral wall of oropharynx | 22 |
| C103 | Malignant neoplasm: Posterior wall of oropharynx | 40 |
| C104 | Malignant neoplasm: Branchial cleft | 18 |
| C108 | Malignant neoplasm: Overlapping lesion of oropharynx | 23 |
| C109 | Malignant neoplasm: Oropharynx unspecified | 378 |
| C11 | Malignant neoplasm of nasopharynx | 0 |
| C110 | Malignant neoplasm: Superior wall of nasopharynx | 5 |
| C111 | Malignant neoplasm: Posterior wall of nasopharynx | 38 |
| C112 | Malignant neoplasm: Lateral wall of nasopharynx | 11 |
| C113 | Malignant neoplasm: Anterior wall of nasopharynx | 13 |
| C118 | Malignant neoplasm: Overlapping lesion of nasopharynx | 10 |
| C119 | Malignant neoplasm: Nasopharynx unspecified | 183 |
| C11X | NA | <5 |
| C12 | Malignant neoplasm of piriform sinus | 69 |
| C12X | NA | 372 |
| C13 | Malignant neoplasm of hypopharynx | 0 |
| C130 | Malignant neoplasm: Postcricoid region | 120 |
| C131 | Malignant neoplasm: Aryepiglottic fold hypopharyngeal aspect | 25 |
| C132 | Malignant neoplasm: Posterior wall of hypopharynx | 27 |
| C138 | Malignant neoplasm: Overlapping lesion of hypopharynx | 12 |
| C139 | Malignant neoplasm: Hypopharynx unspecified | 231 |
| C14 | Malignant neoplasm of other and ill-defined sites in the lip oral cavity and pharynx | 0 |
| C140 | Malignant neoplasm: Pharynx unspecified | 531 |
| C141 | NA | <5 |
| C142 | Malignant neoplasm: Waldeyer ring | 0 |
| C148 | Malignant neoplasm: Overlapping lesion of lip oral cavity and pharynx | 37 |
| C15 | Malignant neoplasm of oesophagus | 0 |
| C15. | NA | <5 |
| C150 | Malignant neoplasm: Cervical part of oesophagus | 57 |
| C151 | Malignant neoplasm: Thoracic part of oesophagus | 40 |
| C152 | Malignant neoplasm: Abdominal part of oesophagus | 38 |
| C153 | Malignant neoplasm: Upper third of oesophagus | 536 |
| C154 | Malignant neoplasm: Middle third of oesophagus | 1365 |
| C155 | Malignant neoplasm: Lower third of oesophagus | 4463 |
| C158 | Malignant neoplasm: Overlapping lesion of oesophagus | 198 |
| C159 | Malignant neoplasm: Oesophagus unspecified | 7839 |
| C16 | Malignant neoplasm of stomach | 0 |
| C160 | Malignant neoplasm: Cardia | 3379 |
| C161 | Malignant neoplasm: Fundus of stomach | 204 |
| C162 | Malignant neoplasm: Body of stomach | 712 |
| C163 | Malignant neoplasm: Pyloric antrum | 689 |
| C164 | Malignant neoplasm: Pylorus | 439 |
| C165 | Malignant neoplasm: Lesser curvature of stomach unspecified | 883 |
| C166 | Malignant neoplasm: Greater curvature of stomach unspecified | 358 |
| C168 | Malignant neoplasm: Overlapping lesion of stomach | 233 |
| C169 | Malignant neoplasm: Stomach unspecified | 5963 |
| C17 | Malignant neoplasm of small intestine | 0 |
| C170 | Malignant neoplasm: Duodenum | 529 |
| C171 | Malignant neoplasm: Jejunum | 86 |
| C172 | Malignant neoplasm: Ileum | 348 |
| C173 | Malignant neoplasm: Meckel diverticulum | 8 |
| C178 | Malignant neoplasm: Overlapping lesion of small intestine | 26 |
| C179 | Malignant neoplasm: Small intestine unspecified | 352 |
| C18 | Malignant neoplasm of colon | <5 |
| C18. | NA | <5 |
| C180 | Malignant neoplasm: Caecum | 6185 |
| C181 | Malignant neoplasm: Appendix | 282 |
| C182 | Malignant neoplasm: Ascending colon | 3022 |
| C183 | Malignant neoplasm: Hepatic flexure | 1250 |
| C184 | Malignant neoplasm: Transverse colon | 2018 |
| C185 | Malignant neoplasm: Splenic flexure | 926 |
| C186 | Malignant neoplasm: Descending colon | 1427 |
| C187 | Malignant neoplasm: Sigmoid colon | 9103 |
| C188 | Malignant neoplasm: Overlapping lesion of colon | 218 |
| C189 | Malignant neoplasm: Colon unspecified | 10067 |
| C18X | NA | <5 |
| C19 | Malignant neoplasm of rectosigmoid junction | 1130 |
| C19X | NA | 4124 |
| C20 | Malignant neoplasm of rectum | 3557 |
| C20+ | NA | <5 |
| C20X | NA | 11863 |
| C21 | Malignant neoplasm of anus and anal canal | 0 |
| C210 | Malignant neoplasm: Anus unspecified | 663 |
| C211 | Malignant neoplasm: Anal canal | 562 |
| C212 | Malignant neoplasm: Cloacogenic zone | 9 |
| C218 | Malignant neoplasm: Overlapping lesion of rectum anus and anal canal | 257 |
| C22 | Malignant neoplasm of liver and intrahepatic bile ducts | 0 |
| C220 | Malignant neoplasm: Liver cell carcinoma | 1741 |
| C221 | Malignant neoplasm: Intrahepatic bile duct carcinoma | 2032 |
| C222 | Malignant neoplasm: Hepatoblastoma | 13 |
| C223 | Malignant neoplasm: Angiosarcoma of liver | 16 |
| C224 | Malignant neoplasm: Other sarcomas of liver | <5 |
| C227 | Malignant neoplasm: Other specified carcinomas of liver | 30 |
| C229 | Malignant neoplasm: Liver unspecified | 1122 |
| C23 | Malignant neoplasm of gallbladder | 320 |
| C23X | NA | 580 |
| C24 | Malignant neoplasm of other and unspecified parts of biliary tract | 0 |
| C240 | Malignant neoplasm: Extrahepatic bile duct | 573 |
| C241 | Malignant neoplasm: Ampulla of Vater | 468 |
| C248 | Malignant neoplasm: Overlapping lesion of biliary tract | 12 |
| C249 | Malignant neoplasm: Biliary tract unspecified | 217 |
| C25 | Malignant neoplasm of pancreas | 0 |
| C250 | Malignant neoplasm: Head of pancreas | 3508 |
| C251 | Malignant neoplasm: Body of pancreas | 486 |
| C252 | Malignant neoplasm: Tail of pancreas | 534 |
| C253 | Malignant neoplasm: Pancreatic duct | 90 |
| C254 | Malignant neoplasm: Endocrine pancreas | 48 |
| C257 | Malignant neoplasm: Other parts of pancreas | 110 |
| C258 | Malignant neoplasm: Overlapping lesion of pancreas | 121 |
| C259 | Malignant neoplasm: Pancreas unspecified | 6642 |
| C26 | Malignant neoplasm of other and ill-defined digestive organs | 0 |
| C260 | Malignant neoplasm: Intestinal tract part unspecified | 2712 |
| C261 | Malignant neoplasm: Spleen | 31 |
| C268 | Malignant neoplasm: Overlapping lesion of digestive system | 45 |
| C269 | Malignant neoplasm: Ill-defined sites within the digestive system | 459 |
| C30 | Malignant neoplasm of nasal cavity and middle ear | 0 |
| C300 | Malignant neoplasm: Nasal cavity | 275 |
| C301 | Malignant neoplasm: Middle ear | 35 |
| C31 | Malignant neoplasm of accessory sinuses | 0 |
| C310 | Malignant neoplasm: Maxillary sinus | 140 |
| C311 | Malignant neoplasm: Ethmoidal sinus | 55 |
| C312 | Malignant neoplasm: Frontal sinus | 15 |
| C313 | Malignant neoplasm: Sphenoidal sinus | 17 |
| C318 | Malignant neoplasm: Overlapping lesion of accessory sinuses | 9 |
| C319 | Malignant neoplasm: Accessory sinus unspecified | 48 |
| C32 | Malignant neoplasm of larynx | 0 |
| C320 | Malignant neoplasm: Glottis | 1218 |
| C321 | Malignant neoplasm: Supraglottis | 707 |
| C322 | Malignant neoplasm: Subglottis | 91 |
| C323 | Malignant neoplasm: Laryngeal cartilage | 95 |
| C328 | Malignant neoplasm: Overlapping lesion of larynx | 59 |
| C329 | Malignant neoplasm: Larynx unspecified | 1224 |
| C33 | Malignant neoplasm of trachea | 16 |
| C33X | NA | 114 |
| C34 | Malignant neoplasm of bronchus and lung | 0 |
| C340 | Malignant neoplasm: Main bronchus | 3250 |
| C341 | Malignant neoplasm: Upper lobe bronchus or lung | 11960 |
| C342 | Malignant neoplasm: Middle lobe bronchus or lung | 1267 |
| C343 | Malignant neoplasm: Lower lobe bronchus or lung | 6166 |
| C348 | Malignant neoplasm: Overlapping lesion of bronchus and lung | 296 |
| C349 | Malignant neoplasm: Bronchus or lung unspecified | 32889 |
| C34X | NA | <5 |
| C37 | Malignant neoplasm of thymus | 15 |
| C37X | NA | 74 |
| C38 | Malignant neoplasm of heart mediastinum and pleura | 0 |
| C380 | Malignant neoplasm: Heart | 29 |
| C381 | Malignant neoplasm: Anterior mediastinum | 31 |
| C382 | Malignant neoplasm: Posterior mediastinum | 16 |
| C383 | Malignant neoplasm: Mediastinum part unspecified | 150 |
| C384 | Malignant neoplasm: Pleura | 403 |
| C388 | Malignant neoplasm: Overlapping lesion of heart mediastinum and pleura | 5 |
| C39 | Malignant neoplasm of other and ill-defined sites in the respiratory system and intrathoracic organs | 0 |
| C390 | Malignant neoplasm: Upper respiratory tract part unspecified | 6 |
| C398 | Malignant neoplasm: Overlapping lesion of respiratory and intrathoracic organs | 11 |
| C399 | Malignant neoplasm: Ill-defined sites within the respiratory system | 10 |
| C40 | Malignant neoplasm of bone and articular cartilage of limbs | 0 |
| C400 | Malignant neoplasm: Scapula and long bones of upper limb | 45 |
| C401 | Malignant neoplasm: Short bones of upper limb | 6 |
| C402 | Malignant neoplasm: Long bones of lower limb | 120 |
| C403 | Malignant neoplasm: Short bones of lower limb | 12 |
| C408 | Malignant neoplasm: Overlapping lesion of bone and articular cartilage of limbs | 5 |
| C409 | Malignant neoplasm: Bone and articular cartilage of limb unspecified | 20 |
| C41 | Malignant neoplasm of bone and articular cartilage of other and unspecified sites | 0 |
| C410 | Malignant neoplasm: Bones of skull and face | 154 |
| C411 | Malignant neoplasm: Mandible | 208 |
| C412 | Malignant neoplasm: Vertebral column | 124 |
| C413 | Malignant neoplasm: Ribs sternum and clavicle | 44 |
| C414 | Malignant neoplasm: Pelvic bones sacrum and coccyx | 113 |
| C418 | Malignant neoplasm: Overlapping lesion of bone and articular cartilage | <5 |
| C419 | Malignant neoplasm: Bone and articular cartilage unspecified | 168 |
| C43 | Malignant melanoma of skin | 0 |
| C430 | Malignant neoplasm: Malignant melanoma of lip | 23 |
| C431 | Malignant neoplasm: Malignant melanoma of eyelid including canthus | 173 |
| C432 | Malignant neoplasm: Malignant melanoma of ear and external auricular canal | 175 |
| C433 | Malignant neoplasm: Malignant melanoma of other and unspecified parts of face | 891 |
| C434 | Malignant neoplasm: Malignant melanoma of scalp and neck | 425 |
| C435 | Malignant neoplasm: Malignant melanoma of trunk | 1491 |
| C436 | Malignant neoplasm: Malignant melanoma of upper limb including shoulder | 1134 |
| C437 | Malignant neoplasm: Malignant melanoma of lower limb including hip | 1493 |
| C438 | Malignant neoplasm: Overlapping malignant melanoma of skin | 7 |
| C439 | Malignant neoplasm: Malignant melanoma of skin unspecified | 1692 |
| C44 | Other malignant neoplasms of skin | 7 |
| C44. | NA | <5 |
| C440 | Malignant neoplasm: Skin of lip | 1708 |
| C441 | Malignant neoplasm: Skin of eyelid including canthus | 6473 |
| C442 | Malignant neoplasm: Skin of ear and external auricular canal | 6712 |
| C443 | Malignant neoplasm: Skin of other and unspecified parts of face | 28071 |
| C444 | Malignant neoplasm: Skin of scalp and neck | 7111 |
| C445 | Malignant neoplasm: Skin of trunk | 5706 |
| C446 | Malignant neoplasm: Skin of upper limb including shoulder | 4642 |
| C447 | Malignant neoplasm: Skin of lower limb including hip | 5427 |
| C448 | Malignant neoplasm: Overlapping lesion of skin | 23 |
| C449 | Malignant neoplasm: Malignant neoplasm of skin unspecified | 1494 |
| C45 | Mesothelioma | 0 |
| C450 | Mesothelioma of pleura | 1188 |
| C451 | Mesothelioma of peritoneum | 96 |
| C452 | Mesothelioma of pericardium | 6 |
| C457 | Mesothelioma of other sites | 240 |
| C459 | Mesothelioma unspecified | 1152 |
| C46 | Kaposi sarcoma | 0 |
| C460 | Kaposi sarcoma of skin | 12 |
| C461 | Kaposi sarcoma of soft tissue | <5 |
| C462 | Kaposi sarcoma of palate | 0 |
| C463 | Kaposi sarcoma of lymph nodes | 0 |
| C467 | Kaposi sarcoma of other sites | <5 |
| C468 | Kaposi sarcoma of multiple organs | <5 |
| C469 | Kaposi sarcoma unspecified | 16 |
| C47 | Malignant neoplasm of peripheral nerves and autonomic nervous system | 0 |
| C470 | Malignant neoplasm: Peripheral nerves of head face and neck | 14 |
| C471 | Malignant neoplasm: Peripheral nerves of upper limb including shoulder | 10 |
| C472 | Malignant neoplasm: Peripheral nerves of lower limb including hip | 13 |
| C473 | Malignant neoplasm: Peripheral nerves of thorax | 12 |
| C474 | Malignant neoplasm: Peripheral nerves of abdomen | 24 |
| C475 | Malignant neoplasm: Peripheral nerves of pelvis | 11 |
| C476 | Malignant neoplasm: Peripheral nerves of trunk unspecified | 7 |
| C478 | Malignant neoplasm: Overlapping lesion of peripheral nerves and autonomic nervous system | 0 |
| C479 | Malignant neoplasm: Peripheral nerves and autonomic nervous system unspecified | 24 |
| C47X | NA | <5 |
| C48 | Malignant neoplasm of retroperitoneum and peritoneum | 0 |
| C480 | Malignant neoplasm: Retroperitoneum | 216 |
| C481 | Malignant neoplasm: Specified parts of peritoneum | 233 |
| C482 | Malignant neoplasm: Peritoneum unspecified | 336 |
| C488 | Malignant neoplasm: Overlapping lesion of retroperitoneum and peritoneum | 7 |
| C489 | NA | <5 |
| C49 | Malignant neoplasm of other connective and soft tissue | 0 |
| C490 | Malignant neoplasm: Connective and soft tissue of head face and neck | 365 |
| C491 | Malignant neoplasm: Connective and soft tissue of upper limb including shoulder | 201 |
| C492 | Malignant neoplasm: Connective and soft tissue of lower limb including hip | 443 |
| C493 | Malignant neoplasm: Connective and soft tissue of thorax | 145 |
| C494 | Malignant neoplasm: Connective and soft tissue of abdomen | 173 |
| C495 | Malignant neoplasm: Connective and soft tissue of pelvis | 189 |
| C496 | Malignant neoplasm: Connective and soft tissue of trunk unspecified | 71 |
| C498 | Malignant neoplasm: Overlapping lesion of connective and soft tissue | 7 |
| C499 | Malignant neoplasm: Connective and soft tissue unspecified | 440 |
| C50 | Malignant neoplasm of breast | <5 |
| C500 | Malignant neoplasm: Nipple and areola | 1048 |
| C501 | Malignant neoplasm: Central portion of breast | 2216 |
| C502 | Malignant neoplasm: Upper-inner quadrant of breast | 2852 |
| C503 | Malignant neoplasm: Lower-inner quadrant of breast | 1523 |
| C504 | Malignant neoplasm: Upper-outer quadrant of breast | 9449 |
| C505 | Malignant neoplasm: Lower-outer quadrant of breast | 1862 |
| C506 | Malignant neoplasm: Axillary tail of breast | 324 |
| C508 | Malignant neoplasm: Overlapping lesion of breast | 2296 |
| C509 | Malignant neoplasm: Breast unspecified | 26791 |
| C50X | NA | <5 |
| C51 | Malignant neoplasm of vulva | 0 |
| C510 | Malignant neoplasm: Labium majus | 64 |
| C511 | Malignant neoplasm: Labium minus | 49 |
| C512 | Malignant neoplasm: Clitoris | 25 |
| C518 | Malignant neoplasm: Overlapping lesion of vulva | 27 |
| C519 | Malignant neoplasm: Vulva unspecified | 1108 |
| C52 | Malignant neoplasm of vagina | 104 |
| C52X | NA | 355 |
| C53 | Malignant neoplasm of cervix uteri | <5 |
| C530 | Malignant neoplasm: Endocervix | 234 |
| C531 | Malignant neoplasm: Exocervix | 18 |
| C538 | Malignant neoplasm: Overlapping lesion of cervix uteri | 36 |
| C539 | Malignant neoplasm: Cervix uteri unspecified | 1776 |
| C53X | NA | <5 |
| C54 | Malignant neoplasm of corpus uteri | 0 |
| C540 | Malignant neoplasm: Isthmus uteri | 21 |
| C541 | Malignant neoplasm: Endometrium | 6434 |
| C542 | Malignant neoplasm: Myometrium | 170 |
| C543 | Malignant neoplasm: Fundus uteri | 21 |
| C548 | Malignant neoplasm: Overlapping lesion of corpus uteri | 60 |
| C549 | Malignant neoplasm: Corpus uteri unspecified | 381 |
| C54X | NA | <5 |
| C55 | Malignant neoplasm of uterus part unspecified | 431 |
| C55X | NA | 836 |
| C56 | Malignant neoplasm of ovary | 2938 |
| C56+ | NA | <5 |
| C56X | NA | 6114 |
| C57 | Malignant neoplasm of other and unspecified female genital organs | 0 |
| C570 | Malignant neoplasm: Fallopian tube | 167 |
| C571 | Malignant neoplasm: Broad ligament | 5 |
| C572 | Malignant neoplasm: Round ligament | <5 |
| C573 | Malignant neoplasm: Parametrium | <5 |
| C574 | Malignant neoplasm: Uterine adnexa unspecified | 18 |
| C577 | Malignant neoplasm: Other specified female genital organs | 9 |
| C578 | Malignant neoplasm: Overlapping lesion of female genital organs | 73 |
| C579 | Malignant neoplasm: Female genital organ unspecified | 156 |
| C58 | Malignant neoplasm of placenta | <5 |
| C60 | Malignant neoplasm of penis | 0 |
| C600 | Malignant neoplasm: Prepuce | 70 |
| C601 | Malignant neoplasm: Glans penis | 218 |
| C602 | Malignant neoplasm: Body of penis | 24 |
| C608 | Malignant neoplasm: Overlapping lesion of penis | 19 |
| C609 | Malignant neoplasm: Penis unspecified | 309 |
| C60X | NA | 7 |
| C61 | Malignant neoplasm of prostate | 10418 |
| C61+ | NA | 5 |
| C619 | NA | <5 |
| C61X | NA | 35972 |
| C62 | Malignant neoplasm of testis | 0 |
| C620 | Malignant neoplasm: Undescended testis | 10 |
| C621 | Malignant neoplasm: Descended testis | 69 |
| C629 | Malignant neoplasm: Testis unspecified | 430 |
| C63 | Malignant neoplasm of other and unspecified male genital organs | 0 |
| C630 | Malignant neoplasm: Epididymis | <5 |
| C631 | Malignant neoplasm: Spermatic cord | 11 |
| C632 | Malignant neoplasm: Scrotum | 64 |
| C637 | Malignant neoplasm: Other specified male genital organs | <5 |
| C638 | Malignant neoplasm: Overlapping lesion of male genital organs | <5 |
| C639 | Malignant neoplasm: Male genital organ unspecified | 7 |
| C64 | Malignant neoplasm of kidney except renal pelvis | 2893 |
| C64L | NA | <5 |
| C64R | NA | <5 |
| C64X | NA | 6591 |
| C65 | Malignant neoplasm of renal pelvis | 33 |
| C65X | NA | 539 |
| C66 | Malignant neoplasm of ureter | 122 |
| C668 | NA | <5 |
| C66X | NA | 772 |
| C67 | Malignant neoplasm of bladder | <5 |
| C67. | NA | <5 |
| C670 | Malignant neoplasm: Trigone of bladder | 528 |
| C671 | Malignant neoplasm: Dome of bladder | 1074 |
| C672 | Malignant neoplasm: Lateral wall of bladder | 2881 |
| C673 | Malignant neoplasm: Anterior wall of bladder | 944 |
| C674 | Malignant neoplasm: Posterior wall of bladder | 1789 |
| C675 | Malignant neoplasm: Bladder neck | 1254 |
| C676 | Malignant neoplasm: Ureteric orifice | 1716 |
| C677 | Malignant neoplasm: Urachus | 15 |
| C678 | Malignant neoplasm: Overlapping lesion of bladder | 609 |
| C679 | Malignant neoplasm: Bladder unspecified | 18025 |
| C67X | NA | <5 |
| C68 | Malignant neoplasm of other and unspecified urinary organs | 0 |
| C680 | Malignant neoplasm: Urethra | 265 |
| C681 | Malignant neoplasm: Paraurethral gland | 0 |
| C688 | Malignant neoplasm: Overlapping lesion of urinary organs | 22 |
| C689 | Malignant neoplasm: Urinary organ unspecified | 172 |
| C69 | Malignant neoplasm of eye and adnexa | 0 |
| C690 | Malignant neoplasm: Conjunctiva | 65 |
| C691 | Malignant neoplasm: Cornea | 7 |
| C692 | Malignant neoplasm: Retina | 24 |
| C693 | Malignant neoplasm: Choroid | 404 |
| C694 | Malignant neoplasm: Ciliary body | 66 |
| C695 | Malignant neoplasm: Lacrimal gland and duct | 10 |
| C696 | Malignant neoplasm: Orbit | 89 |
| C698 | Malignant neoplasm: Overlapping lesion of eye and adnexa | 8 |
| C699 | Malignant neoplasm: Eye unspecified | 134 |
| C70 | Malignant neoplasm of meninges | 0 |
| C700 | Malignant neoplasm: Cerebral meninges | 100 |
| C701 | Malignant neoplasm: Spinal meninges | 13 |
| C709 | Malignant neoplasm: Meninges unspecified | 61 |
| C71 | Malignant neoplasm of brain | 0 |
| C71. | NA | <5 |
| C710 | Malignant neoplasm: Cerebrum except lobes and ventricles | 356 |
| C711 | Malignant neoplasm: Frontal lobe | 824 |
| C712 | Malignant neoplasm: Temporal lobe | 624 |
| C713 | Malignant neoplasm: Parietal lobe | 759 |
| C714 | Malignant neoplasm: Occipital lobe | 175 |
| C715 | Malignant neoplasm: Cerebral ventricle | 40 |
| C716 | Malignant neoplasm: Cerebellum | 115 |
| C717 | Malignant neoplasm: Brain stem | 50 |
| C718 | Malignant neoplasm: Overlapping lesion of brain | 376 |
| C719 | Malignant neoplasm: Brain unspecified | 2772 |
| C72 | Malignant neoplasm of spinal cord cranial nerves and other parts of central nervous system | 0 |
| C720 | Malignant neoplasm: Spinal cord | 85 |
| C721 | Malignant neoplasm: Cauda equina | 8 |
| C722 | Malignant neoplasm: Olfactory nerve | 7 |
| C723 | Malignant neoplasm: Optic nerve | 9 |
| C724 | Malignant neoplasm: Acoustic nerve | 9 |
| C725 | Malignant neoplasm: Other and unspecified cranial nerves | 9 |
| C728 | Malignant neoplasm: Overlapping lesion of brain and other parts of central nervous system | 6 |
| C729 | Malignant neoplasm: Central nervous system unspecified | 11 |
| C72X | NA | <5 |
| C73 | Malignant neoplasm of thyroid gland | 241 |
| C73X | NA | 899 |
| C74 | Malignant neoplasm of adrenal gland | 0 |
| C740 | Malignant neoplasm: Cortex of adrenal gland | 44 |
| C741 | Malignant neoplasm: Medulla of adrenal gland | 18 |
| C749 | Malignant neoplasm: Adrenal gland unspecified | 193 |
| C75 | Malignant neoplasm of other endocrine glands and related structures | 0 |
| C750 | Malignant neoplasm: Parathyroid gland | 20 |
| C751 | Malignant neoplasm: Pituitary gland | 95 |
| C752 | Malignant neoplasm: Craniopharyngeal duct | 5 |
| C753 | Malignant neoplasm: Pineal gland | 23 |
| C754 | Malignant neoplasm: Carotid body | 6 |
| C755 | Malignant neoplasm: Aortic body and other paraganglia | 18 |
| C758 | Malignant neoplasm: Pluriglandular involvement unspecified | <5 |
| C759 | Malignant neoplasm: Endocrine gland unspecified | 46 |
| C75X | NA | <5 |
| C76 | Malignant neoplasm of other and ill-defined sites | 0 |
| C760 | Malignant neoplasm of other and ill-defined sites: Head face and neck | 421 |
| C761 | Malignant neoplasm of other and ill-defined sites: Thorax | 173 |
| C762 | Malignant neoplasm of other and ill-defined sites: Abdomen | 453 |
| C763 | Malignant neoplasm of other and ill-defined sites: Pelvis | 384 |
| C764 | Malignant neoplasm of other and ill-defined sites: Upper limb | 65 |
| C765 | Malignant neoplasm of other and ill-defined sites: Lower limb | 83 |
| C767 | Malignant neoplasm of other and ill-defined sites: Other ill-defined sites | 45 |
| C768 | Malignant neoplasm of other and ill-defined sites: Overlapping lesion of other and ill-defined sites | 27 |
| C77 | Secondary and unspecified malignant neoplasm of lymph nodes | 0 |
| C770 | Secondary and unspecified malignant neoplasm: Lymph nodes of head face and neck | 6138 |
| C771 | Secondary and unspecified malignant neoplasm: Intrathoracic lymph nodes | 7878 |
| C772 | Secondary and unspecified malignant neoplasm: Intra-abdominal lymph nodes | 12351 |
| C773 | Secondary and unspecified malignant neoplasm: Axillary and upper limb lymph nodes | 10594 |
| C774 | Secondary and unspecified malignant neoplasm: Inguinal and lower limb lymph nodes | 1697 |
| C775 | Secondary and unspecified malignant neoplasm: Intrapelvic lymph nodes | 3479 |
| C778 | Secondary and unspecified malignant neoplasm: Lymph nodes of multiple regions | 461 |
| C779 | Secondary and unspecified malignant neoplasm: Lymph node unspecified | 3133 |
| C77X | NA | <5 |
| C78 | Secondary malignant neoplasm of respiratory and digestive organs | <5 |
| C780 | Secondary malignant neoplasm of lung | 20044 |
| C781 | Secondary malignant neoplasm of mediastinum | 841 |
| C782 | Secondary malignant neoplasm of pleura | 4604 |
| C783 | Secondary malignant neoplasm of other and unspecified respiratory organs | 180 |
| C784 | Secondary malignant neoplasm of small intestine | 665 |
| C785 | Secondary malignant neoplasm of large intestine and rectum | 1374 |
| C786 | Secondary malignant neoplasm of retroperitoneum and peritoneum | 11163 |
| C787 | Secondary malignant neoplasm of liver and intrahepatic bile duct | 33322 |
| C788 | Secondary malignant neoplasm of other and unspecified digestive organs | 2092 |
| C789 | NA | <5 |
| C78X | NA | <5 |
| C79 | Secondary malignant neoplasm of other and unspecified sites | <5 |
| C790 | Secondary malignant neoplasm of kidney and renal pelvis | 819 |
| C791 | Secondary malignant neoplasm of bladder and other and unspecified urinary organs | 581 |
| C792 | Secondary malignant neoplasm of skin | 2257 |
| C793 | Secondary malignant neoplasm of brain and cerebral meninges | 8726 |
| C794 | Secondary malignant neoplasm of other and unspecified parts of nervous system | 355 |
| C795 | Secondary malignant neoplasm of bone and bone marrow | 26751 |
| C796 | Secondary malignant neoplasm of ovary | 681 |
| C797 | Secondary malignant neoplasm of adrenal gland | 3509 |
| C798 | Secondary malignant neoplasm of other specified sites | 9974 |
| C799 | Secondary malignant neoplasm unspecified site | 1105 |
| C79D | NA | <5 |
| C80 | Malignant neoplasm without specification of site | 24053 |
| C80- | NA | <5 |
| C80+ | NA | <5 |
| C800 | Malignant neoplasm primary site unknown so stated | 3279 |
| C809 | Malignant neoplasm primary site unspecified | 2717 |
| C80D | NA | <5 |
| C80X | NA | 13445 |
| C81 | Hodgkin lymphoma | 0 |
| C810 | Nodular lymphocyte predominant Hodgkin lymphoma | 80 |
| C811 | Nodular sclerosis (classical) Hodgkin lymphoma | 451 |
| C812 | Mixed cellularity (classical) Hodgkin lymphoma | 161 |
| C813 | Lymphocyte depleted (classical) Hodgkin lymphoma | 24 |
| C814 | Lymphocyte-rich (classical) Hodgkin lymphoma | 5 |
| C817 | Other (classical) Hodgkin lymphoma | 43 |
| C819 | Hodgkin lymphoma unspecified | 971 |
| C82 | Follicular lymphoma | 0 |
| C820 | Follicular lymphoma grade I | 108 |
| C821 | Follicular lymphoma grade II | 166 |
| C822 | Follicular lymphoma grade III unspecified | 115 |
| C823 | Follicular lymphoma grade IIIa | 85 |
| C824 | Follicular lymphoma grade IIIb | 33 |
| C825 | Diffuse follicle centre lymphoma | <5 |
| C826 | Cutaneous follicle centre lymphoma | 9 |
| C827 | Other types of follicular lymphoma | 227 |
| C829 | Follicular lymphoma unspecified | 1288 |
| C83 | Non-follicular lymphoma | 0 |
| C830 | Small cell B-cell lymphoma | 455 |
| C831 | Mantle cell lymphoma | 314 |
| C832 | NA | 9 |
| C833 | Diffuse large B-cell lymphoma | 2921 |
| C834 | NA | 11 |
| C835 | Lymphoblastic (diffuse) lymphoma | 54 |
| C836 | NA | 8 |
| C837 | Burkitt lymphoma | 103 |
| C838 | Other non-follicular lymphoma | 684 |
| C839 | Non-follicular (diffuse) lymphoma unspecified | 225 |
| C84 | Mature T/NK-cell lymphomas | 0 |
| C840 | Mycosis fungoides | 184 |
| C841 | SÃ©zary disease | 34 |
| C842 | NA | <5 |
| C843 | NA | <5 |
| C844 | Peripheral T-cell lymphoma not elsewhere classified | 269 |
| C845 | Other mature T/NK-cell lymphomas | 339 |
| C846 | Anaplastic large cell lymphoma ALK-positive | 6 |
| C847 | Anaplastic large cell lymphoma ALK-negative | 26 |
| C848 | Cutaneous T-cell lymphoma unspecified | 41 |
| C849 | Mature T/NK-cell lymphoma unspecified | <5 |
| C85 | Other and unspecified types of non-Hodgkin lymphoma | 0 |
| C85. | NA | <5 |
| C850 | NA | 14 |
| C851 | B-cell lymphoma unspecified | 2160 |
| C852 | Mediastinal (thymic) large B-cell lymphoma | 27 |
| C857 | Other specified types of non-Hodgkin lymphoma | 368 |
| C859 | Non-Hodgkin lymphoma unspecified | 6367 |
| C86 | Other specified types of T/NK-cell lymphoma | 0 |
| C860 | Extranodal NK/T-cell lymphoma nasal type | 5 |
| C861 | Hepatosplenic T-cell lymphoma | <5 |
| C862 | Enteropathy-type (intestinal) T-cell lymphoma | 6 |
| C863 | Subcutaneous panniculitis-like T-cell lymphoma | 0 |
| C864 | Blastic NK-cell lymphoma | 5 |
| C865 | Angioimmunoblastic T-cell lymphoma | 42 |
| C866 | Primary cutaneous CD30-positive T-cell proliferations | 14 |
| C88 | Malignant immunoproliferative diseases | 0 |
| C880 | WaldenstrÃ¶m macroglobulinaemia | 500 |
| C881 | NA | <5 |
| C882 | Other heavy chain disease | 0 |
| C883 | Immunoproliferative small intestinal disease | <5 |
| C884 | Extranodal marginal zone B-cell lymphoma of mucosa-associated lymphoid tissue [MALT-lymphoma] | 207 |
| C887 | Other malignant immunoproliferative diseases | <5 |
| C889 | Malignant immunoproliferative disease unspecified | 10 |
| C90 | Multiple myeloma and malignant plasma cell neoplasms | <5 |
| C900 | Multiple myeloma | 5375 |
| C901 | Plasma cell leukaemia | 87 |
| C902 | Extramedullary plasmacytoma | 281 |
| C903 | Solitary plasmacytoma | 97 |
| C90X | NA | <5 |
| C91 | Lymphoid leukaemia | 0 |
| C910 | Acute lymphoblastic leukaemia [ALL] | 1175 |
| C911 | Chronic lymphocytic leukaemia of B-cell type | 4689 |
| C912 | NA | 5 |
| C913 | Prolymphocytic leukaemia of B-cell type | 63 |
| C914 | Hairy-cell leukaemia | 225 |
| C915 | Adult T-cell lymphoma/leukaemia [HTLV-1-associated] | 63 |
| C916 | Prolymphocytic leukaemia of T-cell type | 12 |
| C917 | Other lymphoid leukaemia | 26 |
| C918 | Mature B-cell leukaemia Burkitt-type | 0 |
| C919 | Lymphoid leukaemia unspecified | 237 |
| C91X | NA | <5 |
| C92 | Myeloid leukaemia | 0 |
| C920 | Acute myeloblastic leukaemia [AML] | 3321 |
| C921 | Chronic myeloid leukaemia [CML] BCR/ABL-positive | 933 |
| C922 | Atypical chronic myeloid leukaemia BCR/ABL-negative | 7 |
| C923 | Myeloid sarcoma | 13 |
| C924 | Acute promyelocytic leukaemia [PML] | 95 |
| C925 | Acute myelomonocytic leukaemia | 88 |
| C926 | Acute myeloid leukaemia with 11q23-abnormality | <5 |
| C927 | Other myeloid leukaemia | 331 |
| C928 | Acute myeloid leukaemia with multilineage dysplasia | 15 |
| C929 | Myeloid leukaemia unspecified | 111 |
| C92X | NA | <5 |
| C93 | Monocytic leukaemia | 0 |
| C930 | Acute monoblastic/monocytic leukaemia | 97 |
| C931 | Chronic myelomonocytic leukaemia | 234 |
| C933 | Juvenile myelomonocytic leukaemia | 0 |
| C937 | Other monocytic leukaemia | <5 |
| C939 | Monocytic leukaemia unspecified | 6 |
| C94 | Other leukaemias of specified cell type | 0 |
| C940 | Acute erythroid leukaemia | 23 |
| C941 | NA | 10 |
| C942 | Acute megakaryoblastic leukaemia | 13 |
| C943 | Mast cell leukaemia | <5 |
| C944 | Acute panmyelosis with myelofibrosis | 48 |
| C945 | NA | 80 |
| C946 | Myelodysplastic and myeloproliferative disease not elsewhere classified | 77 |
| C947 | Other specified leukaemias | 0 |
| C95 | Leukaemia of unspecified cell type | 0 |
| C950 | Acute leukaemia of unspecified cell type | 211 |
| C951 | Chronic leukaemia of unspecified cell type | 81 |
| C957 | Other leukaemia of unspecified cell type | 8 |
| C959 | Leukaemia unspecified | 381 |
| C96 | Other and unspecified malignant neoplasms of lymphoid haematopoietic and related tissue | 0 |
| C960 | Multifocal and multisystemic (disseminated) Langerhans-cell histiocytosis [Letterer-Siwe disease] | 5 |
| C961 | NA | 8 |
| C962 | Malignant mast cell tumour | 17 |
| C964 | Sarcoma of dendritic cells (accessory cells) | <5 |
| C965 | Multifocal and unisystemic Langerhans-cell histiocytosis | <5 |
| C966 | Unifocal Langerhans-cell histiocytosis | 8 |
| C967 | Other specified malignant neoplasms of lymphoid haematopoietic and related tissue | 8 |
| C968 | Histiocytic sarcoma | <5 |
| C969 | Malignant neoplasm of lymphoid haematopoietic and related tissue unspecified | 75 |
| C97 | Malignant neoplasms of independent (primary) multiple sites | 604 |
| C97X | NA | 1401 |

## Descriptives

327654 people had at least one diagnostic code in at least one of the datasets. 283635 people had a code in hospital admissions data, 135230 in mortality data and 180764 in primary care data. The following figure shows the year of the first code that was found for any person classified positive using (a) all codes combined, (b) only codes from hospital admissions data, (c) only codes from the mortality data and (d) only codes from primary care data.
