# Supplementary material for: The Secure Anonymised Information Linkage databank Dementia e-cohort (SAIL-DeC)
Source: Int J Popul Data Sci. 2020 Feb 25;5(1):1121. doi: 10.23889/ijpds.v5i1.1121 (PMC7473277; doi:10.23889/ijpds.v5i1.1121)
Supplement: Supplementary Material [file ijpds-05-01-1121-s001.zip › Supplementary Appendix 13.html]

Event tables


# Event tables

### *COPD*

#### *Christian*

#### *January 2019*

## Code selection

We have selected codes based on UK Biobank algorithm and COPD validation study (unpublished) in conjunction with the WHO ICD 10 browser (apps.who.int/classifications/icd10/browse/2010/en) and the NHS Read Code Browser (https://isd.digital.nhs.uk/trud3/user/guest/group/0/home). We have deliberately included codes with obvious `misspelling’ (for example having a dot where none should be) or ICD 10 codes ending with ‘X’.

All codes that were selected for classification and the total number of people with at least one of the codes are displayed in the following tables. Please be aware that frequency counts of Read V2 codes in the table do not reflect the hierarchical nature of Read V2 coding (for example, counts of E01.. do not include E011.).

### Read V2 codes:

| code | desc | total\_n |
| --- | --- | --- |
| 663K. | Airways obstructn irreversible | 14392 |
| 66YB. | Chronic obstructive pulmonary disease monitoring | 44609 |
| 66YB0 | Chronic obstructive pulmonary disease 3 monthly review | 4378 |
| 66YB1 | Chronic obstructive pulmonary disease 6 monthly review | 5052 |
| 66YB2 | Telehealth chronic obstructive pulmonary disease monitoring | 0 |
| 66YD. | Chronic obstructive pulmonary disease monitoring due | 857 |
| 66Yg. | Chronic obstructive pulmonary disease disturbs sleep | 1660 |
| 66Yh. | Chronic obstructive pulmonary disease does not disturb sleep | 5668 |
| 66YL. | Chronic obstructive pulmonary disease follow-up | 18993 |
| 66YM. | Chronic obstructive pulmonary disease annual review | 83949 |
| 66YS. | Chronic obstructive pulmonary disease monitoring by nurse | 1022 |
| 66YT. | Chronic obstructive pulmonary disease monitoring by doctor | 342 |
| 679V. | Health education - chronic obstructive pulmonary disease | 8492 |
| 8CR1. | Chronic obstructive pulmonary disease clinical management plan | 2716 |
| 9Oi.. | Chronic obstructive pulmonary disease monitoring administration | 4325 |
| 9Oi0. | Chronic obstructive pulmonary disease monitoring 1st letter | 50236 |
| 9Oi1. | Chronic obstructive pulmonary disease monitoring 2nd letter | 23456 |
| 9Oi2. | Chronic obstructive pulmonary disease monitoring 3rd letter | 12379 |
| 9Oi3. | Chronic obstructive pulmonary disease monitoring verbal invite | 4047 |
| 9Oi4. | Chronic obstructive pulmonary disease monitoring phone invite | 6755 |
| H3… | Chronic obstructive pulmonary disease | 96936 |
| H3121 | Emphysematous bronchitis | 403 |
| H32.. | Emphysema | 13933 |
| H320. | Chronic bullous emphysema | 180 |
| H3200 | Segmental bullous emphysema | 26 |
| H3201 | Zonal bullous emphysema | 6 |
| H3202 | Giant bullous emphysema | 0 |
| H3203 | Bullous emphysema with collapse | 11 |
| H320z | Chronic bullous emphysema NOS | 20 |
| H322. | Centrilobular emphysema | 169 |
| H32z. | Emphysema NOS | 481 |
| H36.. | Mild chronic obstructive pulmonary disease | 18414 |
| H37.. | Moderate chronic obstructive pulmonary disease | 19427 |
| H38.. | Severe chronic obstructive pulmonary disease | 9274 |
| H39.. | Very severe chronic obstructive pulmonary disease | 1171 |
| H3y.. | Other specified chronic obstructive airways disease | 527 |
| H3y0. | Chronic obstructive pulmonary disease with acute lower respiratory infection | 812 |
| H3y1. | Chronic obstructive pulmonary disease with acute exacerbation, unspecified | 11504 |
| H3z.. | Chronic obstructive airways disease NOS | 15620 |

### ICD 9 and 10 codes:

| code | desc | total\_n |
| --- | --- | --- |
| 4912 | Obstructive chronic bronchitis | 278 |
| 492 | Emphysema | 949 |
| J43 | Emphysema | <5 |
| J430 | MacLeod syndrome | 35 |
| J431 | Panlobular emphysema | 92 |
| J432 | Centrilobular emphysema | 1218 |
| J438 | Other emphysema | 203 |
| J439 | Emphysema unspecified | 29927 |
| J43X | NA | 23 |
| J44 | Other chronic obstructive pulmonary disease | 13 |
| J440 | Chronic obstructive pulmonary disease with acute lower respiratory infection | 61301 |
| J441 | Chronic obstructive pulmonary disease with acute exacerbation unspecified | 35243 |
| J448 | Other specified chronic obstructive pulmonary disease | 7725 |
| J449 | Chronic obstructive pulmonary disease unspecified | 126863 |
| J44X | NA | 209 |

## Descriptives

200571 people had at least one diagnostic code in at least one of the datasets. 151466 people had a code in hospital admissions data, 38715 in mortality data and 133142 in primary care data. The following figure shows the year of the first code that was found for any person classified positive using (a) all codes combined, (b) only codes from hospital admissions data, (c) only codes from the mortality data and (d) only codes from primary care data.
