# Supplementary material for: The Secure Anonymised Information Linkage databank Dementia e-cohort (SAIL-DeC)
Source: Int J Popul Data Sci. 2020 Feb 25;5(1):1121. doi: 10.23889/ijpds.v5i1.1121 (PMC7473277; doi:10.23889/ijpds.v5i1.1121)
Supplement: Supplementary Material [file ijpds-05-01-1121-s001.zip › Supplementary Appendix 14.html]

Event tables


# Event tables

### *Depression*

#### *Christian*

#### *January 2019*

## Code selection

We have selected codes based on Moran, C et al. Incidence, clinical management, and mortality risk following self-harm among children and adolescents: cohort study in primary care. BMJ 2017; 359:j45351. doi: doi.org/10.1136/bmj.j4351 in conjunction with the WHO ICD 10 browser (apps.who.int/classifications/icd10/browse/2010/en) and the NHS Read Code Browser (https://isd.digital.nhs.uk/trud3/user/guest/group/0/home). We have deliberately included codes with obvious `misspelling’ (for example having a dot where none should be) or ICD 10 codes ending with ‘X’.

All codes that were selected for classification and the total number of people with at least one of the codes are displayed in the following tables. Please be aware that frequency counts of Read V2 codes in the table do not reflect the hierarchical nature of Read V2 coding (for example, counts of E01.. do not include E011.).

### Read V2 codes:

| code | desc | total\_n |
| --- | --- | --- |
| 1B17. | Depressed | 64820 |
| 2257. | O/E - depressed | 12044 |
| 62T1. | Puerperal depression | 431 |
| 9kQ.. | On full dose long term treatment for depression - enhanced services administration | 12 |
| E0013 | Presenile dementia with depression | 48 |
| E0021 | Senile dementia with depression | 259 |
| E0043 | Arteriosclerotic dementia with depression | 32 |
| E112. | Single major depressive episode | 20019 |
| E1120 | Single major depressive episode, unspecified | 450 |
| E1121 | Single major depressive episode, mild | 516 |
| E1122 | Single major depressive episode, moderate | 555 |
| E1123 | Single major depressive episode, severe, without mention of psychosis | 208 |
| E1124 | Single major depressive episode, severe, with psychosis | 74 |
| E1125 | Single major depressive episode, in partial or unspecified remission | 24 |
| E1126 | Single major depressive episode, in full remission | 20 |
| E112z | Single major depressive episode NOS | 156 |
| E113. | Recurrent major depressive episode | 7975 |
| E1130 | Recurrent major depressive episodes, unspecified | 224 |
| E1131 | Recurrent major depressive episodes, mild | 280 |
| E1132 | Recurrent major depressive episodes, moderate | 449 |
| E1133 | Recurrent major depressive episodes, severe, without mention of psychosis | 132 |
| E1134 | Recurrent major depressive episodes, severe, with psychosis | 124 |
| E1135 | Recurrent major depressive episodes, in partial or unspecified remission | 41 |
| E1136 | Recurrent major depressive episodes, in full remission | 23 |
| E1137 | Recurrent depression | 5089 |
| E113z | Recurrent major depressive episode NOS | 164 |
| E118. | Seasonal affective disorder | 370 |
| E11y2 | Atypical depressive disorder | 42 |
| E11z2 | Masked depression | 751 |
| E130. | Reactive depressive psychosis | 815 |
| E135. | Agitated depression | 3861 |
| E2003 | Anxiety with depression | 72888 |
| E204. | Neurotic depression reactive type | 24122 |
| E290. | Brief depressive reaction | 580 |
| E290z | Brief depressive reaction NOS | 63 |
| E291. | Prolonged depressive reaction | 378 |
| E2B.. | Depressive disorder NEC | 56836 |
| E2B0. | Postviral depression | 132 |
| E2B1. | Chronic depression | 6899 |
| Eu204 | [X]Post-schizophrenic depression | 5 |
| Eu32. | [X]Depressive episode | 21820 |
| Eu320 | [X]Mild depressive episode | 3169 |
| Eu321 | [X]Moderate depressive episode | 4206 |
| Eu322 | [X]Severe depressive episode without psychotic symptoms | 1324 |
| Eu323 | [X]Severe depressive episode with psychotic symptoms | 680 |
| Eu324 | [X]Mild depression | 1744 |
| Eu325 | [X]Major depression, mild | 50 |
| Eu326 | [X]Major depression, moderately severe | 101 |
| Eu327 | [X]Major depression, severe without psychotic symptoms | 67 |
| Eu328 | [X]Major depression, severe with psychotic symptoms | 74 |
| Eu329 | [X]Single major depressive episode, severe, with psychosis, psychosis in remission | 115 |
| Eu32A | [X]Recurrent major depressive episodes, severe, with psychosis, psychosis in remission | 61 |
| Eu32B | [X]Antenatal depression | 0 |
| Eu32y | [X]Other depressive episodes | 740 |
| Eu32z | [X]Depressive episode, unspecified | 56474 |
| Eu33. | [X]Recurrent depressive disorder | 4151 |
| Eu330 | [X]Recurrent depressive disorder, current episode mild | 211 |
| Eu331 | [X]Recurrent depressive disorder, current episode moderate | 221 |
| Eu332 | [X]Recurrent depressive disorder, current episode severe without psychotic symptoms | 530 |
| Eu333 | [X]Recurrent depressive disorder, current episode severe with psychotic symptoms | 355 |
| Eu334 | [X]Recurrent depressive disorder, currently in remission | 94 |
| Eu33y | [X]Other recurrent depressive disorders | 35 |
| Eu33z | [X]Recurrent depressive disorder, unspecified | 153 |
| Eu341 | [X]Dysthymia | 2032 |
| Eu412 | [X]Mixed anxiety and depressive disorder | 4285 |
| Eu530 | [X]Mild mental and behavioural disorders associated with the puerperium, not elsewhere classified | 56 |
| Eu920 | [X]Depressive conduct disorder | <5 |

### ICD 9 and 10 codes:

| code | desc | total\_n |
| --- | --- | --- |
| 2962 | Major depressive disorder single episode | 0 |
| 2963 | Major depressive disorder recurrent episode | 0 |
| 2969 | Unspecified | <5 |
| F32 | Depressive episode | 58 |
| F32. | NA | 43 |
| F320 | Mild depressive episode | 1512 |
| F321 | Moderate depressive episode | 2335 |
| F322 | Severe depressive episode without psychotic symptoms | 2075 |
| F323 | Severe depressive episode with psychotic symptoms | 2147 |
| F325 | NA | <5 |
| F328 | Other depressive episodes | 289 |
| F329 | Depressive episode unspecified | 65766 |
| F32X | NA | 129 |
| F33 | Recurrent depressive disorder | 35 |
| F33. | NA | 32 |
| F330 | Recurrent depressive disorder current episode mild | 636 |
| F331 | Recurrent depressive disorder current episode moderate | 1136 |
| F332 | Recurrent depressive disorder current episode severe without psychotic symptoms | 816 |
| F333 | Recurrent depressive disorder current episode severe with psychotic symptoms | 747 |
| F334 | Recurrent depressive disorder currently in remission | 120 |
| F338 | Other recurrent depressive disorders | 65 |
| F339 | Recurrent depressive disorder unspecified | 2986 |
| F33X | NA | 57 |
| F38 | Other mood [affective] disorders | <5 |
| F380 | Other single mood [affective] disorders | 23 |
| F381 | Other recurrent mood [affective] disorders | 21 |
| F388 | Other specified mood [affective] disorders | 44 |
| F39 | Unspecified mood [affective] disorder | 9 |
| F396 | NA | <5 |
| F39X | NA | 269 |

## Descriptives

272411 people had at least one diagnostic code in at least one of the datasets. 71216 people had a code in hospital admissions data, 895 in mortality data and 246345 in primary care data. The following figure shows the year of the first code that was found for any person classified positive using (a) all codes combined, (b) only codes from hospital admissions data, (c) only codes from the mortality data and (d) only codes from primary care data.
