# Supplementary material for: The Secure Anonymised Information Linkage databank Dementia e-cohort (SAIL-DeC)
Source: Int J Popul Data Sci. 2020 Feb 25;5(1):1121. doi: 10.23889/ijpds.v5i1.1121 (PMC7473277; doi:10.23889/ijpds.v5i1.1121)
Supplement: Supplementary Material [file ijpds-05-01-1121-s001.zip › Supplementary Appendix 15.html]

Event tables


# Event tables

### *Diabetes*

#### *Christian*

#### *January 2019*

## Code selection

We have selected codes based on Eastwood et al., 2016, Algorithms for the capture and adjudication of prevalent and incident diabetes in UK Biobank. PLOS One, 11(9) in conjunction with the WHO ICD 10 browser (apps.who.int/classifications/icd10/browse/2010/en) and the NHS Read Code Browser (https://isd.digital.nhs.uk/trud3/user/guest/group/0/home). In contrast to Eastwood et al, we have included codes for malnutrition-related diabetes. We have deliberately included codes with obvious `misspelling’ (for example having a dot where none should be) or ICD 10 codes ending with ‘X’.

We have not further subtyped diabetes mellitus into Type 1 and Type 2 DM, because the optimal code selection depends on the ultimate goal of the research. Please see Eastwood et al., 2016 for a suggestion of subclassification.

All codes that were selected for classification and the total number of people with at least one of the codes are displayed in the following tables. Please be aware that frequency counts of Read V2 codes in the table do not reflect the hierarchical nature of Read V2 coding (for example, counts of E01.. do not include E011.).

### Read V2 codes:

| code | desc | total\_n |
| --- | --- | --- |
| C10.. | Diabetes mellitus | 89844 |
| C100. | Diabetes mellitus with no mention of complication | 1964 |
| C1000 | Diabetes mellitus, juvenile type, with no mention of complication | 3112 |
| C1001 | Diabetes mellitus, adult onset, with no mention of complication | 16721 |
| C100z | Diabetes mellitus NOS with no mention of complication | 52 |
| C101. | Diabetes mellitus with ketoacidosis | 833 |
| C1010 | Diabetes mellitus, juvenile type, with ketoacidosis | <5 |
| C1011 | Diabetes mellitus, adult onset, with ketoacidosis | 27 |
| C101y | Other specified diabetes mellitus with ketoacidosis | 6 |
| C101z | Diabetes mellitus NOS with ketoacidosis | 30 |
| C102. | Diabetes mellitus with hyperosmolar coma | 46 |
| C1020 | Diabetes mellitus, juvenile type, with hyperosmolar coma | <5 |
| C1021 | Diabetes mellitus, adult onset, with hyperosmolar coma | 7 |
| C102z | Diabetes mellitus NOS with hyperosmolar coma | 6 |
| C103. | Diabetes mellitus with ketoacidotic coma | 48 |
| C1030 | Diabetes mellitus, juvenile type, with ketoacidotic coma | 0 |
| C1031 | Diabetes mellitus, adult onset, with ketoacidotic coma | <5 |
| C103y | Other specified diabetes mellitus with coma | <5 |
| C103z | Diabetes mellitus NOS with ketoacidotic coma | <5 |
| C104. | Diabetes mellitus with renal manifestation | 2572 |
| C1040 | Diabetes mellitus, juvenile type, with renal manifestation | <5 |
| C1041 | Diabetes mellitus, adult onset, with renal manifestation | 30 |
| C104y | Other specified diabetes mellitus with renal complications | 8 |
| C104z | Diabetes mellitus with nephropathy NOS | 124 |
| C105. | Diabetes mellitus with ophthalmic manifestation | 753 |
| C1050 | Diabetes mellitus, juvenile type, with ophthalmic manifestation | 5 |
| C1051 | Diabetes mellitus, adult onset, with ophthalmic manifestation | 36 |
| C105y | Other specified diabetes mellitus with ophthalmic complications | 0 |
| C105z | Diabetes mellitus NOS with ophthalmic manifestation | <5 |
| C106. | Diabetes mellitus with neurological manifestation | 3400 |
| C1060 | Diabetes mellitus, juvenile type, with neurological manifestation | <5 |
| C1061 | Diabetes mellitus, adult onset, with neurological manifestation | 72 |
| C106y | Other specified diabetes mellitus with neurological complications | 39 |
| C106z | Diabetes mellitus NOS with neurological manifestation | 81 |
| C107. | Diabetes mellitus with peripheral circulatory disorder | 448 |
| C1070 | Diabetes mellitus, juvenile type, with peripheral circulatory disorder | <5 |
| C1071 | Diabetes mellitus, adult onset, with peripheral circulatory disorder | 24 |
| C1072 | Diabetes mellitus, adult with gangrene | 30 |
| C1073 | IDDM with peripheral circulatory disorder | 17 |
| C1074 | NIDDM with peripheral circulatory disorder | 52 |
| C107y | Other specified diabetes mellitus with peripheral circulatory complications | 0 |
| C107z | Diabetes mellitus NOS with peripheral circulatory disorder | 26 |
| C108. | Insulin dependent diabetes mellitus | 5062 |
| C1080 | Insulin-dependent diabetes mellitus with renal complications | 10 |
| C1081 | Insulin-dependent diabetes mellitus with ophthalmic complications | 10 |
| C1082 | Insulin-dependent diabetes mellitus with neurological complications | 11 |
| C1083 | Insulin dependent diabetes mellitus with multiple complications | 6 |
| C1084 | Unstable insulin dependent diabetes mellitus | 9 |
| C1085 | Insulin dependent diabetes mellitus with ulcer | 25 |
| C1086 | Insulin dependent diabetes mellitus with gangrene | <5 |
| C1087 | Insulin dependent diabetes mellitus with retinopathy | 60 |
| C1088 | Insulin dependent diabetes mellitus - poor control | 104 |
| C1089 | Insulin dependent diabetes maturity onset | 81 |
| C108A | Insulin-dependent diabetes without complication | 6 |
| C108B | Insulin dependent diabetes mellitus with mononeuropathy | 5 |
| C108C | Insulin dependent diabetes mellitus with polyneuropathy | <5 |
| C108D | Insulin dependent diabetes mellitus with nephropathy | 17 |
| C108E | Insulin dependent diabetes mellitus with hypoglycaemic coma | 10 |
| C108F | Insulin dependent diabetes mellitus with diabetic cataract | 7 |
| C108G | Insulin dependent diabetes mellitus with peripheral angiopathy | <5 |
| C108H | Insulin dependent diabetes mellitus with arthropathy | <5 |
| C108J | Insulin dependent diabetes mellitus with neuropathic arthropathy | <5 |
| C108y | Other specified diabetes mellitus with multiple complications | <5 |
| C108z | Unspecified diabetes mellitus with multiple complications | 0 |
| C109. | Non-insulin dependent diabetes mellitus | 40774 |
| C1090 | Non-insulin-dependent diabetes mellitus with renal complications | 40 |
| C1091 | Non-insulin-dependent diabetes mellitus with ophthalmic complications | 17 |
| C1092 | Non-insulin-dependent diabetes mellitus with neurological complications | 30 |
| C1093 | Non-insulin-dependent diabetes mellitus with multiple complications | 6 |
| C1094 | Non-insulin dependent diabetes mellitus with ulcer | 55 |
| C1095 | Non-insulin dependent diabetes mellitus with gangrene | 18 |
| C1096 | Non-insulin-dependent diabetes mellitus with retinopathy | 78 |
| C1097 | Non-insulin dependent diabetes mellitus - poor control | 739 |
| C1098 | Reaven’s syndrome | 0 |
| C1099 | Non-insulin-dependent diabetes mellitus without complication | 203 |
| C109A | Non-insulin dependent diabetes mellitus with mononeuropathy | <5 |
| C109B | Non-insulin dependent diabetes mellitus with polyneuropathy | 17 |
| C109C | Non-insulin dependent diabetes mellitus with nephropathy | 81 |
| C109D | Non-insulin dependent diabetes mellitus with hypoglycaemic coma | 15 |
| C109E | Non-insulin dependent diabetes mellitus with diabetic cataract | 80 |
| C109F | Non-insulin-dependent diabetes mellitus with peripheral angiopathy | 5 |
| C109G | Non-insulin dependent diabetes mellitus with arthropathy | <5 |
| C109H | Non-insulin dependent diabetes mellitus with neuropathic arthropathy | 7 |
| C109J | Insulin treated Type 2 diabetes mellitus | 2475 |
| C109K | Hyperosmolar non-ketotic state in type 2 diabetes mellitus | 17 |
| C10A. | Malnutrition-related diabetes mellitus | 22 |
| C10A0 | Malnutrition-related diabetes mellitus with coma | 0 |
| C10A1 | Malnutrition-related diabetes mellitus with ketoacidosis | 8 |
| C10A2 | Malnutrition-related diabetes mellitus with renal complications | 0 |
| C10A3 | Malnutrition-related diabetes mellitus with ophthalmic complications | 0 |
| C10A4 | Malnutrition-related diabetes mellitus with neurological complications | <5 |
| C10A5 | Malnutrition-related diabetes mellitus with peripheral circulatory complications | 0 |
| C10A6 | Malnutrition-related diabetes mellitus with multiple complications | 0 |
| C10A7 | Malnutrition-related diabetes mellitus without complications | 0 |
| C10AW | Malnutrition-related diabetes mellitus with unspecified complications | <5 |
| C10AX | Malnutrition-related diabetes mellitus with other specified complications | 0 |
| C10B. | Diabetes mellitus induced by steroids | 224 |
| C10B0 | Steroid induced diabetes mellitus without complication | 11 |
| C10C. | Diabetes mellitus autosomal dominant | 9 |
| C10D. | Diabetes mellitus autosomal dominant type 2 | 107 |
| C10E. | Type 1 diabetes mellitus | 6575 |
| C10E0 | Type 1 diabetes mellitus with renal complications | 7 |
| C10E1 | Type 1 diabetes mellitus with ophthalmic complications | 6 |
| C10E2 | Type 1 diabetes mellitus with neurological complications | 6 |
| C10E3 | Type 1 diabetes mellitus with multiple complications | 8 |
| C10E4 | Unstable type 1 diabetes mellitus | 11 |
| C10E5 | Type 1 diabetes mellitus with ulcer | 8 |
| C10E6 | Type 1 diabetes mellitus with gangrene | <5 |
| C10E7 | Type 1 diabetes mellitus with retinopathy | 51 |
| C10E8 | Type 1 diabetes mellitus - poor control | 27 |
| C10E9 | Type 1 diabetes mellitus maturity onset | 65 |
| C10EA | Type 1 diabetes mellitus without complication | <5 |
| C10EB | Type 1 diabetes mellitus with mononeuropathy | 0 |
| C10EC | Type 1 diabetes mellitus with polyneuropathy | 7 |
| C10ED | Type 1 diabetes mellitus with nephropathy | 42 |
| C10EE | Type 1 diabetes mellitus with hypoglycaemic coma | 18 |
| C10EF | Type 1 diabetes mellitus with diabetic cataract | 11 |
| C10EG | Type 1 diabetes mellitus with peripheral angiopathy | <5 |
| C10EH | Type 1 diabetes mellitus with arthropathy | <5 |
| C10EJ | Type 1 diabetes mellitus with neuropathic arthropathy | 16 |
| C10EK | Type 1 diabetes mellitus with persistent proteinuria | 64 |
| C10EL | Type 1 diabetes mellitus with persistent microalbuminuria | 88 |
| C10EM | Type 1 diabetes mellitus with ketoacidosis | 148 |
| C10EN | Type 1 diabetes mellitus with ketoacidotic coma | 15 |
| C10EP | Type 1 diabetes mellitus with exudative maculopathy | 21 |
| C10EQ | Type 1 diabetes mellitus with gastroparesis | 19 |
| C10ER | Latent autoimmune diabetes mellitus in adult | 45 |
| C10F. | Type 2 diabetes mellitus | 166450 |
| C10F0 | Type 2 diabetes mellitus with renal complications | 59 |
| C10F1 | Type 2 diabetes mellitus with ophthalmic complications | 16 |
| C10F2 | Type 2 diabetes mellitus with neurological complications | 49 |
| C10F3 | Type 2 diabetes mellitus with multiple complications | 12 |
| C10F4 | Type 2 diabetes mellitus with ulcer | 50 |
| C10F5 | Type 2 diabetes mellitus with gangrene | 17 |
| C10F6 | Type 2 diabetes mellitus with retinopathy | 724 |
| C10F7 | Type 2 diabetes mellitus - poor control | 485 |
| C10F8 | Reaven’s syndrome | 62 |
| C10F9 | Type 2 diabetes mellitus without complication | 146 |
| C10FA | Type 2 diabetes mellitus with mononeuropathy | 18 |
| C10FB | Type 2 diabetes mellitus with polyneuropathy | 34 |
| C10FC | Type 2 diabetes mellitus with nephropathy | 450 |
| C10FD | Type 2 diabetes mellitus with hypoglycaemic coma | 35 |
| C10FE | Type 2 diabetes mellitus with diabetic cataract | 66 |
| C10FF | Type 2 diabetes mellitus with peripheral angiopathy | 8 |
| C10FG | Type 2 diabetes mellitus with arthropathy | 15 |
| C10FH | Type 2 diabetes mellitus with neuropathic arthropathy | 56 |
| C10FJ | Insulin treated Type 2 diabetes mellitus | 8190 |
| C10FK | Hyperosmolar non-ketotic state in type 2 diabetes mellitus | 94 |
| C10FL | Type 2 diabetes mellitus with persistent proteinuria | 1190 |
| C10FM | Type 2 diabetes mellitus with persistent microalbuminuria | 3068 |
| C10FN | Type 2 diabetes mellitus with ketoacidosis | 134 |
| C10FP | Type 2 diabetes mellitus with ketoacidotic coma | 5 |
| C10FQ | Type 2 diabetes mellitus with exudative maculopathy | 106 |
| C10FR | Type 2 diabetes mellitus with gastroparesis | 44 |
| C10FS | Maternally inherited diabetes mellitus | <5 |
| C10G. | Secondary pancreatic diabetes mellitus | 38 |
| C10G0 | Secondary pancreatic diabetes mellitus without complication | <5 |
| C10H. | Diabetes mellitus induced by non-steroid drugs | <5 |
| C10H0 | Diabetes mellitus induced by non-steroid drugs without complication | 0 |
| C10J. | Insulin autoimmune syndrome | <5 |
| C10J0 | Insulin autoimmune syndrome without complication | <5 |
| C10K. | Type A insulin resistance | <5 |
| C10K0 | Type A insulin resistance without complication | <5 |
| C10L. | Fibrocalculous pancreatopathy | 0 |
| C10L0 | Fibrocalculous pancreatopathy without complication | 0 |
| C10M. | Lipoatrophic diabetes mellitus | <5 |
| C10M0 | Lipoatrophic diabetes mellitus without complication | 0 |
| C10N. | Secondary diabetes mellitus | 24 |
| C10N0 | Secondary diabetes mellitus without complication | 0 |
| C10N1 | Cystic fibrosis related diabetes mellitus | 0 |
| C10P. | Diabetes mellitus in remission | 246 |
| C10P0 | Type I diabetes mellitus in remission | <5 |
| C10P1 | Type II diabetes mellitus in remission | 206 |
| C10Q. | Maturity onset diabetes of the young type 5 | 0 |
| C10y. | Diabetes mellitus with other specified manifestation | 43 |
| C10y0 | Diabetes mellitus, juvenile type, with other specified manifestation | <5 |
| C10y1 | Diabetes mellitus, adult onset, with other specified manifestation | 8 |
| C10yy | Other specified diabetes mellitus with other specified complications | <5 |
| C10yz | Diabetes mellitus NOS with other specified manifestation | <5 |
| C10z. | Diabetes mellitus with unspecified complication | 40 |
| C10z0 | Diabetes mellitus, juvenile type, with unspecified complication | <5 |
| C10z1 | Diabetes mellitus, adult onset, with unspecified complication | 11 |
| C10zy | Other specified diabetes mellitus with unspecified complications | <5 |
| C10zz | Diabetes mellitus NOS with unspecified complication | <5 |

### ICD 9 and 10 codes:

The following fourth-character subdivisions are for use with categories E10-E14: 0 = with coma; 1 = with ketoacidosis; 2 = with renal complications; 3 = with ophtalmic complications; 4 = with neurological complications; 5 = with peripheral circulatory complications; 6 = with other specified complications; 7 = with multiple complications; 8 = with unspecified complications; 9 = without complications.

Please consult ICD 10 documentation for futher information.

| code | desc | total\_n |
| --- | --- | --- |
| 250 | Diabetes mellitus | 0 |
| 2500 | Diabetes mellitus without mention of complication | 3220 |
| 2501 | Diabetes with ketoacidosis | 37 |
| 2502 | Diabetes with coma | 23 |
| 2503 | Diabetes with renal manifestations | 49 |
| 2504 | Diabetes with ophthalmic manifestations | 12 |
| 2505 | Diabetes with neurological manifestations | 7 |
| 2506 | Diabetes with peripheral circulatory disorders | 103 |
| 2507 | Diabetes with other specified manifestations | 35 |
| 2508 | NA | <5 |
| 2509 | Diabetes with unspecified complications | 22 |
| 3572 | NA | <5 |
| 3572A | Polyneuropathy in diabetes | 0 |
| 3620 | NA | <5 |
| 3620A | Diabetic retinopathy | 0 |
| E10 | Type 1 diabetes mellitus | 24 |
| E10- | NA | <5 |
| E10. | NA | <5 |
| E100 | Type 1 diabetes mellitus | 538 |
| E101 | Type 1 diabetes mellitus | 1486 |
| E102 | Type 1 diabetes mellitus | 1217 |
| E103 | Type 1 diabetes mellitus | 4942 |
| E104 | Type 1 diabetes mellitus | 1563 |
| E105 | Type 1 diabetes mellitus | 2079 |
| E106 | Type 1 diabetes mellitus | 586 |
| E107 | Type 1 diabetes mellitus | 152 |
| E108 | Type 1 diabetes mellitus | 1381 |
| E109 | Type 1 diabetes mellitus | 20932 |
| E10D | NA | 25 |
| E10X | NA | 46 |
| E11 | Type 2 diabetes mellitus | 64 |
| E11- | NA | <5 |
| E11. | NA | 14 |
| E110 | Type 2 diabetes mellitus | 1486 |
| E111 | Type 2 diabetes mellitus | 2006 |
| E112 | Type 2 diabetes mellitus | 5131 |
| E113 | Type 2 diabetes mellitus | 14922 |
| E114 | Type 2 diabetes mellitus | 6739 |
| E115 | Type 2 diabetes mellitus | 7947 |
| E116 | Type 2 diabetes mellitus | 1106 |
| E117 | Type 2 diabetes mellitus | 224 |
| E118 | Type 2 diabetes mellitus | 2150 |
| E119 | Type 2 diabetes mellitus | 170873 |
| E11D | NA | 51 |
| E11X | NA | 38 |
| E12 | Malnutrition-related diabetes mellitus | 0 |
| E120 | Malnutrition-related diabetes mellitus | 6 |
| E121 | Malnutrition-related diabetes mellitus | <5 |
| E122 | Malnutrition-related diabetes mellitus | <5 |
| E123 | Malnutrition-related diabetes mellitus | <5 |
| E124 | Malnutrition-related diabetes mellitus | <5 |
| E125 | Malnutrition-related diabetes mellitus | 0 |
| E126 | Malnutrition-related diabetes mellitus | 0 |
| E127 | Malnutrition-related diabetes mellitus | 0 |
| E128 | Malnutrition-related diabetes mellitus | 8 |
| E129 | Malnutrition-related diabetes mellitus | 33 |
| E13 | Other specified diabetes mellitus | 0 |
| E130 | Other specified diabetes mellitus | 11 |
| E131 | Other specified diabetes mellitus | 15 |
| E132 | Other specified diabetes mellitus | 12 |
| E133 | Other specified diabetes mellitus | 51 |
| E134 | Other specified diabetes mellitus | 11 |
| E135 | Other specified diabetes mellitus | 9 |
| E136 | Other specified diabetes mellitus | 11 |
| E137 | Other specified diabetes mellitus | <5 |
| E138 | Other specified diabetes mellitus | 26 |
| E139 | Other specified diabetes mellitus | 1101 |
| E13X | NA | <5 |
| E14 | Unspecified diabetes mellitus | 31 |
| E14. | NA | <5 |
| E140 | Unspecified diabetes mellitus | 247 |
| E141 | Unspecified diabetes mellitus | 275 |
| E142 | Unspecified diabetes mellitus | 665 |
| E143 | Unspecified diabetes mellitus | 3065 |
| E144 | Unspecified diabetes mellitus | 208 |
| E145 | Unspecified diabetes mellitus | 796 |
| E146 | Unspecified diabetes mellitus | 91 |
| E147 | Unspecified diabetes mellitus | 111 |
| E148 | Unspecified diabetes mellitus | 215 |
| E149 | Unspecified diabetes mellitus | 22863 |
| E14X | NA | 20 |

## Descriptives

237745 people had at least one diagnostic code in at least one of the datasets. 185062 people had a code in hospital admissions data, 29353 in mortality data and 200132 in primary care data. The following figure shows the year of the first code that was found for any person classified positive using (a) all codes combined, (b) only codes from hospital admissions data, (c) only codes from the mortality data and (d) only codes from primary care data.
