# Supplementary material for: The Secure Anonymised Information Linkage databank Dementia e-cohort (SAIL-DeC)
Source: Int J Popul Data Sci. 2020 Feb 25;5(1):1121. doi: 10.23889/ijpds.v5i1.1121 (PMC7473277; doi:10.23889/ijpds.v5i1.1121)
Supplement: Supplementary Material [file ijpds-05-01-1121-s001.zip › Supplementary Appendix 16.html]

Event tables


# Event tables

### *Epilepsy*

#### *Christian*

#### *January 2019*

## Code selection

We have selected codes based on QOF Business rules v24 https://www.pcc-cic.org.uk/article/qof-business-rules-v240 in conjunction with the WHO ICD 10 browser (apps.who.int/classifications/icd10/browse/2010/en) and the NHS Read Code Browser (https://isd.digital.nhs.uk/trud3/user/guest/group/0/home). We have deliberately included codes with obvious `misspelling’ (for example having a dot where none should be) or ICD 10 codes ending with ‘X’.

Note: This code list also contains codes for childhood epilepsies and seizures (some of which may be provoked or isolated seizures and do not therefore constitute epilepsy) - researchers may want to exclude these depending on the specific research question.

All codes that were selected for classification and the total number of people with at least one of the codes are displayed in the following tables. Please be aware that frequency counts of Read V2 codes in the table do not reflect the hierarchical nature of Read V2 coding (for example, counts of E01.. do not include E011.).

### Read V2 codes:

| code | desc | total\_n |
| --- | --- | --- |
| F1321 | Progressive myoclonic epilepsy | 9 |
| F25.. | Epilepsy | 20724 |
| F250. | Generalised nonconvulsive epilepsy | 44 |
| F2500 | Petit mal (minor) epilepsy | 1048 |
| F2501 | Pykno-epilepsy | 0 |
| F2502 | Epileptic seizures - atonic | 48 |
| F2503 | Epileptic seizures - akinetic | 25 |
| F2504 | Juvenile absence epilepsy | 6 |
| F2505 | Lennox-Gastaut syndrome | 0 |
| F250y | Other specified generalised nonconvulsive epilepsy | 5 |
| F250z | Generalised nonconvulsive epilepsy NOS | 11 |
| F251. | Generalised convulsive epilepsy | 262 |
| F2510 | Grand mal (major) epilepsy | 2465 |
| F2511 | Neonatal myoclonic epilepsy | <5 |
| F2512 | Epileptic seizures - clonic | 322 |
| F2513 | Epileptic seizures - myoclonic | 101 |
| F2514 | Epileptic seizures - tonic | 420 |
| F2515 | Tonic-clonic epilepsy | 315 |
| F2516 | Grand mal seizure | 1881 |
| F251y | Other specified generalised convulsive epilepsy | 19 |
| F251z | Generalised convulsive epilepsy NOS | 57 |
| F252. | Petit mal status | 133 |
| F253. | Grand mal status | 542 |
| F254. | Partial epilepsy with impairment of consciousness | 147 |
| F2540 | Temporal lobe epilepsy | 1467 |
| F2541 | Psychomotor epilepsy | 16 |
| F2542 | Psychosensory epilepsy | <5 |
| F2543 | Limbic system epilepsy | <5 |
| F2544 | Epileptic automatism | 8 |
| F2545 | Complex partial epileptic seizure | 657 |
| F254z | Partial epilepsy with impairment of consciousness NOS | 21 |
| F255. | Partial epilepsy without mention of impairment of consciousness | 119 |
| F2550 | Jacksonian, focal or motor epilepsy | 605 |
| F2551 | Sensory induced epilepsy | 6 |
| F2552 | Somatosensory epilepsy | <5 |
| F2553 | Visceral reflex epilepsy | 0 |
| F2554 | Visual reflex epilepsy | <5 |
| F2555 | Unilateral epilepsy | <5 |
| F2556 | Simple partial epileptic seizure | 130 |
| F255y | Other specified partial epilepsy without mention of impairment of consciousness | 7 |
| F255z | Partial epilepsy without mention of impairment of consciousness NOS | 15 |
| F256. | Infantile spasms | 13 |
| F2560 | Hypsarrhythmia | <5 |
| F2561 | Salaam attacks | <5 |
| F256z | Infantile spasms NOS | 0 |
| F257. | Kojevnikov’s epilepsy | 0 |
| F258. | Post-ictal state | 133 |
| F259. | Early infantile epileptic encephalopathy with suppression bursts | <5 |
| F25A. | Juvenile myoclonic epilepsy | 13 |
| F25B. | Alcohol-induced epilepsy | 48 |
| F25C. | Drug-induced epilepsy | <5 |
| F25D. | Menstrual epilepsy | <5 |
| F25E. | Stress-induced epilepsy | 11 |
| F25F. | Photosensitive epilepsy | 15 |
| F25G. | Severe myoclonic epilepsy in infancy | 0 |
| F25H. | Generalised seizure | 160 |
| F25X. | Status epilepticus, unspecified | 183 |
| F25y. | Other forms of epilepsy | 112 |
| F25y0 | Cursive (running) epilepsy | 0 |
| F25y1 | Gelastic epilepsy | <5 |
| F25y2 | Localization-related(focal)(partial)idiopathic epilepsy and epileptic syndromes with seizures of localised onset | 40 |
| F25y3 | Complex partial status epilepticus | 77 |
| F25y4 | Benign Rolandic epilepsy | 0 |
| F25y5 | Panayiotopoulos syndrome | 0 |
| F25yz | Other forms of epilepsy NOS | 77 |
| F25z. | Epilepsy NOS | 2447 |
| SC200 | Traumatic epilepsy | 223 |

### ICD 9 and 10 codes:

| code | desc | total\_n |
| --- | --- | --- |
| 345 | Epilepsy | 0 |
| 3450 | Generalized nonconvulsive epilepsy | <5 |
| 3451 | Generalized convulsive epilepsy | 14 |
| 3452 | Petit mal status | 0 |
| 3453 | Grand mal status | 11 |
| 3454 | Partial epilepsy with impairment of consciousness | <5 |
| 3455 | Partial epilepsy without mention of impairment of | 0 |
| 3456 | Infantile spasms | 0 |
| 3457 | Epilepsia partialis continua | 0 |
| 3458 | Other | <5 |
| 3459 | Unspecified | 218 |
| G40 | Epilepsy | 10 |
| G40. | NA | <5 |
| G400 | Localization-related (focal)(partial) idiopathic epilepsy and epileptic syndromes with seizures of localized onset | 215 |
| G401 | Localization-related (focal)(partial) symptomatic epilepsy and epileptic syndromes with simple partial seizures | 795 |
| G402 | Localization-related (focal)(partial) symptomatic epilepsy and epileptic syndromes with complex partial seizures | 1127 |
| G403 | Generalized idiopathic epilepsy and epileptic syndromes | 3437 |
| G404 | Other generalized epilepsy and epileptic syndromes | 142 |
| G405 | Special epileptic syndromes | 750 |
| G406 | Grand mal seizures unspecified (with or without petit mal) | 2345 |
| G407 | Petit mal unspecified without grand mal seizures | 359 |
| G408 | Other epilepsy | 990 |
| G409 | Epilepsy unspecified | 26092 |
| G40X | NA | 12 |
| G41 | Status epilepticus | 0 |
| G410 | Grand mal status epilepticus | 246 |
| G411 | Petit mal status epilepticus | 20 |
| G412 | Complex partial status epilepticus | 36 |
| G418 | Other status epilepticus | 59 |
| G419 | Status epilepticus unspecified | 860 |

## Descriptives

40270 people had at least one diagnostic code in at least one of the datasets. 30240 people had a code in hospital admissions data, 1649 in mortality data and 25401 in primary care data. The following figure shows the year of the first code that was found for any person classified positive using (a) all codes combined, (b) only codes from hospital admissions data, (c) only codes from the mortality data and (d) only codes from primary care data.
