# Supplementary material for: The Secure Anonymised Information Linkage databank Dementia e-cohort (SAIL-DeC)
Source: Int J Popul Data Sci. 2020 Feb 25;5(1):1121. doi: 10.23889/ijpds.v5i1.1121 (PMC7473277; doi:10.23889/ijpds.v5i1.1121)
Supplement: Supplementary Material [file ijpds-05-01-1121-s001.zip › Supplementary Appendix 17.html]

Event tables


# Event tables

### *Heart failure*

#### *Christian*

#### *January 2019*

## Code selection

We have selected codes based on QOF Business rules v24 https://www.pcc-cic.org.uk/article/qof-business-rules-v240 in conjunction with the WHO ICD 10 browser (apps.who.int/classifications/icd10/browse/2010/en) and the NHS Read Code Browser (https://isd.digital.nhs.uk/trud3/user/guest/group/0/home). We have deliberately included codes with obvious `misspelling’ (for example having a dot where none should be) or ICD 10 codes ending with ‘X’.

All codes that were selected for classification and the total number of people with at least one of the codes are displayed in the following tables. Please be aware that frequency counts of Read V2 codes in the table do not reflect the hierarchical nature of Read V2 coding (for example, counts of E01.. do not include E011.).

### Read V2 codes:

| code | desc | total\_n |
| --- | --- | --- |
| 662f. | New York Heart Association classification - class I | 1324 |
| 662g. | New York Heart Association classification - class II | 3486 |
| 662h. | New York Heart Association classification - class III | 2409 |
| 662i. | New York Heart Association classification - class IV | 317 |
| G1yz1 | Rheumatic left ventricular failure | 40 |
| G58.. | Heart failure | 29073 |
| G580. | Congestive heart failure | 49433 |
| G5800 | Acute congestive heart failure | 373 |
| G5801 | Chronic congestive heart failure | 987 |
| G5802 | Decompensated cardiac failure | 595 |
| G5803 | Compensated cardiac failure | 85 |
| G5804 | Congestive heart failure due to valvular disease | 34 |
| G581. | Left ventricular failure | 38217 |
| G5810 | Acute left ventricular failure | 967 |
| G582. | Acute heart failure | 175 |
| G583. | Heart failure with normal ejection fraction | 553 |
| G584. | Right ventricular failure | 130 |
| G58z. | Heart failure NOS | 1646 |

### ICD 9 and 10 codes:

| code | desc | total\_n |
| --- | --- | --- |
| 428 | Heart failure | 0 |
| 4280 | Congestive heart failure | 4758 |
| 4281 | Left heart failure | 1927 |
| 4284 | NA | <5 |
| 4289 | Unspecified | 1714 |
| I110 | Hypertensive heart disease with (congestive) heart failure | 3283 |
| I130 | Hypertensive heart and renal disease with (congestive) heart failure | 164 |
| I132 | Hypertensive heart and renal disease with both (congestive) heart failure and renal failure | 577 |
| I500 | Congestive heart failure | 101744 |
| I501 | Left ventricular failure | 73529 |
| I509 | Heart failure unspecified | 35778 |

## Descriptives

195806 people had at least one diagnostic code in at least one of the datasets. 151053 people had a code in hospital admissions data, 48807 in mortality data and 98908 in primary care data. The following figure shows the year of the first code that was found for any person classified positive using (a) all codes combined, (b) only codes from hospital admissions data, (c) only codes from the mortality data and (d) only codes from primary care data.
