# Supplementary material for: The Secure Anonymised Information Linkage databank Dementia e-cohort (SAIL-DeC)
Source: Int J Popul Data Sci. 2020 Feb 25;5(1):1121. doi: 10.23889/ijpds.v5i1.1121 (PMC7473277; doi:10.23889/ijpds.v5i1.1121)
Supplement: Supplementary Material [file ijpds-05-01-1121-s001.zip › Supplementary Appendix 18.html]

Event tables


# Event tables

### *Hypertension*

#### *Christian*

#### *January 2019*

## Code selection

We have selected codes based on QOF Business rules v24 https://www.pcc-cic.org.uk/article/qof-business-rules-v240 in conjunction with the WHO ICD 10 browser (apps.who.int/classifications/icd10/browse/2010/en) and the NHS Read Code Browser (https://isd.digital.nhs.uk/trud3/user/guest/group/0/home). However, the following codes were excluded as we considered them likely to produce false positive cases: 662G. (Hypertensive treatm.changed) and 662H. (Hypertensive treatm.stopped). We have deliberately included codes with obvious `misspelling’ (for example having a dot where none should be) or ICD 10 codes ending with ‘X’.

All codes that were selected for classification and the total number of people with at least one of the codes are displayed in the following tables. Please be aware that frequency counts of Read V2 codes in the table do not reflect the hierarchical nature of Read V2 coding (for example, counts of E01.. do not include E011.).

### Read V2 codes:

| code | desc | total\_n |
| --- | --- | --- |
| G2… | Hypertensive disease | 262461 |
| G20.. | Essential hypertension | 379262 |
| G200. | Malignant essential hypertension | 594 |
| G201. | Benign essential hypertension | 10910 |
| G202. | Systolic hypertension | 3461 |
| G203. | Diastolic hypertension | 67 |
| G20z. | Essential hypertension NOS | 25468 |
| G21.. | Hypertensive heart disease | 1168 |
| G210. | Malignant hypertensive heart disease | 12 |
| G2100 | Malignant hypertensive heart disease without congestive cardiac failure | <5 |
| G2101 | Malignant hypertensive heart disease with congestive cardiac failure | <5 |
| G210z | Malignant hypertensive heart disease NOS | 0 |
| G211. | Benign hypertensive heart disease | 15 |
| G2110 | Benign hypertensive heart disease without congestive cardiac failure | <5 |
| G2111 | Benign hypertensive heart disease with congestive cardiac failure | 48 |
| G211z | Benign hypertensive heart disease NOS | <5 |
| G21z. | Hypertensive heart disease NOS | 35 |
| G21z0 | Hypertensive heart disease NOS without congestive cardic failure | 398 |
| G21z1 | Hypertensive heart disease NOS with congestive cardiac failure | 125 |
| G21zz | Hypertensive heart disease NOS | 178 |
| G22.. | Hypertensive renal disease | 809 |
| G220. | Malignant hypertensive renal disease | 20 |
| G221. | Benign hypertensive renal disease | 15 |
| G222. | Hypertensive renal disease with renal failure | 17 |
| G22z. | Hypertensive renal disease NOS | 111 |
| G23.. | Hypertensive heart and renal disease | 41 |
| G230. | Malignant hypertensive heart and renal disease | <5 |
| G231. | Benign hypertensive heart and renal disease | 0 |
| G232. | Hypertensive heart and renal disease with (congestive) heart failure | 5 |
| G233. | Hypertensive heart and renal disease with renal failure | 11 |
| G234. | Hypertensive heart and renal disease with both (congestive) heart failure and renal failure | <5 |
| G23z. | Hypertensive heart and renal disease NOS | <5 |
| G24.. | Secondary hypertension | 317 |
| G240. | Secondary malignant hypertension | 10 |
| G2400 | Secondary malignant renovascular hypertension | 5 |
| G240z | Secondary malignant hypertension NOS | 7 |
| G241. | Secondary benign hypertension | 13 |
| G2410 | Secondary benign renovascular hypertension | 48 |
| G241z | Secondary benign hypertension NOS | 10 |
| G244. | Hypertension secondary to endocrine disorders | 6 |
| G24z. | Secondary hypertension NOS | 17 |
| G24z0 | Secondary renovascular hypertension NOS | 36 |
| G24z1 | Hypertension secondary to drug | 15 |
| G24zz | Secondary hypertension NOS | 18 |
| G25.. | Stage 1 hypertension (NICE - National Institute for Health and Clinical Excellence 2011) | 420 |
| G250. | Stage 1 hypertension (NICE 2011) without evidence of end organ damage | 71 |
| G251. | Stage 1 hypertension (NICE 2011) with evidence of end organ damage | 7 |
| G26.. | Severe hypertension (NICE - National Institute for Health and Clinical Excellence 2011) | 52 |
| G27.. | Hypertension resistant to drug therapy | 37 |
| G28.. | Stage 2 hypertension (NICE - National Institute for Health and Clinical Excellence 2011) | 130 |
| G2y.. | Other specified hypertensive disease | 813 |
| G2z.. | Hypertensive disease NOS | 13663 |
| Gyu2. | [X]Hypertensive diseases | 111 |
| Gyu20 | [X]Other secondary hypertension | <5 |

### ICD 9 and 10 codes:

| code | desc | total\_n |
| --- | --- | --- |
| 401 | Essential hypertension | 0 |
| 4010 | Malignant essential hypertension | <5 |
| 4011 | Benign essential hypertension | 28 |
| 4019 | Essential hypertension not specified | 2404 |
| 402 | Hypertensive heart disease | 0 |
| 4020 | Malignant hypertensive heart disease | 0 |
| 4021 | Benign hypertensive heart disease | <5 |
| 4029 | Hypertensive heart disease not specified | 766 |
| 405 | Secondary hypertension | 0 |
| 4050 | Specified as malignant | 0 |
| 4051 | Specified as benign | 0 |
| 4059 | Not specified as malignant or benign | 0 |
| I10 | Essential (primary) hypertension | 28516 |
| I10- | NA | <5 |
| I10# | NA | <5 |
| I10. | NA | 10 |
| I100 | NA | <5 |
| I101 | NA | <5 |
| I10X | NA | 482709 |
| I11 | Hypertensive heart disease | <5 |
| I110 | Hypertensive heart disease with (congestive) heart failure | 3283 |
| I119 | Hypertensive heart disease without (congestive) heart failure | 6091 |
| I11X | NA | <5 |
| I13 | Hypertensive heart and renal disease | <5 |
| I130 | Hypertensive heart and renal disease with (congestive) heart failure | 164 |
| I131 | Hypertensive heart and renal disease with renal failure | 131 |
| I132 | Hypertensive heart and renal disease with both (congestive) heart failure and renal failure | 577 |
| I139 | Hypertensive heart and renal disease unspecified | 158 |
| I15 | Secondary hypertension | 0 |
| I150 | Renovascular hypertension | 128 |
| I151 | Hypertension secondary to other renal disorders | 94 |
| I152 | Hypertension secondary to endocrine disorders | 12 |
| I158 | Other secondary hypertension | 67 |
| I159 | Secondary hypertension unspecified | 121 |

## Descriptives

658511 people had at least one diagnostic code in at least one of the datasets. 485638 people had a code in hospital admissions data, 28058 in mortality data and 539232 in primary care data. The following figure shows the year of the first code that was found for any person classified positive using (a) all codes combined, (b) only codes from hospital admissions data, (c) only codes from the mortality data and (d) only codes from primary care data.
