# Supplementary material for: The Secure Anonymised Information Linkage databank Dementia e-cohort (SAIL-DeC)
Source: Int J Popul Data Sci. 2020 Feb 25;5(1):1121. doi: 10.23889/ijpds.v5i1.1121 (PMC7473277; doi:10.23889/ijpds.v5i1.1121)
Supplement: Supplementary Material [file ijpds-05-01-1121-s001.zip › Supplementary Appendix 19.html]

Event tables


# Event tables

### *Hypothyroidism*

#### *January 2019*

## Code selection

We have selected codes based on QOF Business rules v24 https://www.pcc-cic.org.uk/article/qof-business-rules-v240 in conjunction with the WHO ICD 10 browser (apps.who.int/classifications/icd10/browse/2010/en) and the NHS Read Code Browser (https://isd.digital.nhs.uk/trud3/user/guest/group/0/home). We have deliberately included codes with obvious `misspelling’ (for example having a dot where none should be) or ICD 10 codes ending with ‘X’.

All codes that were selected for classification and the total number of people with at least one of the codes are displayed in the following tables. Please be aware that frequency counts of Read V2 codes in the table do not reflect the hierarchical nature of Read V2 coding (for example, counts of E01.. do not include E011.).

### Read V2 codes:

| code | desc | total\_n |
| --- | --- | --- |
| C03.. | Congenital hypothyroidism | 349 |
| C030. | Pendred’s syndrome | 5 |
| C031. | Goitrous cretin | <5 |
| C03y. | Other specified congenital hypothyroidism | <5 |
| C03y0 | Congenital hypothyroidism with diffuse goitre | <5 |
| C03y1 | Congenital hypothyroidism without goitre | <5 |
| C03z. | Congenital hypothyroidism NOS | 35 |
| C04.. | Acquired hypothyroidism | 100727 |
| C040. | Postsurgical hypothyroidism | 1445 |
| C041. | Other postablative hypothyroidism | 46 |
| C0410 | Irradiation hypothyroidism | 359 |
| C041z | Postablative hypothyroidism NOS | 103 |
| C042. | Iodine hypothyroidism | 375 |
| C043. | Other iatrogenic hypothyroidism | 129 |
| C0430 | Hypothyroidism resulting from para-aminosalicylic acid | 0 |
| C0431 | Hypothyroidism resulting from phenylbutazone | 0 |
| C0432 | Hypothyroidism resulting from resorcinol | 0 |
| C043z | Iatrogenic hypothyroidism NOS | 193 |
| C044. | Postinfectious hypothyroidism | <5 |
| C045. | Acquired atrophy of thyroid | 24 |
| C046. | Autoimmune myxoedema | 288 |
| C047. | Subclinical hypothyroidism | 2628 |
| C04y. | Other acquired hypothyroidism | 2918 |
| C04z. | Hypothyroidism NOS | 11266 |
| C04z0 | Premature puberty due to hypothyroidism | 0 |
| C04z1 | Myxoedema coma | <5 |

### ICD 9 and 10 codes:

| code | desc | total\_n |
| --- | --- | --- |
| 244 | Acquired hypothyroidism | 0 |
| 2440 | Postsurgical hypothyroidism | <5 |
| 2441 | Other postablative hypothyroidism | 0 |
| 2442 | Iodine hypothyroidism | 0 |
| 2443 | Other iatrogenic hypothyroidism | 0 |
| 2448 | Other | 0 |
| 2449 | Unspecified hypothyroidism | 190 |
| E02 | Subclinical iodine-deficiency hypothyroidism | <5 |
| E02X | NA | 357 |
| E03 | Other hypothyroidism | <5 |
| E03. | NA | 6 |
| E030 | Congenital hypothyroidism with diffuse goitre | 107 |
| E031 | Congenital hypothyroidism without goitre | 88 |
| E032 | Hypothyroidism due to medicaments and other exogenous substances | 372 |
| E033 | Postinfectious hypothyroidism | <5 |
| E034 | Atrophy of thyroid (acquired) | 21 |
| E035 | Myxoedema coma | 46 |
| E038 | Other specified hypothyroidism | 370 |
| E039 | Hypothyroidism unspecified | 84697 |
| E03X | NA | <5 |
| E890 | Postprocedural hypothyroidism | 6281 |
| E8902 | NA | 9 |
| E8903 | NA | 7 |
| E8909 | NA | <5 |

## Descriptives

130269 people had at least one diagnostic code in at least one of the datasets. 88138 people had a code in hospital admissions data, 1435 in mortality data and 111611 in primary care data. The following figure shows the year of the first code that was found for any person classified positive using (a) all codes combined, (b) only codes from hospital admissions data, (c) only codes from the mortality data and (d) only codes from primary care data.
