# Supplementary material for: The Secure Anonymised Information Linkage databank Dementia e-cohort (SAIL-DeC)
Source: Int J Popul Data Sci. 2020 Feb 25;5(1):1121. doi: 10.23889/ijpds.v5i1.1121 (PMC7473277; doi:10.23889/ijpds.v5i1.1121)
Supplement: Supplementary Material [file ijpds-05-01-1121-s001.zip › Supplementary Appendix 20.html]

Event tables


# Event tables

### *MND*

#### *Christian*

#### *January 2019*

## Code selection

We have selected codes based on UK Biobank algorithm for motor neurone disease (unpublished) in conjunction with the WHO ICD 10 browser (apps.who.int/classifications/icd10/browse/2010/en) and the NHS Read Code Browser (https://isd.digital.nhs.uk/trud3/user/guest/group/0/home). We have deliberately included codes with obvious `misspelling’ (for example having a dot where none should be) or ICD 10 codes ending with ‘X’. Please be aware that, in contrast to other risk factors and disease identified in the dementia cohort, we include medication codes for disease classification.

All codes that were selected for classification and the total number of people with at least one of the codes are displayed in the following tables. Please be aware that frequency counts of Read V2 codes in the table do not reflect the hierarchical nature of Read V2 coding (for example, counts of E01.. do not include E011.).

### Read V2 codes:

| code | desc | total\_n |
| --- | --- | --- |
| 7Q041 | Amyotrophic lateral sclerosis drugs Band 1 | <5 |
| F15.. | Anterior horn cell disease | 55 |
| F152. | Motor neurone disease | 1445 |
| F1520 | Amyotrophic lateral sclerosis | 47 |
| F1521 | Progressive muscular atrophy | 31 |
| F1522 | Progressive bulbar palsy | 122 |
| F1523 | Pseudobulbar palsy | 147 |
| F1524 | Primary lateral sclerosis | 57 |
| F152z | Motor neurone disease NOS | 47 |
| F15y. | Other anterior horn cell disease | 0 |
| F15z. | Anterior horn cell disease NOS | 8 |
| dx11. | Riluzole 50mg | 608 |
| dx12. | Rilutex 50mg | 44 |
| dx13. | NA | <5 |
| dx14. | NA | 15 |

### ICD 9 and 10 codes:

| code | desc | total\_n |
| --- | --- | --- |
| 3351 | Spinal muscular atrophy | <5 |
| 3352 | Motor neurone disease | 168 |
| G122 | Motor neuron disease | 2343 |

## Descriptives

2831 people had at least one diagnostic code in at least one of the datasets. 2084 people had a code in hospital admissions data, 1566 in mortality data and 1761 in primary care data. The following figure shows the year of the first code that was found for any person classified positive using (a) all codes combined, (b) only codes from hospital admissions data, (c) only codes from the mortality data and (d) only codes from primary care data.
