# Supplementary material for: The Secure Anonymised Information Linkage databank Dementia e-cohort (SAIL-DeC)
Source: Int J Popul Data Sci. 2020 Feb 25;5(1):1121. doi: 10.23889/ijpds.v5i1.1121 (PMC7473277; doi:10.23889/ijpds.v5i1.1121)
Supplement: Supplementary Material [file ijpds-05-01-1121-s001.zip › Supplementary Appendix 21.html]

Event tables


# Event tables

### *Myocardial Infarction*

#### *Christian*

#### *January 2019*

## Code selection

We have selected codes based on the UK Biobank algorithm and MI validation study (unpublished) in conjunction with the WHO ICD 10 browser (apps.who.int/classifications/icd10/browse/2010/en) and the NHS Read Code Browser (https://isd.digital.nhs.uk/trud3/user/guest/group/0/home). We have deliberately included codes with obvious `misspelling’ (for example having a dot where none should be) or ICD 10 codes ending with ‘X’.

Please be aware that we have included codes for ‘old MI’, which are associated with the sudden increase in the number of hospital codes (see figure below).

All codes that were selected for classification and the total number of people with at least one of the codes are displayed in the following tables. Please be aware that frequency counts of Read V2 codes in the table do not reflect the hierarchical nature of Read V2 coding (for example, counts of E01.. do not include E011.).

### Read V2 codes:

| code | desc | total\_n |
| --- | --- | --- |
| 323.. | ECG: myocardial infarction | 850 |
| 3233. | ECG: antero-septal infarct. | 82 |
| 3234. | ECG:posterior/inferior infarct | 96 |
| 3235. | ECG: subendocardial infarct | 34 |
| 3236. | ECG: lateral infarction | 22 |
| 323Z. | ECG: myocardial infarct NOS | 96 |
| 889A. | Diabetes mellitus insulin-glucose infusion in acute myocardial infarction | <5 |
| G30.. | Acute myocardial infarction | 70504 |
| G300. | Acute anterolateral infarction | 930 |
| G301. | Other specified anterior myocardial infarction | 910 |
| G3010 | Acute anteroapical infarction | 29 |
| G3011 | Acute anteroseptal infarction | 426 |
| G301z | Anterior myocardial infarction NOS | 1240 |
| G302. | Acute inferolateral infarction | 696 |
| G303. | Acute inferoposterior infarction | 216 |
| G304. | Posterior myocardial infarction NOS | 291 |
| G305. | Lateral myocardial infarction NOS | 195 |
| G306. | True posterior myocardial infarction | 26 |
| G307. | Acute subendocardial infarction | 1081 |
| G3070 | Acute non-Q wave infarction | 378 |
| G3071 | Acute non-ST segment elevation myocardial infarction | 14368 |
| G308. | Inferior myocardial infarction NOS | 5077 |
| G309. | Acute Q-wave infarct | 46 |
| G30B. | Acute posterolateral myocardial infarction | 30 |
| G30X. | Acute transmural myocardial infarction of unspecified site | 58 |
| G30X0 | Acute ST segment elevation myocardial infarction | 5067 |
| G30y. | Other acute myocardial infarction | 163 |
| G30y0 | Acute atrial infarction | 28 |
| G30y1 | Acute papillary muscle infarction | 0 |
| G30y2 | Acute septal infarction | 56 |
| G30yz | Other acute myocardial infarction NOS | 58 |
| G30z. | Acute myocardial infarction NOS | 3634 |
| G310. | Postmyocardial infarction syndrome | 179 |
| G31y1 | Microinfarction of heart | <5 |
| G35.. | Subsequent myocardial infarction | 181 |
| G350. | Subsequent myocardial infarction of anterior wall | 22 |
| G351. | Subsequent myocardial infarction of inferior wall | 32 |
| G353. | Subsequent myocardial infarction of other sites | <5 |
| G35X. | Subsequent myocardial infarction of unspecified site | 8 |
| G36.. | Certain current complications following acute myocardial infarction | <5 |
| G360. | Haemopericardium as current complication following acute myocardial infarction | 8 |
| G361. | Atrial septal defect as current complication following acute myocardial infarction | 5 |
| G362. | Ventricular septal defect as current complication following acute myocardial infarction | 6 |
| G363. | Rupture of cardiac wall without haemopericardium as current complication following acute myocardial infarction | <5 |
| G364. | Rupture of chordae tendinae as current complication following acute myocardial infarction | <5 |
| G365. | Rupture of papillary muscle as current complication following acute myocardial infarction | <5 |
| G366. | Thrombosis of atrium, auricular appendage, and ventricle as current complications following acute myocardial infarction | 10 |
| G38.. | Postoperative myocardial infarction | 65 |
| G380. | Postoperative transmural myocardial infarction of anterior wall | <5 |
| G381. | Postoperative transmural myocardial infarction of inferior wall | 5 |
| G384. | Postoperative subendocardial myocardial infarction | 10 |
| G38z. | Postoperative myocardial infarction, unspecified | 5 |
| G501. | Post infarction pericarditis | 32 |
| Gyu34 | [X]Acute transmural myocardial infarction of unspecified site | 5 |

### ICD 9 and 10 codes:

| code | desc | total\_n |
| --- | --- | --- |
| 410 | Acute myocardial infarction | 7942 |
| 412 | Old myocardial infarction | 44 |
| I21 | Acute myocardial infarction | <5 |
| I21. | NA | <5 |
| I210 | Acute transmural myocardial infarction of anterior wall | 12847 |
| I211 | Acute transmural myocardial infarction of inferior wall | 16380 |
| I212 | Acute transmural myocardial infarction of other sites | 2063 |
| I213 | Acute transmural myocardial infarction of unspecified site | 975 |
| I214 | Acute subendocardial myocardial infarction | 18406 |
| I219 | Acute myocardial infarction unspecified | 56338 |
| I21X | NA | 14 |
| I22 | Subsequent myocardial infarction | 0 |
| I22. | NA | <5 |
| I220 | Subsequent myocardial infarction of anterior wall | 1441 |
| I221 | Subsequent myocardial infarction of inferior wall | 1722 |
| I228 | Subsequent myocardial infarction of other sites | 2781 |
| I229 | Subsequent myocardial infarction of unspecified site | 6472 |
| I22X | NA | <5 |
| I23 | Certain current complications following acute myocardial infarction | 0 |
| I230 | Haemopericardium as current complication following acute myocardial infarction | 62 |
| I231 | Atrial septal defect as current complication following acute myocardial infarction | 57 |
| I232 | Ventricular septal defect as current complication following acute myocardial infarction | 154 |
| I233 | Rupture of cardiac wall without haemopericardium as current complication following acute myocardial infarction | 26 |
| I234 | Rupture of chordae tendineae as current complication following acute myocardial infarction | 13 |
| I235 | Rupture of papillary muscle as current complication following acute myocardial infarction | 22 |
| I236 | Thrombosis of atrium auricular appendage and ventricle as current complications following acute myocardial infarction | 60 |
| I238 | Other current complications following acute myocardial infarction | 313 |
| I241 | Dressler syndrome | 214 |
| I252 | Old myocardial infarction | 75829 |

## Descriptives

163098 people had at least one diagnostic code in at least one of the datasets. 123410 people had a code in hospital admissions data, 36330 in mortality data and 92843 in primary care data. The following figure shows the year of the first code that was found for any person classified positive using (a) all codes combined, (b) only codes from hospital admissions data, (c) only codes from the mortality data and (d) only codes from primary care data.
