# Supplementary material for: The Secure Anonymised Information Linkage databank Dementia e-cohort (SAIL-DeC)
Source: Int J Popul Data Sci. 2020 Feb 25;5(1):1121. doi: 10.23889/ijpds.v5i1.1121 (PMC7473277; doi:10.23889/ijpds.v5i1.1121)
Supplement: Supplementary Material [file ijpds-05-01-1121-s001.zip › Supplementary Appendix 22.html]

Event tables


# Event tables

### *Obesity*

#### *Christian*

#### *January 2019*

## Code selection

We have selected codes based on Doran, T et al. Effect of financial incentives on incentivised and non-incentivised clinical activities: longitudinal analysis of data from the UK Quality and Outcomes Framework. British Medical Journal. BMJ 2011; 342:d3590; Reeves, D et al. Can analyses of electronic patient records be independently and externally validated? The effect of statins on the mortality of patients with Ischaemic heart disease: a cohort study with nested case-control analysis. BMJ Open 2014; 4:e004952; Fairhurst, C et al. Exposure to sodium channel-inhibiting drugs and cancer survival: protocol for a cohort study using the QResearch primary care database. BMJ Open 2016;6:e011661; in conjunction with the WHO ICD 10 browser (apps.who.int/classifications/icd10/browse/2010/en) and the NHS Read Code Browser (https://isd.digital.nhs.uk/trud3/user/guest/group/0/home). We have deliberately included codes with obvious `misspelling’ (for example having a dot where none should be) or ICD 10 codes ending with ‘X’.

All codes that were selected for classification and the total number of people with at least one of the codes are displayed in the following tables. Please be aware that frequency counts of Read V2 codes in the table do not reflect the hierarchical nature of Read V2 coding (for example, counts of E01.. do not include E011.).

### Read V2 codes:

| code | desc | total\_n |
| --- | --- | --- |
| 22A5. | O/E - weight > 20% over ideal | 2488 |
| 22K5. | Body mass index 30+ - obesity | 49947 |
| 22K7. | Body mass index 40+ - severely obese | 5184 |
| 38Qb. | National Obesity Observatory Standard Evaluation Framework for weight management interventions - participant satisfaction with intervention | 6 |
| 66C1. | Initial obesity assessment | 773 |
| 66C2. | Follow-up obesity assessment | 1088 |
| 66C4. | Has seen dietician - obesity | 728 |
| 66C5. | Treatment of obesity changed | 27 |
| 66C6. | Treatment of obesity started | 283 |
| 66C7. | Treatment of obesity stopped | 47 |
| 66Cb. | Intensive weight management programme commenced | <5 |
| 66Cc. | Intensive weight management programme ended | <5 |
| 66Cd. | Intensive weight management programme declined | 14 |
| 66CE. | Reason for obesity therapy - occupational | 25 |
| 66CL. | Risk to health associated with overweight and obesity, at no increased risk | 0 |
| 66CM. | Risk to health associated with overweight and obesity, at increased risk | <5 |
| 66CN. | Risk to health associated with overweight and obesity, at high risk | <5 |
| 66CP. | Risk to health associated with overweight and obesity, at very high risk | 26 |
| 66CQ. | Intervention for risk to health associated with overweight and obesity, general advice on healthy weight and lifestyle | 879 |
| 66CR. | Intervention for risk to health associated with overweight and obesity, advice about diet and physical activity | 100 |
| 66CS. | Intervention for risk to health associated with overweight and obesity, advice about diet and physical activity, consider drugs | 0 |
| 66CT. | Intervention for risk to health associated with overweight and obesity, advice about diet and physical activity, consider drugs, consider surgery | 0 |
| 66CW. | Unsuitable for weight management programme | 20 |
| 66CX. | Obesity multidisciplinary case review | 0 |
| 66CZ. | Obesity monitoring NOS | 941 |
| 8CT5. | Anti-obesity drug therapy discontinued | <5 |
| 8CV7. | Anti-obesity drug therapy commenced | <5 |
| 8T11. | Referral to multidisciplinary obesity clinic | 5 |
| 9hN.. | Exception reporting: obesity quality indicators | 0 |
| 9hN0. | Excepted from obesity quality indicators: patient unsuitable | 13 |
| 9hN1. | Excepted from obesity quality indicators: informed dissent | <5 |
| 9OK.. | Obesity monitoring admin. | 273 |
| 9OK1. | Attends obesity monitoring | 445 |
| 9OK2. | Refuses obesity monitoring | 96 |
| 9OK3. | Obesity monitoring default | 28 |
| 9OK4. | Obesity monitoring 1st letter | 818 |
| 9OK5. | Obesity monitoring 2nd letter | 152 |
| 9OK6. | Obesity monitoring 3rd letter | 72 |
| 9OK7. | Obesity monitoring verbal inv. | 14 |
| 9OK8. | Obesity monitor phone invite | 25 |
| 9OK9. | Obesity monitoring deleted | 0 |
| 9OKA. | Obesity monitoring check done | 1286 |
| 9OKZ. | Obesity monitoring admin.NOS | 7 |
| C380. | Obesity | 42303 |
| C3800 | Obesity due to excess calories | 99 |
| C3801 | Drug-induced obesity | 9 |
| C3802 | Extreme obesity with alveolar hypoventilation | 77 |
| C3803 | Morbid obesity | 1986 |
| C3804 | Central obesity | 555 |
| C3805 | Generalised obesity | 112 |
| C3806 | Adult-onset obesity | 0 |
| C3807 | Lifelong obesity | 0 |
| C3808 | Childhood obesity | 0 |
| C38y0 | Pickwickian syndrome | 271 |
| C38z0 | Simple obesity NOS | 363 |
| ZV653 | [V]Dietary surveillance and counselling | 6804 |

### ICD 9 and 10 codes:

| code | desc | total\_n |
| --- | --- | --- |
| 2780 | Obesity | 104 |
| E66 | Obesity | 0 |
| E660 | Obesity due to excess calories | 110 |
| E661 | Drug-induced obesity | 15 |
| E662 | Extreme obesity with alveolar hypoventilation | 857 |
| E668 | Other obesity | 4646 |
| E669 | Obesity unspecified | 35498 |
| E66X | NA | <5 |

## Descriptives

120504 people had at least one diagnostic code in at least one of the datasets. 37689 people had a code in hospital admissions data, 1076 in mortality data and 97673 in primary care data. The following figure shows the year of the first code that was found for any person classified positive using (a) all codes combined, (b) only codes from hospital admissions data, (c) only codes from the mortality data and (d) only codes from primary care data.
