# Supplementary material for: The Secure Anonymised Information Linkage databank Dementia e-cohort (SAIL-DeC)
Source: Int J Popul Data Sci. 2020 Feb 25;5(1):1121. doi: 10.23889/ijpds.v5i1.1121 (PMC7473277; doi:10.23889/ijpds.v5i1.1121)
Supplement: Supplementary Material [file ijpds-05-01-1121-s001.zip › Supplementary Appendix 23.html]

Event tables


# Event tables

### *Osteoporosis*

#### *Christian*

#### *January 2019*

## Code selection

We have selected codes based on QOF Business rules v24 https://www.pcc-cic.org.uk/article/qof-business-rules-v240 in conjunction with the WHO ICD 10 browser (apps.who.int/classifications/icd10/browse/2010/en) and the NHS Read Code Browser (https://isd.digital.nhs.uk/trud3/user/guest/group/0/home). However, the following codes were removed from the list as they were not present on the NHS Read Code Browser: 7230A (OSTEOPOROSIS); 7230B (OSTEOPOROSIS SENILIS); 7230D (VERTEBRAL OSTEOPOROSIS); 7230PM (OSTEOPOROSIS POSTMENOPAUSAL); 7230PT (OSTEOPOROSIS POST-TRAUMATIC). We have deliberately included codes with obvious `misspelling’ (for example having a dot where none should be) or ICD 10 codes ending with ‘X’.

All codes that were selected for classification and the total number of people with at least one of the codes are displayed in the following tables. Please be aware that frequency counts of Read V2 codes in the table do not reflect the hierarchical nature of Read V2 coding (for example, counts of E01.. do not include E011.).

### Read V2 codes:

| code | desc | total\_n |
| --- | --- | --- |
| 58E4. | Forearm DXA scan result osteoporotic | 1090 |
| 58E8. | Heel DXA scan T score | 245 |
| 58EA. | Heel DXA scan result osteoporotic | 174 |
| 58EE. | Hip DXA scan T score | 10008 |
| 58EG. | Hip DXA scan result osteoporotic | 2985 |
| 58EK. | Lumbar spine DXA scan T score | 11748 |
| 58EM. | Lumbar DXA scan result osteoporotic | 4622 |
| 58ES. | Femoral neck DEXA scan T score | 10510 |
| 58EV. | Femoral neck DEXA scan result osteoporotic | 3225 |
| N330. | Osteoporosis | 82449 |
| N3300 | Osteoporosis, unspecified | 892 |
| N3301 | Senile osteoporosis | 342 |
| N3302 | Postmenopausal osteoporosis | 562 |
| N3303 | Idiopathic osteoporosis | 76 |
| N3304 | Dissuse osteoporosis | 23 |
| N3305 | Drug-induced osteoporosis | 173 |
| N3306 | Postoophorectomy osteoporosis | 9 |
| N3307 | Postsurgical malabsorption osteoporosis | <5 |
| N3308 | Localized osteoporosis - Lequesne | 5 |
| N3309 | Osteoporosis in multiple myelomatosis | 5 |
| N330A | Osteoporosis in endocrine disorders | 5 |
| N330B | Vertebral osteoporosis | 1141 |
| N330C | Osteoporosis localized to spine | 318 |
| N330D | Osteoporosis due to corticosteroids | 30 |
| N330z | Osteoporosis NOS | 1754 |
| N3312 | Postoophorectomy osteoporosis with pathological fracture | <5 |
| N3313 | Osteoporosis of disuse with pathological fracture | 12 |
| N3314 | Postsurgical malabsorption osteoporosis with pathological fracture | <5 |
| N3315 | Drug-induced osteoporosis with pathological fracture | 15 |
| N3316 | Idiopathic osteoporosis with pathological fracture | 31 |
| N3317 | Fracture of bone in neoplastic disease | 9 |
| N3318 | Osteoporosis + pathological fracture lumbar vertebrae | 518 |
| N3319 | Osteoporosis + pathological fracture thoracic vertebrae | 580 |
| N331A | Osteoporosis + pathological fracture cervical vertebrae | 12 |
| N331B | Postmenopausal osteoporosis with pathological fracture | 65 |
| N331M | Fragility fracture due to unspecified osteoporosis | 1024 |
| N331N | Fragility fracture | 14088 |
| NyuB0 | [X]Other osteoporosis with pathological fracture | 34 |
| NyuB1 | [X]Other osteoporosis | 42 |
| NyuB2 | [X]Osteoporosis in other disorders classified elsewhere | 6 |
| NyuB8 | [X]Unspecified osteoporosis with pathological fracture | 98 |
| 58EE0 | NA | <5 |
| 58EK0 | NA | 6 |
| 58ES0 | NA | <5 |

### ICD 9 and 10 codes:

ICD 10 codes for Osteoporosis (M80, M81 and M82) can have a 5th digit indicative of the affected site (0=Multiple sites; 1=Shoulder region; 2=Upper arm; 3= Forearm; 4=Hand; 5=Pelvic region and thigh; 6=Lower leg; 7=Ankle and foot; 8=Other; 9=Site unspecified). Please see your favorit ICD 10 documentation for futher information.

Please be aware that there is an unknown number of codes starting with M8 and M9 which are not ICD 10 codes but (indistinguishable) cancer morphology codes (ICD 0). More information on the WIKI (within SAIL) http://xwiki.chi.swan.ac.uk/xwiki/bin/view/Question+and+Answer+Forum/Osteoporosis+or+Cancer%3F

and (outside of SAIL)

http://www.classificationstandards.wales.nhs.uk/wcs01-icd-10-classification-neoplasm-mor

We have deleted all 6-digit codes (other than those where the 6th digit was an A or D) and those that do not exist in the ICD 10 browser (for example M80.7). However, there will still be a (hopefully) low number of false positives.

| code | desc | total\_n |
| --- | --- | --- |
| 7330 | Osteoporosis | 243 |
| M80 | Osteoporosis with pathological fracture | 0 |
| M800 | Postmenopausal osteoporosis with pathological fracture | 310 |
| M801 | Postoophorectomy osteoporosis with pathological fracture | 180 |
| M802 | Osteoporosis of disuse with pathological fracture | 61 |
| M803 | Postsurgical malabsorption osteoporosis with pathological fracture | <5 |
| M804 | Drug-induced osteoporosis with pathological fracture | 297 |
| M805 | Idiopathic osteoporosis with pathological fracture | 94 |
| M808 | Other osteoporosis with pathological fracture | 1635 |
| M809 | Unspecified osteoporosis with pathological fracture | 9394 |
| M81 | Osteoporosis without pathological fracture | 0 |
| M81. | NA | <5 |
| M810 | Postmenopausal osteoporosis | 239 |
| M811 | Postoophorectomy osteoporosis | 17 |
| M812 | Osteoporosis of disuse | 128 |
| M813 | Postsurgical malabsorption osteoporosis | 184 |
| M814 | Drug-induced osteoporosis | 999 |
| M815 | Idiopathic osteoporosis | 32 |
| M816 | Localized osteoporosis [Lequesne] | 56 |
| M818 | Other osteoporosis | 407 |
| M819 | Osteoporosis unspecified | 63022 |
| M820 | Osteoporosis in multiple myelomatosis | 0 |
| M821 | Osteoporosis in endocrine disorders | 0 |
| M828 | Osteoporosis in other diseases classified elsewhere | 0 |

## Descriptives

131647 people had at least one diagnostic code in at least one of the datasets. 69409 people had a code in hospital admissions data, 1730 in mortality data and 100449 in primary care data. The following figure shows the year of the first code that was found for any person classified positive using (a) all codes combined, (b) only codes from hospital admissions data, (c) only codes from the mortality data and (d) only codes from primary care data.
