# Supplementary material for: The Secure Anonymised Information Linkage databank Dementia e-cohort (SAIL-DeC)
Source: Int J Popul Data Sci. 2020 Feb 25;5(1):1121. doi: 10.23889/ijpds.v5i1.1121 (PMC7473277; doi:10.23889/ijpds.v5i1.1121)
Supplement: Supplementary Material [file ijpds-05-01-1121-s001.zip › Supplementary Appendix 24.html]

Event tables


# Event tables

### *Parkinson*

#### *Christian*

#### *January 2019*

## Code selection

We have selected codes based on Codes taken from the UK Biobank algorithm and Parkinson’s disease validation study in conjunction with the WHO ICD 10 browser (apps.who.int/classifications/icd10/browse/2010/en) and the NHS Read Code Browser (https://isd.digital.nhs.uk/trud3/user/guest/group/0/home). We have deliberately included codes with obvious `misspelling’ (for example having a dot where none should be) or ICD 10 codes ending with ‘X’.

All codes that were selected for classification and the total number of people with at least one of the codes are displayed in the following tables. Please be aware that frequency counts of Read V2 codes in the table do not reflect the hierarchical nature of Read V2 coding (for example, counts of E01.. do not include E011.).

### Read V2 codes:

| code | desc | total\_n |
| --- | --- | --- |
| 147F. | History of Parkinson’s disease | 239 |
| F12.. | Parkinson’s disease | 16508 |
| F120. | Paralysis agitans | 1409 |
| F12z. | Parkinson’s disease NOS | 1121 |

### ICD 9 and 10 codes:

| code | desc | total\_n |
| --- | --- | --- |
| 3320 | Paralysis agitans | 1016 |
| G20 | Parkinson disease | 6810 |
| G20. | NA | 7 |
| G20+ | NA | 6 |
| G200 | NA | 304 |
| G204 | NA | <5 |
| G20D | NA | 56 |
| G20X | NA | 21112 |

## Descriptives

27483 people had at least one diagnostic code in at least one of the datasets. 21260 people had a code in hospital admissions data, 7565 in mortality data and 18067 in primary care data. The following figure shows the year of the first code that was found for any person classified positive using (a) all codes combined, (b) only codes from hospital admissions data, (c) only codes from the mortality data and (d) only codes from primary care data.
