# Supplementary material for: The Secure Anonymised Information Linkage databank Dementia e-cohort (SAIL-DeC)
Source: Int J Popul Data Sci. 2020 Feb 25;5(1):1121. doi: 10.23889/ijpds.v5i1.1121 (PMC7473277; doi:10.23889/ijpds.v5i1.1121)
Supplement: Supplementary Material [file ijpds-05-01-1121-s001.zip › Supplementary Appendix 25.html]

Event tables


# Event tables

### *Peripheral arterial disease events*

#### *Christian*

#### *January 2019*

## Code selection

We have selected codes based on QOF Business rules v24 https://www.pcc-cic.org.uk/article/qof-business-rules-v240 in conjunction with the WHO ICD 10 browser (apps.who.int/classifications/icd10/browse/2010/en) and the NHS Read Code Browser (https://isd.digital.nhs.uk/trud3/user/guest/group/0/home). In line with current practice, the term peripheral arterial disease is used to describe a narrowing or occlusion of the peripheral arteries, affecting the blood supply to the lower limb. We have deliberately included codes with obvious `misspelling’ (for example having a dot where none should be) or ICD 10 codes ending with ‘X’.

All codes that were selected for classification and the total number of people with at least one of the codes are displayed in the following tables. Please be aware that frequency counts of Read V2 codes in the table do not reflect the hierarchical nature of Read V2 coding (for example, counts of E01.. do not include E011.).

### Read V2 codes:

| code | desc | total\_n |
| --- | --- | --- |
| G73.. | Other peripheral vascular disease | 9273 |
| G73z. | Peripheral vascular disease NOS | 19334 |
| G73z0 | Intermittent claudication | 24192 |
| G73zz | Peripheral vascular disease NOS | 5972 |
| Gyu74 | [X]Other specified peripheral vascular diseases | 6 |

### ICD 9 and 10 codes:

| code | desc | total\_n |
| --- | --- | --- |
| 4439 | Unspecified | 1117 |
| I739 | Peripheral vascular disease unspecified | 40861 |

## Descriptives

68346 people had at least one diagnostic code in at least one of the datasets. 38257 people had a code in hospital admissions data, 7553 in mortality data and 46123 in primary care data. The following figure shows the year of the first code that was found for any person classified positive using (a) all codes combined, (b) only codes from hospital admissions data, (c) only codes from the mortality data and (d) only codes from primary care data.
