# Supplementary material for: The Secure Anonymised Information Linkage databank Dementia e-cohort (SAIL-DeC)
Source: Int J Popul Data Sci. 2020 Feb 25;5(1):1121. doi: 10.23889/ijpds.v5i1.1121 (PMC7473277; doi:10.23889/ijpds.v5i1.1121)
Supplement: Supplementary Material [file ijpds-05-01-1121-s001.zip › Supplementary Appendix 26.html]

Event tables


# Event tables

### *Rheumatoid Arthritis*

#### *Christian*

#### *January 2019*

## Code selection

We have selected codes based on Nicholson, A et al. Optimising Use of Electronic Health Records to Describe the Presentation of Rheumatoid Arthritis in Primary Care: A Strategy for Developing Code Lists. PLoS ONE 2013;8(2):e54878 (https://clinicalcodes.rss.mhs.man.ac.uk/medcodes/article/13/) in conjunction with the WHO ICD 10 browser (apps.who.int/classifications/icd10/browse/2010/en) and the NHS Read Code Browser (https://isd.digital.nhs.uk/trud3/user/guest/group/0/home). We have deliberately included codes with obvious `misspelling’ (for example having a dot where none should be) or ICD 10 codes ending with ‘X’.

All codes that were selected for classification and the total number of people with at least one of the codes are displayed in the following tables. Please be aware that frequency counts of Read V2 codes in the table do not reflect the hierarchical nature of Read V2 coding (for example, counts of E01.. do not include E011.).

### Read V2 codes:

| code | desc | total\_n |
| --- | --- | --- |
| 2G25. | O/E - hands - ulnar deviation | 103 |
| 2G27. | O/E-hands-rheumatoid spindling | 58 |
| F3712 | Polyneuropathy in rheumatoid arthritis | 17 |
| F3964 | Myopathy due to rheumatoid arthritis | 10 |
| G5y8. | Rheumatoid myocarditis | <5 |
| H570. | Rheumatoid lung | 73 |
| N04.. | Rheumatoid arthritis and other inflammatory polyarthropathies | 1383 |
| N040. | Rheumatoid arthritis | 24991 |
| N0400 | Rheumatoid arthritis of cervical spine | 26 |
| N0401 | Other rheumatoid arthritis of spine | 9 |
| N0402 | Rheumatoid arthritis of shoulder | 37 |
| N0403 | Rheumatoid arthritis of sternoclavicular joint | <5 |
| N0404 | Rheumatoid arthritis of acromioclavicular joint | <5 |
| N0405 | Rheumatoid arthritis of elbow | 28 |
| N0406 | Rheumatoid arthritis of distal radio-ulnar joint | <5 |
| N0407 | Rheumatoid arthritis of wrist | 52 |
| N0408 | Rheumatoid arthritis of metacarpophalangeal joint | 64 |
| N0409 | Rheumatoid arthritis of proximal interphalangeal joint of finger | 28 |
| N040A | Rheumatoid arthritis of distal interphalangeal joint of finger | 17 |
| N040B | Rheumatoid arthritis of hip | 19 |
| N040C | Rheumatoid arthritis of sacro-iliac joint | <5 |
| N040D | Rheumatoid arthritis of knee | 99 |
| N040E | Rheumatoid arthritis of tibio-fibular joint | <5 |
| N040F | Rheumatoid arthritis of ankle | 27 |
| N040G | Rheumatoid arthritis of subtalar joint | 5 |
| N040H | Rheumatoid arthritis of talonavicular joint | 5 |
| N040J | Rheumatoid arthritis of other tarsal joint | 6 |
| N040K | Rheumatoid arthritis of 1st metatarsophalangeal joint | 9 |
| N040L | Rheumatoid arthritis of lesser metatarsophalangeal joint | 7 |
| N040M | Rheumatoid arthritis of interphalangeal joint of toe | <5 |
| N040N | Rheumatoid vasculitis | 52 |
| N040P | Seronegative rheumatoid arthritis | 1205 |
| N040Q | Rheumatoid bursitis | 16 |
| N040R | Rheumatoid nodule | 130 |
| N040S | Rheumatoid arthritis - multiple joint | 296 |
| N040T | Flare of rheumatoid arthritis | 1009 |
| N041. | Felty’s syndrome | 111 |
| N042. | Other rheumatoid arthropathy with visceral or systemic involvement | 5 |
| N0420 | Rheumatic carditis | 112 |
| N0421 | Rheumatoid lung disease | 26 |
| N0422 | Rheumatoid nodule | 340 |
| N042z | Rheumatoid arthropathy with visceral or systemic involvement NOS | 9 |
| N043. | Juvenile rheumatoid arthritis - Still’s disease | 78 |
| N0430 | Juvenile rheumatoid arthropathy unspecified | <5 |
| N0431 | Acute polyarticular juvenile rheumatoid arthritis | <5 |
| N0432 | Pauciarticular juvenile rheumatoid arthritis | 5 |
| N0433 | Monarticular juvenile rheumatoid arthritis | <5 |
| N043z | Juvenile rheumatoid arthritis NOS | 5 |
| N044. | Chronic post-rheumatic arthropathy | 510 |
| N045. | Other juvenile arthritis | 8 |
| N0450 | Juvenile ankylosing spondylitis | <5 |
| N0451 | Juvenile seronegative polyarthritis | 7 |
| N0452 | Juvenile arthritis in psoriasis | 11 |
| N0453 | Juvenile arthritis in Crohn’s disease | <5 |
| N0454 | Juvenile arthritis in ulcerative colitis | 0 |
| N0455 | Juvenile rheumatoid arthritis | 13 |
| N0456 | Pauciarticular onset juvenile chronic arthritis | 0 |
| N047. | Seropositive errosive rheumatoid arthritis | 198 |
| N04X. | Seropositive rheumatoid arthritis, unspecified | 959 |
| N04z. | Inflammatory polyarthropathy NOS | 697 |
| Nyu11 | [X]Other seropositive rheumatoid arthritis | 17 |
| Nyu12 | [X]Other specified rheumatoid arthritis | 5 |
| Nyu15 | [X]Other juvenile arthritis | 0 |
| Nyu1G | [X]Seropositive rheumatoid arthritis, unspecified | 26 |

### ICD 9 and 10 codes:

ICD 10 codes for Rheumatoid Arthritis (M05 and M06) can have a 5th digit indicative of the affected site (0=Multiple sites; 1=Shoulder region; 2=Upper arm; 3= Forearm; 4=Hand; 5=Pelvic region and thigh; 6=Lower leg; 7=Ankle and foot; 8=Other; 9=Site unspecified). Please see ICD 10 documentation for futher information.

| code | desc | total\_n |
| --- | --- | --- |
| 7140 | Rheumatoid arthritis | 511 |
| 7141 | Felty s syndrome | <5 |
| 7142 | Other rheumatoid arthritis with visceral or system | <5 |
| 7148 | Other | 5 |
| M05 | Seropositive rheumatoid arthritis | 0 |
| M05. | NA | 6 |
| M050 | Felty syndrome | 144 |
| M051 | Rheumatoid lung disease | 332 |
| M052 | Rheumatoid vasculitis | 196 |
| M053 | Rheumatoid arthritis with involvement of other organs and systems | 68 |
| M058 | Other seropositive rheumatoid arthritis | 257 |
| M059 | Seropositive rheumatoid arthritis unspecified | 3046 |
| M06. | NA | <5 |
| M060 | Seronegative rheumatoid arthritis | 1806 |
| M062 | Rheumatoid bursitis | 28 |
| M063 | Rheumatoid nodule | 479 |
| M064 | Inflammatory polyarthropathy | 309 |
| M066 | NA | <5 |
| M067 | NA | <5 |
| M068 | Other specified rheumatoid arthritis | 360 |
| M069 | Rheumatoid arthritis unspecified | 30593 |

## Descriptives

44151 people had at least one diagnostic code in at least one of the datasets. 32197 people had a code in hospital admissions data, 2655 in mortality data and 28144 in primary care data. The following figure shows the year of the first code that was found for any person classified positive using (a) all codes combined, (b) only codes from hospital admissions data, (c) only codes from the mortality data and (d) only codes from primary care data.
