# Supplementary material for: The Secure Anonymised Information Linkage databank Dementia e-cohort (SAIL-DeC)
Source: Int J Popul Data Sci. 2020 Feb 25;5(1):1121. doi: 10.23889/ijpds.v5i1.1121 (PMC7473277; doi:10.23889/ijpds.v5i1.1121)
Supplement: Supplementary Material [file ijpds-05-01-1121-s001.zip › Supplementary Appendix 27.html]

Event tables


# Event tables

### *Ever smoking*

#### *Christian*

#### *January 2019*

## Code selection

We have selected codes for either based on Stocks, J et al. Examining variations in prescribing safety in UK general practice: a cross-sectional study using the Clinical Practice Research Datalink. BMJ 2015;351:h5501; Fairhurst, C et al. Sodium channel-inhibiting drugs and cancer survival: protocol for a cohort study using the CPRD primary care database. BMJ Open 2016;6:e011661; Springate, D et al. Can analyses of electronic patient records be independently and externally validated? Study 2: the Effect of Beta-Adrenoceptor Blocker Therapy on Cancer Survival; a Retrospective Cohort Study. BMJ Open 2015;5:e007299 in conjunction with the WHO ICD 10 browser (apps.who.int/classifications/icd10/browse/2010/en) and the NHS Read Code Browser (https://isd.digital.nhs.uk/trud3/user/guest/group/0/home). We have deliberately included codes with obvious `misspelling’ (for example having a dot where none should be) or ICD 10 codes ending with ‘X’.

Note: There are no ICD-10 codes for smoking - they only appear in the primary care data. Smoking was added to the Quality Outcomes Framework (QOF) in 2006, which may explain the sudden increase in coding around this time. Given the retrospective, observational nature of the data, we do not try to distinguish between ‘past smoking’ and ‘current smoking’.

All codes that were selected for classification and the total number of people with at least one of the codes are displayed in the following tables. Please be aware that frequency counts of Read V2 codes in the table do not reflect the hierarchical nature of Read V2 coding (for example, counts of E01.. do not include E011.).

### Read V2 codes:

| code | desc | total\_n |
| --- | --- | --- |
| 137.. | Tobacco consumption | 114320 |
| 1372. | Trivial smoker - < 1 cig/day | 22183 |
| 1373. | Light smoker - 1-9 cigs/day | 73707 |
| 1374. | Moderate smoker - 10-19 cigs/d | 105608 |
| 1375. | Heavy smoker - 20-39 cigs/day | 65980 |
| 1376. | Very heavy smoker - 40+cigs/d | 6825 |
| 1377. | Ex-trivial smoker (<1/day) | 17793 |
| 1378. | Ex-light smoker (1-9/day) | 58529 |
| 1379. | Ex-moderate smoker (10-19/day) | 101232 |
| 137a. | Pipe tobacco consumption | 2008 |
| 137A. | Ex-heavy smoker (20-39/day) | 57813 |
| 137b. | Ready to stop smoking | 5590 |
| 137B. | Ex-very heavy smoker (40+/day) | 14902 |
| 137c. | Thinking about stopping smoking | 8318 |
| 137C. | Keeps trying to stop smoking | 2132 |
| 137d. | Not interested in stopping smoking | 12264 |
| 137D. | Admitted tobacco cons untrue ? | 53 |
| 137e. | Smoking restarted | 385 |
| 137E. | Tobacco consumption unknown | 4249 |
| 137f. | Reason for restarting smoking | 28 |
| 137F. | Ex-smoker - amount unknown | 69222 |
| 137g. | Cigarette pack-years | 5520 |
| 137G. | Trying to give up smoking | 33582 |
| 137h. | Minutes from waking to first tobacco consumption | 182 |
| 137H. | Pipe smoker | 12336 |
| 137j. | Ex-cigarette smoker | 19150 |
| 137J. | Cigar smoker | 14076 |
| 137K. | Stopped smoking | 103495 |
| 137K0 | Recently stopped smoking | 1326 |
| 137l. | Ex roll-up cigarette smoker | 870 |
| 137m. | Failed attempt to stop smoking | 2710 |
| 137M. | Rolls own cigarettes | 20862 |
| 137n. | Total time smoked | 9078 |
| 137N. | Ex pipe smoker | 5082 |
| 137o. | Waterpipe tobacco consumption | 61 |
| 137O. | Ex cigar smoker | 3725 |
| 137P. | Cigarette smoker | 205518 |
| 137Q. | Smoking started | 2550 |
| 137R. | Current smoker | 125863 |
| 137S. | Ex smoker | 429585 |
| 137T. | Date ceased smoking | 23162 |
| 137V. | Smoking reduced | 1302 |
| 137X. | Cigarette consumption | 6035 |
| 137Y. | Cigar consumption | 1278 |
| 137Z. | Tobacco consumption NOS | 14715 |
| 13p.. | Smoking cessation milestones | 4779 |
| 13p0. | Negotiated date for cessation of smoking | 1617 |
| 13p1. | Smoking status at 4 weeks | 423 |
| 13p2. | Smoking status between 4 and 52 weeks | 194 |
| 13p3. | Smoking status at 52 weeks | 16 |
| 13p4. | Smoking free weeks | 657 |
| 13p5. | Smoking cessation programme start date | 824 |
| 13p50 | Practice based smoking cessation programme start date | 272 |
| 13p6. | Carbon monoxide reading at 4 weeks | 196 |
| 13p7. | Smoking status at 12 weeks | 24 |
| 13p8. | Lost to smoking cessation follow-up | 14 |
| 38DH. | Fagerstrom test for nicotine dependence | 5 |
| 67H1. | Lifestyle advice regarding smoking | 12047 |
| 67H6. | Brief intervention for smoking cessation | 4144 |
| 745H. | Smoking cessation therapy | 9664 |
| 745H0 | Nicotine replacement therapy using nicotine patches | 1704 |
| 745H1 | Nicotine replacement therapy using nicotine gum | 207 |
| 745H2 | Nicotine replacement therapy using nicotine inhalator | 557 |
| 745H3 | Nicotine replacement therapy using nicotine lozenges | 158 |
| 745H4 | Smoking cessation drug therapy | 1264 |
| 745H5 | Varenicline therapy | 8 |
| 745Hy | Other specified smoking cessation therapy | 470 |
| 745Hz | Smoking cessation therapy NOS | 819 |
| 8B2B. | Nicotine replacement therapy | 12364 |
| 8B2B0 | Issue of nicotine replacement therapy voucher | 0 |
| 8B31G | Varenicline smoking cessation therapy offered | <5 |
| 8B3f. | Nicotine replacement therapy provided free | 2941 |
| 8B3Y. | Over the counter nicotine replacement therapy | 1157 |
| 8BP3. | Nicotine replacement therapy provided by community pharmacist | 9 |
| 8CAg. | Smoking cessation advice provided by community pharmacist | 184 |
| 8CAL. | Smoking cessation advice | 342254 |
| 8CdB. | Stop smoking service opportunity signposted | 1965 |
| 8H7i. | Referral to smoking cessation advisor | 14754 |
| 8HBM. | Stop smoking face to face follow-up | 291 |
| 8HBP. | Smoking cessation 12 week follow-up | 10 |
| 8HkQ. | Referral to NHS stop smoking service | 5839 |
| 8HTK. | Referral to stop-smoking clinic | 11965 |
| 8I2I. | Nicotine replacement therapy contraindicated | <5 |
| 8I39. | Nicotine replacement therapy refused | 725 |
| 8IAj. | Smoking cessation advice declined | 20230 |
| 8IEK. | Smoking cessation programme declined | 7603 |
| 8IEM. | Smoking cessation drug therapy declined | 8779 |
| 8IEM0 | Varenicline smoking cessation therapy declined | 0 |
| 8IEo. | Referral to smoking cessation service declined | 4017 |
| 8T08. | Referral to smoking cessation service | 745 |
| 9hG.. | Exception reporting: smoking quality indicators | 626 |
| 9hG0. | Excepted from smoking quality indicators: Patient unsuitable | 5297 |
| 9hG1. | Excepted from smoking quality indicators: Informed dissent | 10264 |
| 9kc.. | Smoking cessation - enhanced services administration | 83 |
| 9kc0. | Smoking cessation monitoring template completed - enhanced services administration | <5 |
| 9kf1. | Referred for chronic obstructive pulmonary disease structured smoking assessment - enhanced services administration | <5 |
| 9kf2. | Chronic obstructive pulmonary disease structured smoking assessment declined - enhanced services administration | 10 |
| 9km.. | Ex-smoker annual review - enhanced services administration | 508 |
| 9ko.. | Current smoker annual review - enhanced services administration | 446 |
| 9N2k. | Seen by smoking cessation advisor | 4489 |
| 9N4M. | DNA - Did not attend smoking cessation clinic | 887 |
| 9Ndf. | Consent given for follow-up by smoking cessation team | 219 |
| 9Ndg. | Declined consent for follow-up by smoking cessation team | 2658 |
| 9NdV. | Consent given for follow-up evaluation after smoking cessation intervention | 6 |
| 9NdW. | Consent given for smoking cessation data sharing | 81 |
| 9NdY. | Declined consent for follow-up evaluation after smoking cessation intervention | 58 |
| 9NdZ. | Declined consent for smoking cessation data sharing | 82 |
| 9NS02 | Referral for smoking cessation service offered | 3786 |
| 9OO.. | Anti-smoking monitoring admin. | 3791 |
| 9OO1. | Attends stop smoking monitor. | 1268 |
| 9OO2. | Refuses stop smoking monitor | 1239 |
| 9OO3. | Stop smoking monitor default | 168 |
| 9OO4. | Stop smoking monitor 1st lettr | 7636 |
| 9OO5. | Stop smoking monitor 2nd lettr | 1622 |
| 9OO6. | Stop smoking monitor 3rd lettr | 658 |
| 9OO7. | Stop smoking monitor verb.inv. | 1418 |
| 9OO8. | Stop smoking monitor phone inv | 615 |
| 9OO9. | Stop smoking monitoring delete | 6 |
| 9OOA. | Stop smoking monitor.chck done | 554 |
| 9OOB. | Stop smoking invitation short message service text message | <5 |
| 9OOB0 | Stop smoking invitation first short message service text message | 49 |
| 9OOB1 | Stop smoking invitation second short message service text message | <5 |
| 9OOB2 | Stop smoking invitation third short message service text message | 5 |
| 9OOZ. | Stop smoking monitor admin.NOS | 366 |
| E023. | Nicotine withdrawal | 120 |
| E251. | Tobacco dependence | 1802 |
| E2510 | Tobacco dependence, unspecified | <5 |
| E2511 | Tobacco dependence, continuous | <5 |
| E2512 | Tobacco dependence, episodic | 0 |
| E2513 | Tobacco dependence in remission | 0 |
| E251z | Tobacco dependence NOS | <5 |
| Eu17. | [X]Mental and behavioural disorder due to use of tobacco | 0 |
| Eu170 | [X]Mental and behavioural disorders due to use of tobacco: acute intoxication | <5 |
| Eu171 | [X]Mental and behavioural disorders due to use of tobacco: harmful use | 5 |
| Eu172 | [X]Mental and behavioural disorders due to use of tobacco: dependence syndrome | 0 |
| Eu173 | [X]Mental and behavioural disorders due to use of tobacco: withdrawal state | 0 |
| Eu174 | [X]Mental and behavioural disorders due to use of tobacco: withdrawal state with delirium | 0 |
| Eu175 | [X]Mental and behavioural disorders due to use of tobacco: psychotic disorder | 0 |
| Eu176 | [X]Mental and behavioural disorders due to use of tobacco: amnesic syndrome | 0 |
| Eu177 | [X]Mental and behavioural disorders due to use of tobacco: residual and late-onset psychotic disorder | 0 |
| Eu17y | [X]Mental and behavioural disorders due to use of tobacco: other mental and behavioural disorders | 0 |
| Eu17z | [X]Mental and behavioural disorders due to use of tobacco: unspecified mental and behavioural disorder | 0 |
| H3101 | Smokers’ cough | 831 |
| J0364 | Tobacco deposit on teeth | 0 |
| J0873 | Leukokeratosis nicotina palati | 6 |
| SMC.. | Toxic effect of tobacco and nicotine | 5 |
| ZV116 | [V]Personal history of tobacco abuse | <5 |
| ZV4K0 | [V]Tobacco use | 311 |
| ZV6D8 | [V]Tobacco abuse counselling | <5 |
| 1370. | NA | <5 |
| 137H0 | NA | 31 |
| 137j0 | NA | 98 |
| 137J0 | NA | 34 |
| 137M0 | NA | <5 |
| 137n0 | NA | <5 |
| 137N0 | NA | <5 |
| 137O0 | NA | <5 |
| 137P0 | NA | 17 |
| 137R0 | NA | <5 |
| 137s. | NA | 262 |
| 137S0 | NA | 5 |
| 137T0 | NA | <5 |
| 137Y0 | NA | <5 |

```

## Descriptive statistics

714588 people had at least one diagnostic code in the primary care data. The following figure shows the year of the first code that was found for any person classified positive.
