# Supplementary material for: The Secure Anonymised Information Linkage databank Dementia e-cohort (SAIL-DeC)
Source: Int J Popul Data Sci. 2020 Feb 25;5(1):1121. doi: 10.23889/ijpds.v5i1.1121 (PMC7473277; doi:10.23889/ijpds.v5i1.1121)
Supplement: Supplementary Material [file ijpds-05-01-1121-s001.zip › Supplementary Appendix 28.html]

Event tables


# Event tables

### *Stroke*

#### *Christian*

#### *January 2019*

## Code selection

We have selected codes based on the UK Biobank algorithm and stroke validation study (unpublished) in conjunction with the WHO ICD 10 browser (apps.who.int/classifications/icd10/browse/2010/en) and the NHS Read Code Browser (https://isd.digital.nhs.uk/trud3/user/guest/group/0/home). We have deliberately included codes with obvious `misspelling’ (for example having a dot where none should be) or ICD 10 codes ending with ‘X’.

All codes that were selected for classification and the total number of people with at least one of the codes are displayed in the following tables. Please be aware that frequency counts of Read V2 codes in the table do not reflect the hierarchical nature of Read V2 coding (for example, counts of E01.. do not include E011.).

### Read V2 codes:

| code | desc | total\_n |
| --- | --- | --- |
| G60.. | Subarachnoid haemorrhage | 4689 |
| G600. | Ruptured berry aneurysm | 78 |
| G601. | Subarachnoid haemorrhage from carotid siphon and bifurcation | <5 |
| G602. | Subarachnoid haemorrhage from middle cerebral artery | 67 |
| G603. | Subarachnoid haemorrhage from anterior communicating artery | 28 |
| G604. | Subarachnoid haemorrhage from posterior communicating artery | 31 |
| G605. | Subarachnoid haemorrhage from basilar artery | 10 |
| G606. | Subarachnoid haemorrhage from vertebral artery | <5 |
| G60X. | Subarachnoid haemorrhage from intracranial artery, unspecified | 49 |
| G60z. | Subarachnoid haemorrhage NOS | 124 |
| G61.. | Intracerebral haemorrhage | 5476 |
| G610. | Cortical haemorrhage | 26 |
| G611. | Internal capsule haemorrhage | 71 |
| G612. | Basal nucleus haemorrhage | 26 |
| G613. | Cerebellar haemorrhage | 395 |
| G614. | Pontine haemorrhage | 64 |
| G615. | Bulbar haemorrhage | 10 |
| G616. | External capsule haemorrhage | 5 |
| G617. | Intracerebral haemorrhage, intraventricular | 61 |
| G618. | Intracerebral haemorrhage, multiple localized | 13 |
| G619. | Lobar cerebral haemorrhage | 47 |
| G61X. | Intracerebral haemorrhage in hemisphere, unspecified | 27 |
| G61X0 | Left sided intracerebral haemorrhage, unspecified | 39 |
| G61X1 | Right sided intracerebral haemorrhage, unspecified | 42 |
| G61z. | Intracerebral haemorrhage NOS | 208 |
| G63y. | Other precerebral artery occlusion | 7 |
| G63y0 | Cerebral infarct due to thrombosis of precerebral arteries | 241 |
| G63y1 | Cerebral infarction due to embolism of precerebral arteries | 58 |
| G64.. | Cerebral arterial occlusion | 11929 |
| G640. | Cerebral thrombosis | 563 |
| G6400 | Cerebral infarction due to thrombosis of cerebral arteries | 127 |
| G641. | Cerebral embolism | 275 |
| G6410 | Cerebral infarction due to embolism of cerebral arteries | 36 |
| G64z. | Cerebral infarction NOS | 7655 |
| G64z0 | Brainstem infarction | 155 |
| G64z1 | Wallenberg syndrome | 86 |
| G64z2 | Left sided cerebral infarction | 861 |
| G64z3 | Right sided cerebral infarction | 804 |
| G64z4 | Infarction of basal ganglia | 415 |
| G66.. | Stroke and cerebrovascular accident unspecified | 62575 |
| G660. | Middle cerebral artery syndrome | 182 |
| G661. | Anterior cerebral artery syndrome | 35 |
| G662. | Posterior cerebral artery syndrome | 88 |
| G663. | Brain stem stroke syndrome | 264 |
| G664. | Cerebellar stroke syndrome | 352 |
| G665. | Pure motor lacunar syndrome | 121 |
| G666. | Pure sensory lacunar syndrome | 41 |
| G667. | Left sided CVA | 2766 |
| G668. | Right sided CVA | 2476 |
| G669. | Cerebral palsy, not congenital or infantile, acute | 19 |
| G6760 | Cerebral infarction due to cerebral venous thrombosis, nonpyogenic | 36 |
| G6W.. | Cerebral infarction due to unspecified occlusion or stenosis of precerebral arteries | 96 |
| G6X.. | Cerebral infarction due to unspecified occlusion or stenosis of cerebral arteries | 362 |
| Gyu60 | [X]Subarachnoid haemorrhage from other intracranial arteries | <5 |
| Gyu61 | [X]Other subarachnoid haemorrhage | 7 |
| Gyu62 | [X]Other intracerebral haemorrhage | 28 |
| Gyu63 | [X]Cerebral infarction due to unspecified occlusion or stenosis of cerebral arteries | 27 |
| Gyu64 | [X]Other cerebral infarction | 294 |
| Gyu6E | [X]Subarachnoid haemorrhage from intracranial artery, unspecified | <5 |
| Gyu6F | [X]Intracerebral haemorrhage in hemisphere, unspecified | 12 |
| Gyu6G | [X]Cerebral infarction due to unspecified occlusion or stenosis of precerebral arteries | 9 |

### ICD 9 and 10 codes:

| code | desc | total\_n |
| --- | --- | --- |
| 430 | Subarachnoid haemorrhage | 317 |
| 4300 | NA | 316 |
| 4301 | NA | 24 |
| 4302 | NA | 54 |
| 430A | Subarachnoid haemorrhage | 0 |
| 431 | Intracerebral haemorrhage | 784 |
| 433 | Occlusion and stenosis of precerebral arteries | 0 |
| 4330 | Basilar artery | <5 |
| 4331 | Carotid artery | 30 |
| 4332 | Vertebral artery | <5 |
| 4333 | Multiple and bilateral | <5 |
| 4338 | Other | <5 |
| 4339 | Unspecified | 0 |
| 436 | Acute but ill-defined cerebrovascular disease | 6258 |
| I60 | Subarachnoid haemorrhage | 0 |
| I600 | Subarachnoid haemorrhage from carotid siphon and bifurcation | 72 |
| I601 | Subarachnoid haemorrhage from middle cerebral artery | 392 |
| I602 | Subarachnoid haemorrhage from anterior communicating artery | 441 |
| I603 | Subarachnoid haemorrhage from posterior communicating artery | 320 |
| I604 | Subarachnoid haemorrhage from basilar artery | 152 |
| I605 | Subarachnoid haemorrhage from vertebral artery | 21 |
| I606 | Subarachnoid haemorrhage from other intracranial arteries | 138 |
| I607 | Subarachnoid haemorrhage from intracranial artery unspecified | 285 |
| I608 | Other subarachnoid haemorrhage | 411 |
| I609 | Subarachnoid haemorrhage unspecified | 3776 |
| I60X | NA | <5 |
| I61 | Intracerebral haemorrhage | 0 |
| I610 | Intracerebral haemorrhage in hemisphere subcortical | 409 |
| I611 | Intracerebral haemorrhage in hemisphere cortical | 683 |
| I612 | Intracerebral haemorrhage in hemisphere unspecified | 349 |
| I613 | Intracerebral haemorrhage in brain stem | 463 |
| I614 | Intracerebral haemorrhage in cerebellum | 616 |
| I615 | Intracerebral haemorrhage intraventricular | 953 |
| I616 | Intracerebral haemorrhage multiple localized | 130 |
| I618 | Other intracerebral haemorrhage | 1204 |
| I619 | Intracerebral haemorrhage unspecified | 8684 |
| I63 | Cerebral infarction | <5 |
| I630 | Cerebral infarction due to thrombosis of precerebral arteries | 468 |
| I631 | Cerebral infarction due to embolism of precerebral arteries | 78 |
| I632 | Cerebral infarction due to unspecified occlusion or stenosis of precerebral arteries | 907 |
| I633 | Cerebral infarction due to thrombosis of cerebral arteries | 2008 |
| I634 | Cerebral infarction due to embolism of cerebral arteries | 1062 |
| I635 | Cerebral infarction due to unspecified occlusion or stenosis of cerebral arteries | 2700 |
| I636 | Cerebral infarction due to cerebral venous thrombosis nonpyogenic | 35 |
| I638 | Other cerebral infarction | 8593 |
| I639 | Cerebral infarction unspecified | 42617 |
| I63X | NA | 16 |
| I64 | Stroke not specified as haemorrhage or infarction | 26727 |
| I64. | NA | 10 |
| I640 | NA | 45 |
| I649 | NA | <5 |
| I64D | NA | <5 |
| I64X | NA | 30322 |

## Descriptive statistics

140930 people had at least one diagnostic code in at least one of the datasets. 88093 people had a code in hospital admissions data, 44575 in mortality data and 89214 in primary care data. The following figure shows the year of the first code that was found for any person classified positive using (a) all codes combined, (b) only codes from hospital admissions data, (c) only codes from the mortality data and (d) only codes from primary care data.
