# Supplementary material for: The Secure Anonymised Information Linkage databank Dementia e-cohort (SAIL-DeC)
Source: Int J Popul Data Sci. 2020 Feb 25;5(1):1121. doi: 10.23889/ijpds.v5i1.1121 (PMC7473277; doi:10.23889/ijpds.v5i1.1121)
Supplement: Supplementary Material [file ijpds-05-01-1121-s001.zip › Supplementary Appendix 29.html]

Event tables


# Event tables

### *Substance misuse*

#### *Christian*

#### *January 2019*

## Code selection

We have selected codes based on Gorton, HC et al. Risk of unnatural mortality in people with epilepsy. JAMA Neurol. 2018 Apr 9 (list available from https://clinicalcodes.rss.mhs.man.ac.uk/medcodes/article/55/) in conjunction with the WHO ICD 10 browser (apps.who.int/classifications/icd10/browse/2010/en) and the NHS Read Code Browser (https://isd.digital.nhs.uk/trud3/user/guest/group/0/home). We have deliberately included codes with obvious `misspelling’ (for example having a dot where none should be) or ICD 10 codes ending with ‘X’.

Please be aware that we have excluded alcohol and tobacco misuse.

All codes that were selected for classification and the total number of people with at least one of the codes are displayed in the following tables. Please be aware that frequency counts of Read V2 codes in the table do not reflect the hierarchical nature of Read V2 coding (for example, counts of E01.. do not include E011.).

### Read V2 codes:

| code | desc | total\_n |
| --- | --- | --- |
| 13c.. | Drug user | 103 |
| 13c0. | Injecting drug user | 19 |
| 13c1. | Intravenous drug user | 37 |
| 13c2. | Never injecting drug user | 29 |
| 13c3. | Intramuscular drug user | <5 |
| 13c4. | Intranasal drug user | <5 |
| 13c5. | Substance misuse increased | 8 |
| 13c6. | Substance misuse decreased | <5 |
| 13c7. | Current drug user | 28 |
| 13c8. | Reduced drugs misuse | 5 |
| 13c9. | Subcutaneous drug user | 0 |
| 13cA. | Smokes drugs | 18 |
| 13cB. | Misuses drugs orally | 8 |
| 13cC. | Continuous use of drugs | <5 |
| 13cD. | Episodic use of drugs | <5 |
| 13cE. | Prolonged high dose use of cannabis | 11 |
| 13cF. | Preoccupied with substance misuse | 0 |
| 13cG. | Drug tolerance | 90 |
| 13cG0 | Opioid tolerant | 0 |
| 13cG1 | Opioid naive | <5 |
| 13cH. | Persistent substance misuse | 20 |
| 13cJ. | Previously injecting drug user | 5 |
| 13cK. | Current non recreational drug user | <5 |
| 13cL. | Has never injected drugs | <5 |
| 13cM. | Substance misuse | 138 |
| 13cM0 | Novel psychoactive substance misuse | 0 |
| 13cM1 | Opioid analgesic dependence | <5 |
| 13cN. | Has never shared drug injection equipment | 0 |
| 13cP. | Does not misuse drugs | 109 |
| 13cQ. | Behavioural tolerance to drug | 0 |
| 13cR. | Physical tolerance to drug | 0 |
| 13cS. | Psychological tolerance to drug | 0 |
| 13cT. | Reverse tolerance to drug | 0 |
| 146C. | Failed heroin detoxification | <5 |
| 146F. | H/O: drug abuse | 216 |
| 1T… | History of substance misuse | 78 |
| 1T0.. | H/O heroin misuse | 18 |
| 1T00. | H/O daily heroin misuse | <5 |
| 1T01. | H/O weekly heroin misuse | 0 |
| 1T02. | Previous history of heroin misuse | <5 |
| 1T03. | H/O infrequent heroin misuse | 0 |
| 1T1.. | H/O methadone misuse | <5 |
| 1T10. | H/O daily methadone misuse | 0 |
| 1T11. | H/O weekly methadone misuse | 0 |
| 1T12. | H/O infrequent methadone misuse | 0 |
| 1T13. | Previous history of methadone misuse | <5 |
| 1T2.. | H/O ecstasy misuse | 0 |
| 1T20. | H/O daily ecstasy misuse | 0 |
| 1T21. | H/O weekly ecstasy misuse | 0 |
| 1T22. | H/O infrequent ecstasy misuse | 0 |
| 1T23. | Previous history of ecstasy misuse | 0 |
| 1T3.. | H/O benzodiazepine misuse | 26 |
| 1T30. | H/O daily benzodiazepine misuse | 5 |
| 1T31. | H/O weekly benzodiazepine misuse | <5 |
| 1T32. | H/O infrequent benzodiazepine misuse | 5 |
| 1T33. | Previous history of benzodiazepine misuse | 6 |
| 1T4.. | H/O amphetamine misuse | 41 |
| 1T40. | H/O daily amphetamine misuse | <5 |
| 1T41. | H/O weekly amphetamine misuse | <5 |
| 1T42. | H/O infrequent amphetamine misuse | <5 |
| 1T43. | Previous history of amphetamine misuse | <5 |
| 1T5.. | H/O cocaine misuse | 13 |
| 1T50. | H/O daily cocaine misuse | 0 |
| 1T51. | H/O weekly cocaine misuse | 0 |
| 1T52. | H/O infrequent cocaine misuse | 0 |
| 1T53. | Previous history of cocaine misuse | <5 |
| 1T6.. | H/O crack cocaine misuse | 0 |
| 1T60. | H/O daily crack cocaine misuse | 0 |
| 1T61. | H/O weekly crack cocaine misuse | 0 |
| 1T62. | H/O infrequent crack cocaine misuse | 0 |
| 1T63. | Previous history of crack cocaine misuse | 0 |
| 1T7.. | H/O hallucinogen misuse | 0 |
| 1T70. | H/O daily hallucinogen misuse | 0 |
| 1T71. | H/O weekly hallucinogen misuse | 0 |
| 1T72. | H/O infrequent hallucinogen misuse | 0 |
| 1T73. | Previous history of hallucinogen misuse | 0 |
| 1T8.. | H/O cannabis misuse | 69 |
| 1T80. | H/O daily cannabis misuse | 23 |
| 1T81. | H/O weekly cannabis misuse | 9 |
| 1T82. | H/O infrequent cannabis misuse | 5 |
| 1T83. | Previous history of cannabis misuse | 11 |
| 1T9.. | H/O solvent misuse | 0 |
| 1T90. | H/O daily solvent misuse | 0 |
| 1T91. | H/O weekly solvent misuse | 0 |
| 1T92. | H/O infrequent solvent misuse | 0 |
| 1T93. | Previous history of solvent misuse | 0 |
| 1TA.. | H/O barbiturate misuse | <5 |
| 1TA0. | H/O daily barbiturate misuse | 0 |
| 1TA1. | H/O weekly barbiturate misuse | 0 |
| 1TA2. | H/O infrequent barbiturate misuse | 0 |
| 1TA3. | Previous history of barbiturate misuse | 0 |
| 1TB.. | H/O major tranquilliser misuse | <5 |
| 1TB0. | H/O daily major tranquilliser misuse | <5 |
| 1TB1. | H/O weekly major tranquilliser misuse | 0 |
| 1TB2. | H/O infrequent major tranquilliser misuse | 0 |
| 1TB3. | Previous history of major tranquilliser misuse | 0 |
| 1TC.. | H/O anti-depressant misuse | 0 |
| 1TC0. | H/O daily anti-depressant misuse | 6 |
| 1TC1. | H/O weekly anti-depressant misuse | 0 |
| 1TC2. | H/O infrequent anti-depressant misuse | 0 |
| 1TC3. | Previous history of anti-depressant misuse | 0 |
| 1TD.. | H/O opiate misuse | 33 |
| 1TD0. | H/O daily opiate misuse | 5 |
| 1TD1. | H/O weekly opiate misuse | 0 |
| 1TD2. | H/O infrequent opiate misuse | <5 |
| 1TD3. | Previous history of opiate misuse | <5 |
| 1TE.. | Uses heroin on top of substitution therapy | 0 |
| 1TF.. | Does not use heroin on top of substitution therapy | <5 |
| 1TG.. | H/O novel psychoactive substance misuse | 0 |
| 1V… | Drug misuse behaviour | 22 |
| 1V0.. | Misuses drugs | 9 |
| 1V00. | Occasional drug user | 0 |
| 1V01. | Long-term drug misuser | <5 |
| 1V02. | Poly-drug misuser | 0 |
| 1V03. | Misuses drugs sublingually | 0 |
| 1V04. | Misuses drugs rectally | 0 |
| 1V05. | Misuses drugs vaginally | 0 |
| 1V06. | Uses drug paraphernalia | 0 |
| 1V07. | Notified addict | 0 |
| 1V08. | Smokes drugs in cigarette form | <5 |
| 1V09. | Smokes drugs through a pipe | <5 |
| 1V0A. | Chases the dragon | 0 |
| 1V0B. | Sniffs drugs | 0 |
| 1V0C. | Drug addict | 0 |
| 1V0D. | Amount of money spent per day on drug habit | 0 |
| 1V0E. | Health problem secondary to drug misuse | 0 |
| 1V1.. | Time devoted to drug related activities | 0 |
| 1V10. | Time spent obtaining drugs | 0 |
| 1V11. | Time spent taking drugs | 0 |
| 1V12. | Time spent recovering from drugs | 0 |
| 1V2.. | Frequency of drug misuse | 0 |
| 1V22. | Age at starting drug misuse | 0 |
| 1V23. | Time since stopped drug misuse | 0 |
| 1V24. | Total time drugs misused | 0 |
| 1V25. | Has never misused drugs | 5 |
| 1V26. | Misused drugs in past | <5 |
| 1V3.. | Drug injection behaviour | <5 |
| 1V30. | Injects drugs subcutaneously | 7 |
| 1V31. | Injects drugs intramuscularly | <5 |
| 1V32. | Neck injector | 0 |
| 1V33. | Groin injector | 0 |
| 1V34. | Does not inject drugs | 0 |
| 1V35. | Shares drug equipment | 0 |
| 1V36. | Frontloading | 0 |
| 1V37. | Drug injecting equipment hygiene | 0 |
| 1V38. | Sharing of drug injecting equipment | 0 |
| 1V3A. | Does not share drug injection equipment | 0 |
| 1V3B. | Shares syringes | 0 |
| 1V3C. | Shares needles | 0 |
| 1V3D. | Cleaning of needles | 0 |
| 1V3E. | Cleans own needles | 0 |
| 1V3F. | Cleans needles with bleach | 0 |
| 1V3G. | Does not clean needles | 0 |
| 1V3H. | Obtains clean needles | 0 |
| 1V3J. | Uses needle exchange scheme | 0 |
| 1V3K. | Obtains clean syringes | 0 |
| 1V3L. | Use of needle and syringe exchange scheme | 0 |
| 1V3M. | Does not use needle and syringe exchange scheme | 0 |
| 1V3N. | Uses needle and syringe exchange scheme | 0 |
| 1V4.. | Priority of drug-related activities | 0 |
| 1V40. | No undue priority given to drug-related activities | 0 |
| 1V41. | Priority given to drug-related activities | 0 |
| 1V42. | Drug habit takes priority over social obligations | 0 |
| 1V43. | Drug habit takes priority over family obligations | 0 |
| 1V44. | Drug habit takes priority over financial obligations | 0 |
| 1V5.. | Routine of drug-related activities | <5 |
| 1V50. | Has no routine of drug-related activities | 0 |
| 1V51. | Has a routine of drug-related activities | 0 |
| 1V52. | Follows same drug-related routine every day | 0 |
| 1V53. | Drug-related rituals | 0 |
| 1V54. | Follows drug-related rituals | 0 |
| 1V55. | Does not follow drug-related rituals | 0 |
| 1V6.. | Drug-related offending behaviour | <5 |
| 1V60. | Dealing with drugs | 0 |
| 1V61. | Selling drugs | <5 |
| 1V62. | Buying drugs | <5 |
| 1V63. | Possession of drugs | <5 |
| 1V64. | Illicit drug use | 20 |
| 1V65. | Heroin misuse | 17 |
| 1V66. | Ecstasy misuse | 0 |
| 677T. | Substance misuse structured counselling | 0 |
| 7P220 | Delivery of rehabilitation for drug addiction | 9 |
| 8AA.. | Drug abuse monitoring | 31 |
| 8B23. | Drug addiction therapy | 613 |
| 8B230 | Drug addiction maintenance therapy - naltrexone | 0 |
| 8B231 | Drug addiction maintenance therapy - lofexidine | 0 |
| 8B2N. | Drug addiction detoxification therapy - methadone | 23 |
| 8B2P. | Drug addiction maintenance therapy - methadone | 19 |
| 8B2Q. | Drug addiction maintenance therapy - buprenorphine | 0 |
| 8B2R. | Drug addiction detoxification therapy - buprenorphine | 0 |
| 8B2S. | Opioid agonist substitution therapy | 0 |
| 8B2T. | Opioid antagonist therapy | 0 |
| 8BAd. | Opiate dependence detoxification | 38 |
| 8BAo. | Benzodiazepine dependence detoxification | 9 |
| 8BAW. | Drug dependence self detoxification | <5 |
| 8BAX. | Drug dependence home detoxification | 25 |
| 8H7x. | Referral to drug abuse counsellor | 163 |
| 8Hh1. | Self referral to substance misuse service | 0 |
| 8HkF. | Referral to substance misuse service | 85 |
| 8Hq.. | Admission to substance misuse detoxification centre | 12 |
| 9G2.. | Drug addiction notification | 27 |
| 9G21. | Drug addict notific to CMO | <5 |
| 9G22. | Drug addict re-notific due | 0 |
| 9G23. | Drug addict re-notif to CMO | <5 |
| 9G2Z. | Drug addiction notif NOS | <5 |
| 9HC.. | Substance misuse monitoring | 162 |
| 9HC0. | Initial substance misuse assessment | 10 |
| 9HC1. | Follow up substance misuse assessment | 12 |
| 9HC2. | Substance misuse clinical management plan agreed | 20 |
| 9HC3. | Substance misuse clinical management plan reviewed | 7 |
| 9HC4. | Substance misuse treatment withdrawn | <5 |
| 9HC5. | Substance misuse treatment programme completed | <5 |
| 9HC6. | Substance misuse treatment declined | 0 |
| 9HC7. | Substance misuse treatment not available | 0 |
| 9HC8. | Declined to give substance misuse history | 0 |
| 9HC9. | Substance misuse treatment programme delivered by other healthcare provider | 0 |
| 9HCA. | Substance misuse monitoring 6 month review | <5 |
| 9HCB. | Substance misuse monitoring annual review | 0 |
| 9HCC. | On substance misuse programme | <5 |
| 9k5.. | Drug misuse - enhanced services administration | 21 |
| 9k50. | Drug misuse - enhanced service completed | 13 |
| 9k51. | Shared care drug misuse treatment - enhanced services administration | 9 |
| 9k52. | Drug misuse treatment in primary care - enhanced services administration | <5 |
| 9k53. | Pharmacy attended for drug misuse - enhanced services administration | 0 |
| 9kS.. | Drug misuse assessment declined - enhanced services administration | 0 |
| 9N1yJ | Seen in drug misuse clinic | 22 |
| 9N4i. | DNA - Did not attend substance misuse clinic | 46 |
| 9NdN. | Declined consent for notification of drug misuse | 0 |
| 9No5. | Seen in substance misuse clinic | 208 |
| 9s… | Drug misuse clinic administration | <5 |
| E24.. | Drug dependence | 1902 |
| E240. | Opioid type drug dependence | 384 |
| E2400 | Unspecified opioid dependence | 11 |
| E2401 | Continuous opioid dependence | 16 |
| E2402 | Episodic opioid dependence | 6 |
| E2403 | Opioid dependence in remission | 5 |
| E240z | Opioid drug dependence NOS | 83 |
| E241. | Hypnotic or anxiolytic dependence | 6064 |
| E2410 | Hypnotic or anxiolytic dependence, unspecified | 58 |
| E2411 | Hypnotic or anxiolytic dependence, continuous | 171 |
| E2412 | Hypnotic or anxiolytic dependence, episodic | 37 |
| E2413 | Hypnotic or anxiolytic dependence in remission | 9 |
| E241z | Hypnotic or anxiolytic dependence NOS | 169 |
| E242. | Cocaine type drug dependence | 31 |
| E2420 | Cocaine dependence, unspecified | 6 |
| E2421 | Cocaine dependence, continuous | <5 |
| E2422 | Cocaine dependence, episodic | <5 |
| E2423 | Cocaine dependence in remission | <5 |
| E242z | Cocaine drug dependence NOS | <5 |
| E243. | Cannabis type drug dependence | 73 |
| E2430 | Cannabis dependence, unspecified | 36 |
| E2431 | Cannabis dependence, continuous | 16 |
| E2432 | Cannabis dependence, episodic | <5 |
| E2433 | Cannabis dependence in remission | <5 |
| E243z | Cannabis drug dependence NOS | <5 |
| E244. | Amphetamine or other psychostimulant dependence | 83 |
| E2440 | Amphetamine or psychostimulant dependence, unspecified | 11 |
| E2441 | Amphetamine or psychostimulant dependence, continuous | 9 |
| E2442 | Amphetamine or psychostimulant dependence, episodic | <5 |
| E2443 | Amphetamine or psychostimulant dependence in remission | <5 |
| E244z | Amphetamine or psychostimulant dependence NOS | 23 |
| E245. | Hallucinogen dependence | 16 |
| E2450 | Hallucinogen dependence, unspecified | <5 |
| E2451 | Hallucinogen dependence, continuous | 0 |
| E2452 | Hallucinogen dependence, episodic | 0 |
| E2453 | Hallucinogen dependence in remission | 0 |
| E245z | Hallucinogen dependence NOS | 0 |
| E246. | Glue sniffing dependence | 5 |
| E2460 | Glue sniffing dependence, unspecified | 0 |
| E2461 | Glue sniffing dependence, continuous | 0 |
| E2462 | Glue sniffing dependence, episodic | 0 |
| E2463 | Glue sniffing dependence in remission | 0 |
| E246z | Glue sniffing dependence NOS | 0 |
| E247. | Other specified drug dependence | 23 |
| E2470 | Other specified drug dependence, unspecified | <5 |
| E2471 | Other specified drug dependence, continuous | 5 |
| E2472 | Other specified drug dependence, episodic | <5 |
| E2473 | Other specified drug dependence in remission | <5 |
| E247z | Other specified drug dependence NOS | <5 |
| E248. | Combined opioid with other drug dependence | 19 |
| E2480 | Combined opioid with other drug dependence, unspecified | 0 |
| E2481 | Combined opioid with other drug dependence, continuous | <5 |
| E2482 | Combined opioid with other drug dependence, episodic | <5 |
| E2483 | Combined opioid with other drug dependence in remission | <5 |
| E248z | Combined opioid with other drug dependence NOS | <5 |
| E249. | Combined drug dependence, excluding opioids | 12 |
| E2490 | Combined drug dependence, excluding opioid, unspecified | 0 |
| E2491 | Combined drug dependence, excluding opioid, continuous | 0 |
| E2492 | Combined drug dependence, excluding opioid, episodic | 0 |
| E2493 | Combined drug dependence, excluding opioid, in remission | 0 |
| E249z | Combined drug dependence, excluding opioid, NOS | 0 |
| E24A. | Ecstasy type drug dependence | <5 |
| E24z. | Drug dependence NOS | 176 |
| E252. | Nondependent cannabis abuse | 138 |
| E2520 | Nondependent cannabis abuse, unspecified | 11 |
| E2521 | Nondependent cannabis abuse, continuous | <5 |
| E2522 | Nondependent cannabis abuse, episodic | 7 |
| E2523 | Nondependent cannabis abuse in remission | 0 |
| E252z | Nondependent cannabis abuse NOS | 9 |
| E253. | Nondependent hallucinogen abuse | 24 |
| E2530 | Nondependent hallucinogen abuse, unspecified | <5 |
| E2531 | Nondependent hallucinogen abuse, continuous | 0 |
| E2532 | Nondependent hallucinogen abuse, episodic | 0 |
| E2533 | Nondependent hallucinogen abuse in remission | 0 |
| E253z | Nondependent hallucinogen abuse NOS | 0 |
| E254. | Nondependent hypnotic or anxiolytic abuse | 69 |
| E2540 | Nondependent hypnotic or anxiolytic abuse, unspecified | 23 |
| E2541 | Nondependent hypnotic or anxiolytic abuse, continuous | <5 |
| E2542 | Nondependent hypnotic or anxiolytic abuse, episodic | 0 |
| E2543 | Nondependent hypnotic or anxiolytic abuse in remission | 0 |
| E254z | Nondependent hypnotic or anxiolytic abuse NOS | <5 |
| E255. | Nondependent opioid abuse | 5 |
| E2550 | Nondependent opioid abuse, unspecified | <5 |
| E2551 | Nondependent opioid abuse, continuous | <5 |
| E2552 | Nondependent opioid abuse, episodic | <5 |
| E2553 | Nondependent opioid abuse in remission | 0 |
| E255z | Nondependent opioid abuse NOS | 0 |
| E256. | Nondependent cocaine abuse | 7 |
| E2560 | Nondependent cocaine abuse, unspecified | <5 |
| E2561 | Nondependent cocaine abuse, continuous | 0 |
| E2562 | Nondependent cocaine abuse, episodic | <5 |
| E2563 | Nondependent cocaine abuse in remission | <5 |
| E256z | Nondependent cocaine abuse NOS | 0 |
| E257. | Nondependent amphetamine or other psychostimulant abuse | 39 |
| E2570 | Nondependent amphetamine or psychostimulant abuse, unspecified | <5 |
| E2571 | Nondependent amphetamine or psychostimulant abuse, continuous | <5 |
| E2572 | Nondependent amphetamine or psychostimulant abuse, episodic | <5 |
| E2573 | Nondependent amphetamine or psychostimulant abuse in remission | <5 |
| E257z | Nondependent amphetamine or psychostimulant abuse NOS | <5 |
| E258. | Nondependent antidepressant type drug abuse | 7 |
| E2580 | Nondependent antidepressant type drug abuse, unspecified | 0 |
| E2581 | Nondependent antidepressant type drug abuse, continuous | <5 |
| E2582 | Nondependent antidepressant type drug abuse, episodic | 0 |
| E2583 | Nondependent antidepressant type drug abuse in remission | 0 |
| E258z | Nondependent antidepressant type drug abuse NOS | 0 |
| E259. | Nondependent mixed drug abuse | 8 |
| E2590 | Nondependent mixed drug abuse, unspecified | 8 |
| E2591 | Nondependent mixed drug abuse, continuous | <5 |
| E2592 | Nondependent mixed drug abuse, episodic | <5 |
| E2593 | Nondependent mixed drug abuse in remission | <5 |
| E2594 | Misuse of prescription only drugs | 81 |
| E259z | Nondependent mixed drug abuse NOS | <5 |
| E25y. | Nondependent other drug abuse | 187 |
| E25y0 | Nondependent other drug abuse, unspecified | <5 |
| E25y1 | Nondependent other drug abuse, continuous | 0 |
| E25y2 | Nondependent other drug abuse, episodic | <5 |
| E25y3 | Nondependent other drug abuse in remission | 0 |
| E25yz | Nondependent other drug abuse NOS | 9 |
| E25z. | Misuse of drugs NOS | 495 |
| Eu11. | [X]Mental and behavioural disorders due to use of opioids | 6 |
| Eu110 | [X]Mental and behavioural disorders due to use of opioids: acute intoxication | <5 |
| Eu111 | [X]Mental and behavioural disorders due to use of opioids: harmful use | <5 |
| Eu112 | [X]Mental and behavioural disorders due to use of opioids: dependence syndrome | 167 |
| Eu113 | [X]Mental and behavioural disorders due to use of opioids: withdrawal state | 58 |
| Eu114 | [X]Mental and behavioural disorders due to use of opioids: withdrawal state with delirium | 0 |
| Eu115 | [X]Mental and behavioural disorders due to use of opioids: psychotic disorder | 0 |
| Eu116 | [X]Mental and behavioural disorders due to use of opioids: amnesic syndrome | 0 |
| Eu117 | [X]Mental and behavioural disorders due to use of opioids: residual and late-onset psychotic disorder | 0 |
| Eu11y | [X]Mental and behavioural disorders due to use of opioids: other mental and behavioural disorders | 0 |
| Eu11z | [X]Mental and behavioural disorders due to use of opioids: unspecified mental and behavioural disorder | <5 |
| Eu12. | [X]Mental and behavioural disorders due to use of cannabinoids | <5 |
| Eu120 | [X]Mental and behavioural disorders due to use of cannabinoids: acute intoxication | <5 |
| Eu121 | [X]Mental and behavioural disorders due to use of cannabinoids: harmful use | <5 |
| Eu122 | [X]Mental and behavioural disorders due to use of cannabinoids: dependence syndrome | 51 |
| Eu123 | [X]Mental and behavioural disorders due to use of cannabinoids: withdrawal state | <5 |
| Eu124 | [X]Mental and behavioural disorders due to use of cannabinoids: withdrawal state with delirium | 0 |
| Eu125 | [X]Mental and behavioural disorders due to use of cannabinoids: psychotic disorder | <5 |
| Eu126 | [X]Mental and behavioural disorders due to use of cannabinoids: amnesic syndrome | 0 |
| Eu127 | [X]Mental and behavioural disorders due to use of cannabinoids: residual and late-onset psychotic disorder | 0 |
| Eu12y | [X]Mental and behavioural disorders due to use of cannabinoids: other mental and behavioural disorders | 0 |
| Eu12z | [X]Mental and behavioural disorders due to use of cannabinoids: unspecified mental and behavioural disorder | 0 |
| Eu13. | [X]Mental and behavioural disorders due to use of sedatives or hypnotics | <5 |
| Eu130 | [X]Mental and behavioural disorders due to use of sedatives or hypnotics: acute intoxication | 0 |
| Eu131 | [X]Mental and behavioural disorders due to use of sedatives or hypnotics: harmful use | <5 |
| Eu132 | [X]Mental and behavioural disorders due to use of sedatives or hypnotics: dependence syndrome | 52 |
| Eu133 | [X]Mental and behavioural disorders due to use of sedatives or hypnotics: withdrawal state | <5 |
| Eu134 | [X]Mental and behavioural disorders due to use of sedatives or hypnotics: withdrawal state with delirium | 0 |
| Eu135 | [X]Mental and behavioural disorders due to use of sedatives or hypnotics: psychotic disorder | 0 |
| Eu136 | [X]Mental and behavioural disorders due to use of sedatives or hypnotics: amnesic syndrome | 0 |
| Eu137 | [X]Mental and behavioural disorders due to use of sedatives or hypnotics: residual and late-onset psychotic disorder | <5 |
| Eu13y | [X]Mental and behavioural disorders due to use of sedatives or hypnotics: other mental and behavioural disorders | 0 |
| Eu13z | [X]Mental and behavioural disorders due to use of sedatives or hypnotics: unspecified mental and behavioural disorder | 0 |
| Eu14. | [X]Mental and behavioural disorders due to use of cocaine | 0 |
| Eu140 | [X]Mental and behavioural disorders due to use of cocaine: acute intoxication | 0 |
| Eu141 | [X]Mental and behavioural disorders due to use of cocaine: harmful use | 0 |
| Eu142 | [X]Mental and behavioural disorders due to use of cocaine: dependence syndrome | 7 |
| Eu143 | [X]Mental and behavioural disorders due to use of cocaine: withdrawal state | 0 |
| Eu144 | [X]Mental and behavioural disorders due to use of cocaine: withdrawal state with delirium | 0 |
| Eu145 | [X]Mental and behavioural disorders due to use of cocaine: psychotic disorder | 0 |
| Eu146 | [X]Mental and behavioural disorders due to use of cocaine: amnesic syndrome | 0 |
| Eu147 | [X]Mental and behavioural disorders due to use of cocaine: residual and late-onset psychotic disorder | 0 |
| Eu14y | [X]Mental and behavioural disorders due to use of cocaine: other mental and behavioural disorders | 0 |
| Eu14z | [X]Mental and behavioural disorders due to use of cocaine: unspecified mental and behavioural disorder | 0 |
| Eu15. | [X]Mental and behavioural disorders due to use of other stimulants, including caffeine | 0 |
| Eu150 | [X]Mental and behavioural disorders due to use of other stimulants, including caffeine: acute intoxication | <5 |
| Eu151 | [X]Mental and behavioural disorders due to use of other stimulants, including caffeine: harmful use | 0 |
| Eu152 | [X]Mental and behavioural disorders due to use of other stimulants, including caffeine: dependence syndrome | 29 |
| Eu153 | [X]Mental and behavioural disorders due to use of other stimulants, including caffeine: withdrawal state | <5 |
| Eu154 | [X]Mental and behavioural disorders due to use of other stimulants, including caffeine: withdrawal state with delirium | 0 |
| Eu155 | [X]Mental and behavioural disorders due to use of other stimulants, including caffeine: psychotic disorder | 0 |
| Eu156 | [X]Mental and behavioural disorders due to use of other stimulants, including caffeine: amnesic syndrome | 0 |
| Eu157 | [X]Mental and behavioural disorders due to use of other stimulants, including caffeine: residual and late-onset psychotic disorder | <5 |
| Eu15y | [X]Mental and behavioural disorders due to use of other stimulants, including caffeine: other mental and behavioural disorders | 0 |
| Eu15z | [X]Mental and behavioural disorders due to use of other stimulants, including caffeine: unspecified mental and behavioural disorder | 0 |
| Eu16. | [X]Mental and behavioural disorders due to use of hallucinogens | 0 |
| Eu160 | [X]Mental and behavioural disorders due to use of hallucinogens: acute intoxication | 0 |
| Eu161 | [X]Mental and behavioural disorders due to use of hallucinogens: harmful use | 0 |
| Eu162 | [X]Mental and behavioural disorders due to use of hallucinogens: dependence syndrome | <5 |
| Eu163 | [X]Mental and behavioural disorders due to use of hallucinogens: withdrawal state | 0 |
| Eu164 | [X]Mental and behavioural disorders due to use of hallucinogens: withdrawal state with delirium | <5 |
| Eu165 | [X]Mental and behavioural disorders due to use of hallucinogens: psychotic disorder | <5 |
| Eu166 | [X]Mental and behavioural disorders due to use of hallucinogens: amnesic syndrome | 0 |
| Eu167 | [X]Mental and behavioural disorders due to use of hallucinogens: residual and late-onset psychotic disorder | 0 |
| Eu16y | [X]Mental and behavioural disorders due to use of hallucinogens: other mental and behavioural disorders | 0 |
| Eu16z | [X]Mental and behavioural disorders due to use of hallucinogens: unspecified mental and behavioural disorder | 0 |
| Eu18. | [X]Mental and behavioural disorders due to use of volatile solvents | 0 |
| Eu180 | [X]Mental and behavioural disorders due to use of volatile solvents: acute intoxication | <5 |
| Eu181 | [X]Mental and behavioural disorders due to use of volatile solvents: harmful use | 0 |
| Eu182 | [X]Mental and behavioural disorders due to use of volatile solvents: dependence syndrome | <5 |
| Eu183 | [X]Mental and behavioural disorders due to use of volatile solvents: withdrawal state | 0 |
| Eu184 | [X]Mental and behavioural disorders due to use of volatile solvents: withdrawal state with delirium | 0 |
| Eu185 | [X]Mental and behavioural disorders due to use of volatile solvents: psychotic disorder | 0 |
| Eu186 | [X]Mental and behavioural disorders due to use of volatile solvents: amnesic syndrome | 0 |
| Eu187 | [X]Mental and behavioural disorders due to use of volatile solvents: residual and late-onset psychotic disorder | 0 |
| Eu18y | [X]Mental and behavioural disorders due to use of volatile solvents: other mental and behavioural disorders | 0 |
| Eu18z | [X]Mental and behavioural disorders due to use of volatile solvents: unspecified mental and behavioural disorder | 0 |
| Eu19. | [X]Mental and behavioural disorders due to multiple drug use and use of other psychoactive substances | <5 |
| Eu190 | [X]Mental and behavioural disorders due to multiple drug use and use of other psychoactive substances: acute intoxication | 0 |
| Eu191 | [X]Mental and behavioural disorders due to multiple drug use and use of other psychoactive substances: harmful use | 0 |
| Eu192 | [X]Mental and behavioural disorders due to multiple drug use and use of other psychoactive substances: dependence syndrome | 33 |
| Eu193 | [X]Mental and behavioural disorders due to multiple drug use and use of other psychoactive substances: withdrawal state | <5 |
| Eu194 | [X]Mental and behavioural disorders due to multiple drug use and use of other psychoactive substances: withdrawal state with delirium | 0 |
| Eu195 | [X]Mental and behavioural disorders due to multiple drug use and use of other psychoactive substances: psychotic disorder | <5 |
| Eu196 | [X]Mental and behavioural disorders due to multiple drug use and use of other psychoactive substances: amnesic syndrome | 0 |
| Eu197 | [X]Mental and behavioural disorders due to multiple drug use and use of other psychoactive substances: residual and late-onset psychotic | 0 |
| Eu19y | [X]Mental and behavioural disorders due to multiple drug use and use of other psychoactive substances: other mental and behavioural disorders | 0 |
| Eu19z | [X]Mental and behavioural disorders due to multiple drug use and use of other psychoactive substances: unspecified mental and behavioural disorder | 0 |
| Eu1A. | [X]Mental and behavioural disorders due to use of crack cocaine | 0 |
| Eu1A0 | [X]Mental and behavioural disorders due to use of crack cocaine: acute intoxication | 0 |
| Eu1A1 | [X]Mental and behavioural disorders due to use of crack cocaine: harmful use | 0 |
| Eu1A2 | [X]Mental and behavioural disorders due to use of crack cocaine: dependence syndrome | <5 |
| Eu1A3 | [X]Mental and behavioural disorders due to use of crack cocaine: withdrawal state | 0 |
| Eu1A4 | [X]Mental and behavioural disorders due to use of crack cocaine: withdrawal state with delirium | 0 |
| Eu1A5 | [X]Mental and behavioural disorders due to use of crack cocaine: psychotic disorder | 0 |
| Eu1A6 | [X]Mental and behavioural disorders due to use of crack cocaine: amnesic syndrome | 0 |
| Eu1A7 | [X]Mental and behavioural disorders due to use of crack cocaine: residual and late-onset psychotic disorder | 0 |
| Eu1Ay | [X]Mental and behavioural disorders due to use of crack cocaine: other mental and behavioural disorders | 0 |
| Eu1Az | [X]Mental and behavioural disorders due to use of crack cocaine: unspecified mental and behavioural disorder | 0 |
| Eu55. | [X]Abuse of non-dependence-producing substances | 71 |
| L183. | Drug dependence during pregnancy, childbirth and the puerperium | <5 |
| L1830 | Drug dependence - unspecified whether during pregnancy or the puerperium | 0 |
| L1831 | Drug dependence during pregnancy - baby delivered | 0 |
| L1832 | Drug dependence in the puerperium - baby delivered | 0 |
| L1833 | Drug dependence during pregnancy - baby not yet delivered | 0 |
| L1834 | Drug dependence in the puerperium - baby delivered during previous episode of care | 0 |
| L183z | Drug dependence during pregnancy, childbirth or the puerperium NOS | 0 |
| ZV114 | [V]Personal history of psychoactive substance abuse | 17 |
| ZV115 | [V]Personal history of drug abuse by injection | 12 |

### ICD 9 and 10 codes:

| code | desc | total\_n |
| --- | --- | --- |
| 2920 | Drug withdrawal syndrome | <5 |
| 2929 | Unspecified | 0 |
| 2940 | Korsakov s psychosis or syndrome (nonalcoholic) | <5 |
| 304 | Drug dependence | 0 |
| 3040 | Morphine type | <5 |
| 3041 | Barbiturate type | <5 |
| 3042 | Cocaine | <5 |
| 3043 | Cannabis | 0 |
| 3044 | Amphetamine type and other psychostimulants | <5 |
| 3045 | Hallucinogens | 0 |
| 3046 | Other | 0 |
| 3047 | Combinations of morphine type drug with any other | <5 |
| 3048 | Combinations excluding morphine type drug | 0 |
| 3049 | Unspecified | <5 |
| F11 | Mental and behavioural disorders due to use of opioids | 9 |
| F11. | NA | <5 |
| F110 | Mental and behavioural disorders due to use of opioids | 66 |
| F111 | Mental and behavioural disorders due to use of opioids | 189 |
| F112 | Mental and behavioural disorders due to use of opioids | 798 |
| F113 | Mental and behavioural disorders due to use of opioids | 100 |
| F114 | Mental and behavioural disorders due to use of opioids | 14 |
| F115 | Mental and behavioural disorders due to use of opioids | 24 |
| F116 | Mental and behavioural disorders due to use of opioids | 10 |
| F117 | Mental and behavioural disorders due to use of opioids | <5 |
| F118 | Mental and behavioural disorders due to use of opioids | <5 |
| F119 | Mental and behavioural disorders due to use of opioids | 28 |
| F11X | NA | 13 |
| F12 | Mental and behavioural disorders due to use of cannabinoids | <5 |
| F120 | Mental and behavioural disorders due to use of cannabinoids | 36 |
| F121 | Mental and behavioural disorders due to use of cannabinoids | 415 |
| F122 | Mental and behavioural disorders due to use of cannabinoids | 75 |
| F123 | Mental and behavioural disorders due to use of cannabinoids | 6 |
| F124 | Mental and behavioural disorders due to use of cannabinoids | <5 |
| F125 | Mental and behavioural disorders due to use of cannabinoids | 30 |
| F126 | Mental and behavioural disorders due to use of cannabinoids | <5 |
| F127 | Mental and behavioural disorders due to use of cannabinoids | <5 |
| F128 | Mental and behavioural disorders due to use of cannabinoids | <5 |
| F129 | Mental and behavioural disorders due to use of cannabinoids | 18 |
| F12X | NA | 12 |
| F13 | Mental and behavioural disorders due to use of sedatives or hypnotics | 5 |
| F13. | NA | <5 |
| F130 | Mental and behavioural disorders due to use of sedatives or hypnotics | 16 |
| F131 | Mental and behavioural disorders due to use of sedatives or hypnotics | 58 |
| F132 | Mental and behavioural disorders due to use of sedatives or hypnotics | 291 |
| F133 | Mental and behavioural disorders due to use of sedatives or hypnotics | 81 |
| F134 | Mental and behavioural disorders due to use of sedatives or hypnotics | 14 |
| F135 | Mental and behavioural disorders due to use of sedatives or hypnotics | 13 |
| F136 | Mental and behavioural disorders due to use of sedatives or hypnotics | <5 |
| F137 | Mental and behavioural disorders due to use of sedatives or hypnotics | 14 |
| F138 | Mental and behavioural disorders due to use of sedatives or hypnotics | <5 |
| F139 | Mental and behavioural disorders due to use of sedatives or hypnotics | 13 |
| F13X | NA | 19 |
| F14 | Mental and behavioural disorders due to use of cocaine | 0 |
| F14. | NA | <5 |
| F140 | Mental and behavioural disorders due to use of cocaine | 10 |
| F141 | Mental and behavioural disorders due to use of cocaine | 60 |
| F142 | Mental and behavioural disorders due to use of cocaine | 30 |
| F143 | Mental and behavioural disorders due to use of cocaine | <5 |
| F144 | Mental and behavioural disorders due to use of cocaine | <5 |
| F145 | Mental and behavioural disorders due to use of cocaine | 9 |
| F146 | Mental and behavioural disorders due to use of cocaine | 0 |
| F147 | Mental and behavioural disorders due to use of cocaine | <5 |
| F148 | Mental and behavioural disorders due to use of cocaine | 0 |
| F149 | Mental and behavioural disorders due to use of cocaine | 13 |
| F14X | NA | <5 |
| F16 | Mental and behavioural disorders due to use of hallucinogens | 0 |
| F160 | Mental and behavioural disorders due to use of hallucinogens | 8 |
| F161 | Mental and behavioural disorders due to use of hallucinogens | 20 |
| F162 | Mental and behavioural disorders due to use of hallucinogens | 27 |
| F163 | Mental and behavioural disorders due to use of hallucinogens | 7 |
| F164 | Mental and behavioural disorders due to use of hallucinogens | 0 |
| F165 | Mental and behavioural disorders due to use of hallucinogens | 14 |
| F166 | Mental and behavioural disorders due to use of hallucinogens | <5 |
| F167 | Mental and behavioural disorders due to use of hallucinogens | 7 |
| F168 | Mental and behavioural disorders due to use of hallucinogens | 0 |
| F169 | Mental and behavioural disorders due to use of hallucinogens | <5 |
| F16X | NA | <5 |
| F18 | Mental and behavioural disorders due to use of volatile solvents | 0 |
| F180 | Mental and behavioural disorders due to use of volatile solvents | <5 |
| F181 | Mental and behavioural disorders due to use of volatile solvents | 19 |
| F182 | Mental and behavioural disorders due to use of volatile solvents | 9 |
| F183 | Mental and behavioural disorders due to use of volatile solvents | <5 |
| F184 | Mental and behavioural disorders due to use of volatile solvents | <5 |
| F185 | Mental and behavioural disorders due to use of volatile solvents | 0 |
| F186 | Mental and behavioural disorders due to use of volatile solvents | 0 |
| F187 | Mental and behavioural disorders due to use of volatile solvents | 0 |
| F188 | Mental and behavioural disorders due to use of volatile solvents | <5 |
| F189 | Mental and behavioural disorders due to use of volatile solvents | 7 |
| F19 | Mental and behavioural disorders due to multiple drug use and use of other psychoactive substances | 6 |
| F190 | Mental and behavioural disorders due to multiple drug use and use of other psychoactive substances | 33 |
| F191 | Mental and behavioural disorders due to multiple drug use and use of other psychoactive substances | 195 |
| F192 | Mental and behavioural disorders due to multiple drug use and use of other psychoactive substances | 223 |
| F193 | Mental and behavioural disorders due to multiple drug use and use of other psychoactive substances | 44 |
| F194 | Mental and behavioural disorders due to multiple drug use and use of other psychoactive substances | 8 |
| F195 | Mental and behavioural disorders due to multiple drug use and use of other psychoactive substances | 65 |
| F196 | Mental and behavioural disorders due to multiple drug use and use of other psychoactive substances | <5 |
| F197 | Mental and behavioural disorders due to multiple drug use and use of other psychoactive substances | 5 |
| F198 | Mental and behavioural disorders due to multiple drug use and use of other psychoactive substances | 6 |
| F199 | Mental and behavioural disorders due to multiple drug use and use of other psychoactive substances | 38 |
| F19U | NA | <5 |
| F19X | NA | 35 |
| T40 | Poisoning by narcotics and psychodysleptics [hallucinogens] | 0 |
| T400 | Poisoning: Opium | 13 |
| T401 | Poisoning: Heroin | 163 |
| T402 | Poisoning: Other opioids | 2615 |
| T403 | Poisoning: Methadone | 71 |
| T404 | Poisoning: Other synthetic narcotics | 570 |
| T405 | Poisoning: Cocaine | 40 |
| T406 | Poisoning: Other and unspecified narcotics | 347 |
| T407 | Poisoning: Cannabis (derivatives) | 72 |
| T408 | Poisoning: Lysergide [LSD] | 11 |
| T409 | Poisoning: Other and unspecified psychodysleptics [hallucinogens] | 10 |
| X42 | Accidental poisoning by and exposure to narcotics and psychodysleptics drugs | <5 |
| X420 | Accidental poisoning by and exposure to narcotics and psychodysleptics drugs | 770 |
| X421 | Accidental poisoning by and exposure to narcotics and psychodysleptics drugs | 51 |
| X422 | Accidental poisoning by and exposure to narcotics and psychodysleptics drugs | 134 |
| X424 | Accidental poisoning by and exposure to narcotics and psychodysleptics drugs | 9 |
| X425 | Accidental poisoning by and exposure to narcotics and psychodysleptics drugs | <5 |
| X428 | Accidental poisoning by and exposure to narcotics and psychodysleptics drugs | 18 |
| X429 | Accidental poisoning by and exposure to narcotics and psychodysleptics drugs | 559 |
| X62 | Intentional poisoning by and exposure to narcotics and psychodysleptics drugs | <5 |
| X620 | Intentional poisoning by and exposure to narcotics and psychodysleptics drugs | 1009 |
| X621 | Intentional poisoning by and exposure to narcotics and psychodysleptics drugs | 6 |
| X622 | Intentional poisoning by and exposure to narcotics and psychodysleptics drugs | 10 |
| X624 | Intentional poisoning by and exposure to narcotics and psychodysleptics drugs | 10 |
| X625 | Intentional poisoning by and exposure to narcotics and psychodysleptics drugs | 13 |
| X627 | Intentional poisoning by and exposure to narcotics and psychodysleptics drugs | <5 |
| X628 | Intentional poisoning by and exposure to narcotics and psychodysleptics drugs | 35 |
| X629 | Intentional poisoning by and exposure to narcotics and psychodysleptics drugs | 906 |
| X62X | Intentional poisoning by and exposure to narcotics and psychodysleptics drugs | <5 |

## Descriptives

16660 people had at least one diagnostic code in at least one of the datasets. 6094 people had a code in hospital admissions data, 271 in mortality data and 11262 in primary care data. The following figure shows the year of the first code that was found for any person classified positive using (a) all codes combined, (b) only codes from hospital admissions data, (c) only codes from the mortality data and (d) only codes from primary care data.
