# Supplementary material for: The Secure Anonymised Information Linkage databank Dementia e-cohort (SAIL-DeC)
Source: Int J Popul Data Sci. 2020 Feb 25;5(1):1121. doi: 10.23889/ijpds.v5i1.1121 (PMC7473277; doi:10.23889/ijpds.v5i1.1121)
Supplement: Supplementary Material [file ijpds-05-01-1121-s001.zip › Supplementary Appendix 7.docx]

**Supplementary Appendix 4. Dementia case identification across datasets**

Figure S1 demonstrates the datasets in which we identified dementia cases at any point in time. Figure S2 shows the order in which we identified dementia cases across datasets. Of the 129,650 participants who developed dementia, 62,129 (48%) first received a dementia code in primary care data. Of these, 39,179 (63%) subsequently received a dementia code in hospital admissions data and 25,357 (41%) received a later dementia code in mortality data. 20,290 participants received a dementia code in both hospital admissions and mortality data following their first dementia code in primary care. The median time from first primary care dementia code to first dementia code in hospital admissions data was 520 days. The median time from first primary care dementia code to receiving a dementia code in death data was 1440 days for participants who also received a dementia code in hospital admissions data, and 988 days for those without a hospital record of dementia.

62,413 (48%) participants first received a dementia code in hospital admissions data. Of these, 16,588 (27%) subsequently received a dementia code in primary care data and 21,778 (35%) received a dementia code in mortality data. 8,515 (14%) participants received a dementia code in both primary care and mortality data after first appearing in hospital admissions data. The median time from first hospital admissions dementia code to receiving a primary care dementia code was 160 days. The median times from first dementia code in hospital admissions data to a dementia code in mortality data for participants with and without a primary care dementia code were 237 days and 1227 days respectively.

5,069 (4%) participants only received a dementia code in mortality data.


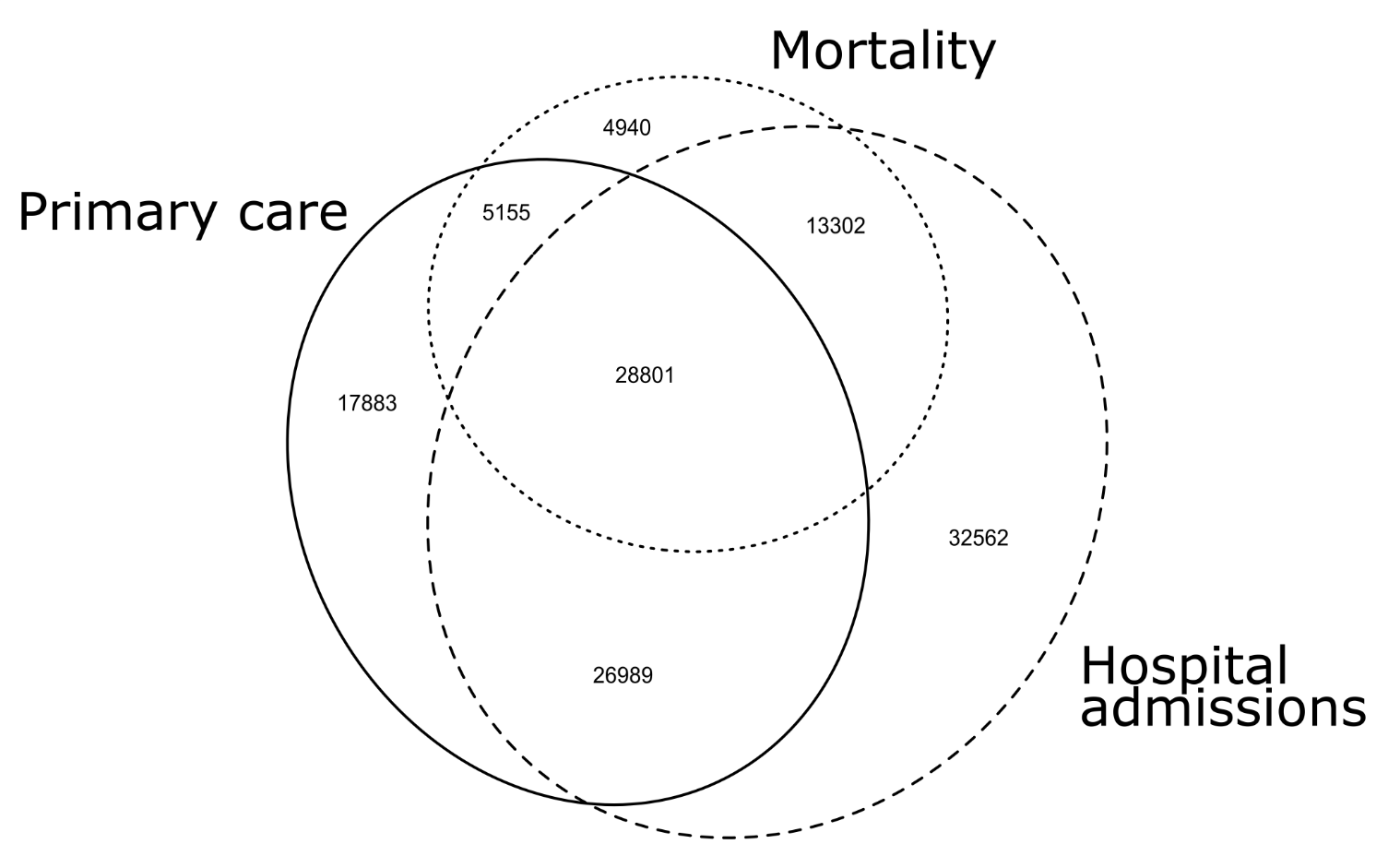


**Figure S1. Area proportional Euler diagram demonstrating the datasets in which dementia cases were identified at any time during follow-up (n=129,650)**

**
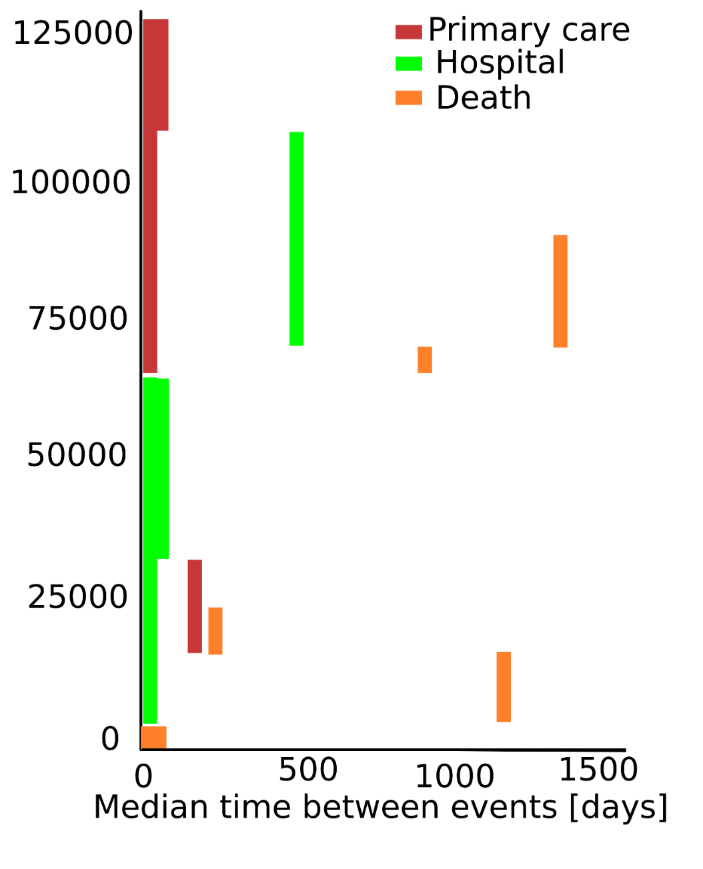
**

**Figure S2. Event flow diagram, demonstrating the order in which dementia cases were identified across datasets**

Participants are grouped based on the order in which they were identified across primary care, hospital admissions and mortality datasets (e.g. primary care data only; primary care data, followed by hospital admissions data; primary care data, followed by hospital admissions data, followed by mortality data; primary care data followed by mortality data). The Y axis displays participant number and the X axis shows the median duration (in days) between the identification of participants in each dataset. The length of the bars indicates the number of participants within that group. Thicker bars indicate a group of participants who were only identified in one dataset, thinner bars indicate groups of participants who were identified in more than one dataset over time.
