# Supplementary material for: The Secure Anonymised Information Linkage databank Dementia e-cohort (SAIL-DeC)
Source: Int J Popul Data Sci. 2020 Feb 25;5(1):1121. doi: 10.23889/ijpds.v5i1.1121 (PMC7473277; doi:10.23889/ijpds.v5i1.1121)
Supplement: Supplementary Material [file ijpds-05-01-1121-s001.zip › Supplementary Appendix 8.html]

Event tables


# Event tables

### *Dementia*

#### *Christian*

#### *January 2019*

## Code selection

We have selected codes based on from the UK Biobank algorithm and dementia validation study (unpublished) in conjunction with the WHO ICD 10 browser (apps.who.int/classifications/icd10/browse/2010/en) and the NHS Read Code Browser (https://isd.digital.nhs.uk/trud3/user/guest/group/0/home). We have deliberately included codes with obvious `misspelling’ (for example having a dot where none should be) or ICD 10 codes ending with ‘X’. We have added subtype classification (Alzheimer, Vascular Dementia, Fronto-temporal dementia (FTD), Dementia with Lewis Bodies (DLB) and Unspecific) to each code again based on the UK Biobank algorithm and dementia validation study. We allowed any combination of diagnosistic codes, so people can appear to have several different subtypes of dementia.

All codes that were selected for classification and the total number of people with at least one of the codes are displayed in the following tables. Please be aware that frequency counts of Read V2 codes in the table do not reflect the hierarchical nature of Read V2 coding (for example, counts of E01.. do not include E011.).

### Read V2 codes:

| subtype | code | desc | total\_n |
| --- | --- | --- | --- |
| Alzheimer | Eu00. | [X]Dementia in Alzheimer’s disease | 7609 |
| Alzheimer | Eu000 | [X]Dementia in Alzheimer’s disease with early onset | 223 |
| Alzheimer | Eu001 | [X]Dementia in Alzheimer’s disease with late onset | 1094 |
| Alzheimer | Eu002 | [X]Dementia in Alzheimer’s dis, atypical or mixed type | 2455 |
| Alzheimer | Eu00z | [X]Dementia in Alzheimer’s disease, unspecified | 3208 |
| Alzheimer | F110. | Alzheimer’s disease | 15549 |
| Alzheimer | F1100 | Alzheimer’s disease with early onset | 375 |
| Alzheimer | F1101 | Alzheimer’s disease with late onset | 257 |
| Alzheimer | F112. | Senile degeneration of brain | 215 |
| Alzheimer | Fyu30 | [X]Other Alzheimer’s disease | 7 |
| DLB | Eu025 | [X]Lewy body dementia | 945 |
| DLB | F116. | Lewy body disease | 475 |
| FTD | Eu020 | [X]Dementia in Pick’s disease | 67 |
| FTD | F111. | Pick’s disease | 76 |
| FTD | F118. | Frontotemporal degeneration | 64 |
| unspec | 1461. | H/O: dementia | 4932 |
| unspec | 38C13 | Assessment of psychotic and behavioural symptoms of dementia | 22 |
| unspec | 3AE3. | GDS level 4 - moderate cognitive decline | 26 |
| unspec | 3AE4. | GDS level 5 - moderately severe cognitive decline | <5 |
| unspec | 3AE5. | GDS level 6 - severe cognitive decline | 23 |
| unspec | 3AE6. | GDS level 7 - very severe cognitive decline | <5 |
| unspec | 66h.. | Dementia monitoring | 2000 |
| unspec | 6AB.. | Dementia annual review | 47177 |
| unspec | 8BM02 | Dementia medication review | 1357 |
| unspec | 8CMe0 | Dementia advance care plan | 263 |
| unspec | 8CMG2 | Review of dementia advance care plan | 139 |
| unspec | 8CMZ. | Dementia care plan | 858 |
| unspec | 8CMZ0 | Dementia care plan agreed | 504 |
| unspec | 8CMZ1 | Dementia care plan reviewed | 475 |
| unspec | 8CMZ2 | Dementia care plan declined | 26 |
| unspec | 8CMZ3 | Dementia care plan review declined | 14 |
| unspec | 8CSA. | Dementia advance care plan agreed | 49 |
| unspec | 8Hla. | Referral to dementia care advisor | 123 |
| unspec | 9hD.. | Exception reporting: dementia quality indicators | 116 |
| unspec | 9hD0. | Excepted from dementia quality indicators: Patient unsuitable | 3992 |
| unspec | 9hD1. | Excepted from dementia quality indicators: Informed dissent | 1331 |
| unspec | 9Ou.. | Dementia monitoring administration | 318 |
| unspec | 9Ou1. | Dementia monitoring first letter | 4391 |
| unspec | 9Ou2. | Dementia monitoring second letter | 1562 |
| unspec | 9Ou3. | Dementia monitoring third letter | 751 |
| unspec | 9Ou4. | Dementia monitoring verbal invite | 159 |
| unspec | 9Ou5. | Dementia monitoring telephone invite | 376 |
| unspec | A411. | Jakob-Creutzfeldt disease | 11 |
| unspec | A4110 | Sporadic Creutzfeldt-Jakob disease | 9 |
| unspec | E00.. | Senile and presenile organic psychotic conditions | 24228 |
| unspec | E000. | Uncomplicated senile dementia | 3108 |
| unspec | E001. | Presenile dementia | 472 |
| unspec | E0010 | Uncomplicated presenile dementia | 14 |
| unspec | E0011 | Presenile dementia with delirium | 17 |
| unspec | E0012 | Presenile dementia with paranoia | 36 |
| unspec | E0013 | Presenile dementia with depression | 48 |
| unspec | E001z | Presenile dementia NOS | 58 |
| unspec | E002. | Senile dementia with depressive or paranoid features | 97 |
| unspec | E0020 | Senile dementia with paranoia | 161 |
| unspec | E0021 | Senile dementia with depression | 259 |
| unspec | E002z | Senile dementia with depressive or paranoid features NOS | 22 |
| unspec | E003. | Senile dementia with delirium | 173 |
| unspec | E012. | Other alcoholic dementia | 227 |
| unspec | E0120 | Chronic alcoholic brain syndrome | 21 |
| unspec | E02y1 | Drug-induced dementia | 8 |
| unspec | E041. | Dementia in conditions EC | 608 |
| unspec | Eu012 | [X]Subcortical vascular dementia | 66 |
| unspec | Eu013 | [X]Mixed cortical and subcortical vascular dementia | 439 |
| unspec | Eu02. | [X]Dementia in other diseases classified elsewhere | 242 |
| unspec | Eu021 | [X]Dementia in Creutzfeldt-Jakob disease | 7 |
| unspec | Eu022 | [X]Dementia in Huntington’s disease | 41 |
| unspec | Eu023 | [X]Dementia in Parkinson’s disease | 723 |
| unspec | Eu02y | [X]Dementia in other specified diseases classif elsewhere | 19 |
| unspec | Eu02z | [X] Unspecified dementia | 13394 |
| unspec | Eu041 | [X]Delirium superimposed on dementia | 51 |
| unspec | Eu106 | [X]Mental and behavioural disorders due to use of alcohol: amnesic syndrome | 101 |
| unspec | Eu107 | [X]Mental and behavioural disorders due to use of alcohol: residual and late-onset psychotic disorder | 125 |
| unspec | F11x7 | Cerebral degeneration due to Jakob - Creutzfeldt disease | 9 |
| unspec | F11x9 | Cerebral degeneration in Parkinson’s disease | 9 |
| unspec | F11y2 | Corticobasal degeneration | 37 |
| Vascular | E004. | Arteriosclerotic dementia | 2427 |
| Vascular | E0040 | Uncomplicated arteriosclerotic dementia | 88 |
| Vascular | E0041 | Arteriosclerotic dementia with delirium | 6 |
| Vascular | E0042 | Arteriosclerotic dementia with paranoia | 16 |
| Vascular | E0043 | Arteriosclerotic dementia with depression | 32 |
| Vascular | E004z | Arteriosclerotic dementia NOS | 108 |
| Vascular | Eu01. | [X]Vascular dementia | 15621 |
| Vascular | Eu010 | [X]Vascular dementia of acute onset | 22 |
| Vascular | Eu011 | [X]Multi-infarct dementia | 429 |
| Vascular | Eu01y | [X]Other vascular dementia | 138 |
| Vascular | Eu01z | [X]Vascular dementia, unspecified | 543 |
| Vascular | F11x2 | Cerebral degeneration due to cerebrovascular disease | 32 |
| Vascular | F21y2 | Binswanger’s disease | 33 |

### ICD 9 and 10 codes:

| subtype | code | desc | total\_n |
| --- | --- | --- | --- |
| Alzheimer | 3310 | Alzheimer s disease | 995 |
| Alzheimer | F000 | Dementia in alzheimer disease with early onset | 1319 |
| Alzheimer | F001 | Dementia in alzheimer disease with late onset | 5571 |
| Alzheimer | F002 | Dementia in alzheimer disease atypical or mixed type | 2826 |
| Alzheimer | F009 | Dementia in alzheimer disease unspecified | 15617 |
| Alzheimer | G300 | Alzheimer disease with early onset | 1042 |
| Alzheimer | G301 | Alzheimer disease with late onset | 3223 |
| Alzheimer | G308 | Other alzheimer disease | 2437 |
| Alzheimer | G309 | Alzheimer disease unspecified | 29791 |
| FTD | 3311 | Pick s disease | <5 |
| FTD | F020 | Dementia in pick disease | 485 |
| FTD | G310 | Circumscribed brain atrophy | 804 |
| unspec | 0461 | Jakob-creutzfeldt disease | 5 |
| unspec | 2900 | Senile dementia simple type | 1220 |
| unspec | 2901 | Presenile dementia | 213 |
| unspec | 2902 | Senile dementia depressed or paranoid type | 119 |
| unspec | 2903 | Senile dementia with acute confusional state | <5 |
| unspec | 3312 | Senile degeneration of brain | 5 |
| unspec | A810 | Creutzfeldt-jakob disease | 81 |
| unspec | F021 | Dementia in creutzfeldt-jakob disease | 29 |
| unspec | F022 | Dementia in huntington disease | 85 |
| unspec | F023 | Dementia in parkinson disease | 2466 |
| unspec | F024 | Dementia in human immunodeficiency virus [hiv] disease | 5 |
| unspec | F028 | Dementia in other specified diseases classified elsewhere | 1711 |
| unspec | F03 | Unspecified dementia | 26572 |
| unspec | F03. | NA | 19 |
| unspec | F030 | NA | 13956 |
| unspec | F031 | NA | <5 |
| unspec | F033 | NA | <5 |
| unspec | F034 | NA | <5 |
| unspec | F039 | NA | <5 |
| unspec | F03X | NA | 64301 |
| unspec | F051 | Delirium superimposed on dementia | 1628 |
| unspec | F106 | Mental and behavioural disorders due to use of alcohol | 672 |
| unspec | G311 | Senile degeneration of brain not elsewhere classified | 461 |
| unspec | G318 | Other specified degenerative diseases of nervous system | 2695 |
| Vascular | 2904 | Arteriosclerotic dementia | 424 |
| Vascular | F010 | Vascular dementia of acute onset | 765 |
| Vascular | F011 | Multi-infarct dementia | 5251 |
| Vascular | F012 | Subcortical vascular dementia | 220 |
| Vascular | F013 | Mixed cortical and subcortical vascular dementia | 458 |
| Vascular | F018 | Other vascular dementia | 380 |
| Vascular | F019 | Vascular dementia unspecified | 23958 |
| Vascular | I673 | Progressive vascular leukoencephalopathy | 66 |

## Descriptives

129632 people had at least one diagnostic code in at least one of the datasets; 101654 had a hospital code, 52198 had a code in the mortality data and 78828 had a GP code. The following figure shows the year of the first code that was found for any person classified positive using (a) all codes combined, (b) only codes from hospital admissions, (c) only codes from the mortality statistics and (d) only codes from GP consultations.
