# Supplementary material for: The Secure Anonymised Information Linkage databank Dementia e-cohort (SAIL-DeC)
Source: Int J Popul Data Sci. 2020 Feb 25;5(1):1121. doi: 10.23889/ijpds.v5i1.1121 (PMC7473277; doi:10.23889/ijpds.v5i1.1121)
Supplement: Supplementary Material [file ijpds-05-01-1121-s001.zip › Supplementary Appendix 9.html]

Event tables


# Event tables

### *Alcohol dependence*

#### *Christian*

#### *January 2019*

## Code selection

We have selected codes based on Thompson, A et al. Drug therapy for alcohol dependence in primary care in the UK: A Clinical Practice Research Datalink study. PLoS ONE. 2017; 12(3) e0173272 and Carr MJ et al. Premature death among primary care patients with a history of self-harm. Ann Fam Med. 2017; 15(3): 246-254 (list at https://clinicalcodes.rss.mhs.man.ac.uk/medcodes/article/41) in conjunction with the WHO ICD 10 browser (apps.who.int/classifications/icd10/browse/2010/en) and the NHS Read Code Browser (https://isd.digital.nhs.uk/trud3/user/guest/group/0/home). We have deliberately included codes with obvious `misspelling’ (for example having a dot where none should be) or ICD 10 codes ending with ‘X’.

All codes that were selected for classification and the total number of people with at least one of the codes are displayed in the following tables. Please be aware that frequency counts of Read V2 codes in the table do not reflect the hierarchical nature of Read V2 coding (for example, counts of E01.. do not include E011.).

### Read V2 codes:

| code | desc | total\_n |
| --- | --- | --- |
| 1366. | Very heavy drinker - >9u/day | 7407 |
| 136Q. | Very heavy drinker | 703 |
| 136T. | Harmful alcohol use | 1082 |
| 1462. | H/O: alcoholism | 1706 |
| 1B1c. | Alcohol induced hallucinations | 39 |
| 66e0. | Alcohol abuse monitoring | 449 |
| 7P221 | Delivery of rehabilitation for alcohol addiction | 22 |
| 8BA8. | Alcohol detoxification | 2663 |
| 8CAv. | Advised to contact primary care alcohol worker | 13 |
| 8G32. | Aversion therapy - alcoholism | 52 |
| 8H35. | Admitted to alcohol detoxification centre | 221 |
| 8HkG. | Referral to specialist alcohol treatment service | 57 |
| 8IAF. | Brief intervention for excessive alcohol consumption declined | 53 |
| 8IAJ. | Declined referral to specialist alcohol treatment service | 97 |
| 8IAt. | Extended intervention for excessive alcohol consumption declined | 20 |
| 9k1B. | Extended intervention for excessive alcohol consumption completed | 6 |
| 9NN2. | Under care of community alcohol team | 176 |
| C1505 | Alcohol-induced pseudo-Cushing’s syndrome | <5 |
| E01.. | Alcoholic psychoses | 99 |
| E010. | Alcohol withdrawal delirium | 355 |
| E011. | Alcohol amnestic syndrome | 20 |
| E0110 | Korsakov’s alcoholic psychosis | 239 |
| E0111 | Korsakov’s alcoholic psychosis with peripheral neuritis | 14 |
| E0112 | Wernicke-Korsakov syndrome | 90 |
| E011z | Alcohol amnestic syndrome NOS | <5 |
| E012. | Other alcoholic dementia | 227 |
| E0120 | Chronic alcoholic brain syndrome | 21 |
| E013. | Alcohol withdrawal hallucinosis | 27 |
| E014. | Pathological alcohol intoxication | 51 |
| E015. | Alcoholic paranoia | 15 |
| E01y. | Other alcoholic psychosis | 67 |
| E01y0 | Alcohol withdrawal syndrome | 1703 |
| E01yz | Other alcoholic psychosis NOS | <5 |
| E01z. | Alcoholic psychosis NOS | 26 |
| E23.. | Alcohol dependence syndrome | 18360 |
| E230. | Acute alcoholic intoxication in alcoholism | 499 |
| E2300 | Acute alcoholic intoxication, unspecified, in alcoholism | 33 |
| E2301 | Continuous acute alcoholic intoxication in alcoholism | 9 |
| E2302 | Episodic acute alcoholic intoxication in alcoholism | 13 |
| E2303 | Acute alcoholic intoxication in remission, in alcoholism | <5 |
| E230z | Acute alcoholic intoxication in alcoholism NOS | 41 |
| E231. | Chronic alcoholism | 776 |
| E2310 | Unspecified chronic alcoholism | 47 |
| E2311 | Continuous chronic alcoholism | 212 |
| E2312 | Episodic chronic alcoholism | 137 |
| E2313 | Chronic alcoholism in remission | 135 |
| E231z | Chronic alcoholism NOS | 197 |
| E23z. | Alcohol dependence syndrome NOS | 879 |
| E2503 | Nondependent alcohol abuse in remission | 89 |
| Eu10. | [X]Mental and behavioural disorders due to use of alcohol | 106 |
| Eu100 | [X]Mental and behavioural disorders due to use of alcohol: acute intoxication | 143 |
| Eu101 | [X]Mental and behavioural disorders due to use of alcohol: harmful use | 59 |
| Eu102 | [X]Mental and behavioural disorders due to use of alcohol: dependence syndrome | 563 |
| Eu103 | [X]Mental and behavioural disorders due to use of alcohol: withdrawal state | 108 |
| Eu104 | [X]Mental and behavioural disorders due to use of alcohol: withdrawal state with delirium | 104 |
| Eu105 | [X]Mental and behavioural disorders due to use of alcohol: psychotic disorder | 93 |
| Eu106 | [X]Mental and behavioural disorders due to use of alcohol: amnesic syndrome | 101 |
| Eu107 | [X]Mental and behavioural disorders due to use of alcohol: residual and late-onset psychotic disorder | 125 |
| Eu108 | [X]Alcohol withdrawal-induced seizure | 227 |
| Eu10y | [X]Mental and behavioural disorders due to use of alcohol: other mental and behavioural disorders | <5 |
| Eu10z | [X]Mental and behavioural disorders due to use of alcohol: unspecified mental and behavioural disorder | <5 |
| F11x0 | Cerebral degeneration due to alcoholism | 121 |
| F1440 | Cerebellar ataxia due to alcoholism | 43 |
| F25B. | Alcohol-induced epilepsy | 48 |
| F375. | Alcoholic polyneuropathy | 170 |
| F3941 | Alcoholic myopathy | 34 |
| G555. | Alcoholic cardiomyopathy | 278 |
| G8523 | Oesophageal varices in alcoholic cirrhosis of the liver | 57 |
| J610. | Alcoholic fatty liver | 768 |
| J611. | Acute alcoholic hepatitis | 415 |
| J612. | Alcoholic cirrhosis of liver | 2208 |
| J6120 | Alcoholic fibrosis and sclerosis of liver | 20 |
| J613. | Alcoholic liver damage unspecified | 2429 |
| J6130 | Alcoholic hepatic failure | 92 |
| J617. | Alcoholic hepatitis | 387 |
| J6170 | Chronic alcoholic hepatitis | 29 |
| J6710 | Alcohol-induced chronic pancreatitis | 130 |
| SLH3. | Alcohol deterrent poisoning | 5 |
| SM001 | Denatured alcohol causing toxic effect | <5 |
| U60H3 | [X]Alcohol deterrents causing adverse effects in therapeutic use | 0 |

### ICD 9 and 10 codes:

| code | desc | total\_n |
| --- | --- | --- |
| 291 | Alcoholic psychoses | 0 |
| 2910 | Delirium tremens | <5 |
| 2911 | Korsakov s psychosis alcoholic | 0 |
| 2912 | Other alcoholic dementia | <5 |
| 2913 | Other alcoholic hallucinosis | <5 |
| 2914 | Pathological drunkenness | 0 |
| 2915 | Alcoholic jealousy | 0 |
| 2918 | Other | 5 |
| 2919 | Unspecified | 0 |
| 303 | Alcohol dependence syndrome | 117 |
| 4255 | Alcoholic cardiomyopathy | 15 |
| 5710 | Alcoholic fatty liver | 12 |
| E244 | Alcohol-induced pseudo-Cushing syndrome | 6 |
| F10. | NA | 46 |
| F101 | Mental and behavioural disorders due to use of alcohol | 14063 |
| F102 | Mental and behavioural disorders due to use of alcohol | 15060 |
| F103 | Mental and behavioural disorders due to use of alcohol | 4561 |
| F104 | Mental and behavioural disorders due to use of alcohol | 699 |
| F105 | Mental and behavioural disorders due to use of alcohol | 237 |
| F106 | Mental and behavioural disorders due to use of alcohol | 672 |
| F107 | Mental and behavioural disorders due to use of alcohol | 571 |
| F108 | Mental and behavioural disorders due to use of alcohol | 47 |
| F109 | Mental and behavioural disorders due to use of alcohol | 589 |
| F10U | NA | <5 |
| F10X | NA | 101 |
| G312 | Degeneration of nervous system due to alcohol | 754 |
| G721 | Alcoholic myopathy | 92 |
| I426 | Alcoholic cardiomyopathy | 548 |
| K70 | Alcoholic liver disease | 0 |
| K700 | Alcoholic fatty liver | 629 |
| K701 | Alcoholic hepatitis | 865 |
| K702 | Alcoholic fibrosis and sclerosis of liver | 68 |
| K703 | Alcoholic cirrhosis of liver | 4791 |
| K704 | Alcoholic hepatic failure | 1482 |
| K709 | Alcoholic liver disease unspecified | 7279 |
| K70D | NA | <5 |
| K70X | NA | <5 |
| K852 | Alcohol-induced acute pancreatitis | 147 |
| K860 | Alcohol-induced chronic pancreatitis | 949 |

## Descriptives

49544 people had at least one diagnostic code in at least one of the datasets. 30595 people had a code in hospital admissions data, 3873 in mortality data and 31819 in primary care data. The following figure shows the year of the first code that was found for any person classified positive using (a) all codes combined, (b) only codes from hospital admissions data, (c) only codes from the mortality data and (d) only codes from primary care data.
